# Between-tumor and within-tumor heterogeneity in invasive potential

**DOI:** 10.1371/journal.pcbi.1007464

**Published:** 2020-01-21

**Authors:** Veena Padmanaban, Yohannes Tsehay, Kevin J. Cheung, Andrew J. Ewald, Joel S. Bader

**Affiliations:** 1 Center for Cell Dynamics and Department of Cell Biology, Johns Hopkins University School of Medicine, Baltimore, Maryland, United States of America; 2 High-Throughout Biology Center and Department of Biomedical Engineering, Johns Hopkins University, Baltimore, Maryland, United States of America; 3 Cancer Invasion and Metastasis Program, Department of Oncology, Sidney Kimmel Comprehensive Cancer Center, Johns Hopkins University, Baltimore, Maryland, United States of America; H. Lee Moffitt Cancer Center and Research Institute, UNITED STATES

## Abstract

For women with access to healthcare and early detection, breast cancer deaths are caused primarily by metastasis rather than growth of the primary tumor. Metastasis has been difficult to study because it happens deep in the body, occurs over years, and involves a small fraction of cells from the primary tumor. Furthermore, within-tumor heterogeneity relevant to metastasis can also lead to therapy failures and is obscured by studies of bulk tissue. Here we exploit heterogeneity to identify molecular mechanisms of metastasis. We use “organoids”, groups of hundreds of tumor cells taken from a patient and grown in the lab, to probe tumor heterogeneity, with potentially thousands of organoids generated from a single tumor. We show that organoids have the character of biological replicates: within-tumor and between-tumor variation are of similar magnitude. We develop new methods based on population genetics and variance components models to build between-tumor and within-tumor statistical tests, using organoids analogously to large sibships and vastly amplifying the test power. We show great efficiency for tests based on the organoids with the most extreme phenotypes and potential cost savings from pooled tests of the extreme tails, with organoids generated from hundreds of tumors having power predicted to be similar to bulk tests of hundreds of thousands of tumors. We apply these methods to an association test for molecular correlates of invasion, using a novel quantitative invasion phenotype calculated as the spectral power of the organoid boundary. These new approaches combine to show a strong association between invasion and protein expression of Keratin 14, a known biomarker for poor prognosis, with *p* = 2 × 10^−45^ for within-tumor tests of individual organoids and *p* < 10^−6^ for pooled tests of extreme tails. Future studies using these methods could lead to discoveries of new classes of cancer targets and development of corresponding therapeutics. All data and methods are available under an open source license at https://github.com/baderzone/invasion_2019.

## Author summary

For women with access to healthcare and early detection, breast cancer deaths are caused primarily by metastasis rather than growth of the primary tumor. Metastasis has been difficult to study because it happens deep in the body, occurs over years, and involves a small fraction of cells from the primary tumor. Furthermore, individual cells within a tumor can behave very differently, leading to failures of therapies. Here we exploit heterogeneity to develop new methods to identify molecular mechanisms of metastasis. We use “organoids”, groups of hundreds tumor cells taken from a patient and grown in the lab. Thousands of organoids can be generated from a single tumor sample to probe different regions and amplify the amount of information provided. Organoids provide information about metastasis because they vary in their ability to invade the growth medium. We introduce a new phenotype for invasion obtained by converting the boundary of an organoid into a frequency spectrum, then summing the power across all frequencies. We analyze this metastasis-related phenotype by adapting methods from population genetics that compare the most extreme siblings in a family. We analogously compare the most invasive vs. least invasive organoids from each tumor. Power calculations suggest that studies of 50–100 individuals, with 100-1000 organoids generated from each, could reveal DNA mutations and aberrant gene expression associated with invasion. We validate this approach by demonstrating strong statistical significance between invasion and protein expression of Keratin 14, a known biomarker for poor prognosis. Future studies using these methods could lead to discoveries of new classes of cancer targets and development of corresponding therapeutics.

## Introduction

Metastasis, rather than growth of the primary tumor, is the major cause of breast cancer mortality in developed nations [1]. Estimates for the United States for 2019 are 271,270 new invasive breast cancer cases and 42,260 deaths [2]. For women with access to healthcare, five-year survival rates are approximately 95% for localized cancer, 85% for regional cancer, and 35% for distant-stage disease [1, 3].

Despite its importance in driving mortality, metastasis remains difficult to target clinically. Most therapies aim to suppress proliferation or eliminate proliferating cells rather than slow or halt specific stages of the metastatic process, such as invasion, dissemination, and seeding of secondary micro-metastases. These processes remain challenging to study because they occur deep in the body, can take years or decades to develop, and may arise in part from genetic and genomic heterogeneity of tumor cells [4]. Recent studies, for example, suggest that different tumor cell states are responsible for proliferation versus dissemination [5–8].

Organotypic cell culture has provided powerful new systems for studying metastasis [9]. Organoids are groups of hundreds of cells that self-organize in three-dimensional medium into organ-like structures and have phenotypes that are proxies for the corresponding *in vivo* system. Organoids derived from mammary tissues and tumors have been used to represent multiple stages of breast cancer metastasis, including invasion [10] and dissemination [11]. Invasive structures observed within organoid generated from murine and human human tumors have properties similar to *in vivo* tumor-stromal boundaries in genetically engineered mouse models and in diverse sub-types of human breast tumors [10]. Cell types within these invasive structures are distinguished by Keratin 14 expression: Keratin 14 positive (K14+) cells lead collective invasion strands, while Keratin 14 negative (K14−) cells form the bulk of the organoid or primary tumor [10]. These results mirror clinical findings of increased mortality from breast tumors expressing higher levels of K14 [12].

Heterogeneity exists both between tumors and within tumors. Measurements of the tumor bulk can obscure within-tumor heterogeneity, losing the ability to characterize smaller cell populations responsible for metastatic phenotypes or therapy resistance. In our context, however, heterogeneity can be exploited by probing variation in phenotype, genotype, and gene expression across different organoids generated from a single tumor. We propose that between-tumor and within-tumor variation are analogous to between-family and within-family variation in population genetics, with organoids generated from a single tumor corresponding to siblings within a large family.

We use this insight to quantitatively analyze organoid behavior with methods from population genetics. Within-tumor methods are particularly powerful because thousands of organoids can be generated from a single tumor, their shared genetic and environmental background can be subtracted as a common baseline, and pooled tests of the most extreme organoids are very efficient. As with genome-wide association studies, genetic and genomic associations identified by organoid population genetics could then be validated experimentally by directed perturbations. This combination of population-based organoid studies of invasion phenotypes and genetic/genomic perturbations could lead to new classes of targets for metastatic breast cancer and other invasive cancers.

Statistical genetics methods benefit from having quantitative rather than qualitative phenotypes. Invasion and other metastasis-related phenotypes have generally been qualitative, however, based on visual sorting into categories often denoted ‘+’, ‘++’, ‘+++’. Reproducible methods for automated scoring, similar to the quantitative growth rates used for proliferation phenotypes, have additional value in permitting more systematic, genome-scale studies that complement detailed phenotyping of individual genetic or chemical perturbations.

Fractal dimension calculations can provide features for image classification [13] and have been suggested for classifying macroscopic tumor-stromal boundaries [14, 15]. Fractal dimensions can also be related to boundary fluctuations predicted by stochastic models of tumor growth [16]. Organoids have a natural smallest length scale of a single cell, however, and are too small to permit the scaling over several orders of magnitude required to calculate a fractal dimension. Fourier modes of one-dimensional boundary signatures have also been used to characterize shape, with the amplitude-weighted mean of the inverse of the frequency as a feature, together with other measures of boundary curvature and compactness [14, 15].

Here we define a quantitative phenotype for the invasive behavior of organoids generated from human breast tumor tissue. The mathematical technique is motivated by methods developed for analysis of biological shape using spectral transforms of a boundary [17]. These methods map two-dimensional boundaries to a curve parameterized by contour length, then perform Fourier transforms for each coordinate separately to arrive at a lossless representation. The spectral power is invariant to translation or rotation of the organoid boundary in the imaging plane. Similar methods have been useful for characterizing the behavior of isolated single cells [18].

We provide a formal description of the mathematical procedure, including normalization for organoid size, smoothing of possible pixelation artifacts, and recognizing high-curvature invasive boundaries. Organoids generated from distinct tumor samples are analyzed using these methods. A variance components model, the standard framework for quantitative traits in statistical genetics [19–21], defines contributions to invasiveness due to heterogeneity between tumors and due to heterogeneity within a single tumor. Heterogeneity is often considered a difficulty in cancer studies; here we describe how it can be exploited as a source of information in population-based studies. The variance components framework suggests that the population sizes that will be required to conduct well-powered association studies at genome-wide significance are highly feasible within single laboratories.

We conduct such an analysis for Keratin 14, using immunofluorescence to characterize protein expression within each organoid for correlation tests with invasion. We present results for tests based on all organoids, on the most and least invasive organoids from each tumor, and on pools of the extreme tails. These tests are all significant. We conclude with implications for population-based studies of cancer metastasis.

## Materials and methods

### Ethics statement

The use of human tumor specimens was approved by JHM-IRB X as study number NA_00077976, “Molecular Regulation of Breast Cancer Invasion”. The IRB determined that the use of de-identified tumor specimens is not human subjects research for which IRB review is required.

### Clinical cohort

A total of 60 human breast tumor specimens were obtained as surgical samples from the Cooperative Human Tissue Network in accordance with a Johns Hopkins School of Medicine IRB acknowledged exempt study design. Basic demographic information (age, ethnicity, sex), entry date, and type of sample (primary tumor, recurrence) was available for every specimen. A total of 823 organoids were generated from 52 of the specimens. Most statistical analyses were limited to 47 specimens with 5 or more organoids, corresponding to 811 organoids. While all samples were consented for organoid generation and analysis, consent for continued access to medical records was not consistently requested; consequently, this study was not designed for analysis of outcomes. For more detail about the clinical cohort, see S1 Table.

### Organoid generation

Breast tumor specimens were processed individually to generate organoids, with approximately 300–500 cells per organoids, according to published protocols [10, 22]. The organoids were cultured in collagen I for six days and then imaged. Each organoid was imaged using differential interference contrast (DIC) microscopy and at identical resolution and position using epifluorescence for Keratin-14 immunofluorescence (Biolegend Cat. No. 005301). Epifluorescence and DIC both yielded 1040×1388 pixel images. Microscope optics defined a resolution of 0.51190476 *μ*m per pixel (full precision based on stated values), corresponding to a 532.4×710.5 *μ*m field of view. A total of 823 organoids were imaged, corresponding to an average of 16 organoids per tumor.

Organoids in DIC images were manually traced using ImageJ [23] to define their boundaries as pairs of points {*x*_*v*_, *y*_*v*_} for *v* ∈ 0…*V* − 1. The total number of points *V* depended on the manual outline, and the spacing between adjacent points was variable. The organoid boundaries were then used to define a spatial mask to extract pixel intensities from the paired epifluorescence image. Pixel intensities were recorded as integers in the range 0 (no fluorescence) through 255 (saturation) and scaled to the closed interval [0, 1] by dividing by 255. The scaled values within the organoid boundary were summed. The total K14 intensity was then defined as the sum divided by 1,443,520, the number of pixels in the image, and the mean K14 intensity was defined as the sum divided by the area of the organoid in pixels. As a final step to ensure robust analysis unaffected by details of the K14 distribution or epifluorescence dynamic range, we calculated rank-normalized values for total K14 by replacing each of the *n* = 823 values by its rank in the range 1…*n*, with ties assigned the average value, subtracting 0.5 and then dividing by *n* to obtain a value in the open interval (0, 1). The same procedure was used to calculate rank-normalized mean K14.

### Spectral power

Boundary coordinates were analyzed using custom software, Ibis2d (see S1 File, provided under an open source license), implementing a spectral transform method. First, the overall contour length *L* of each organoid was calculated as

$$\begin{array}{c} L=\sum_{v=0}^{V-1}\sqrt{{\left(x_{v}-x_{v-1}\right)}^{2}+{\left(y_{v}-y_{v-1}\right)}^{2}}. \end{array}$$ (1)

The total number of boundary points used for manual segmentation is denoted *V*. The index *v* labels these points with periodic boundary conditions, (*x*_−1_, *y*_−1_) ≡ (*x*_*V*−1_, *y*_*V*−1_). Next, *M* points were chosen along the contour with equal spacing *L*/*M* between adjacent points. A new boundary of equally-spaced points (*x*_*j*_, *y*_*j*_) for integer *j* ∈ {0, 1, …, *M* − 1} was then calculated using linear interpolation of the original boundary by Python scipy.interpolate.interp1d. Results shown used *M* = 256 total points.

Areas for organoids were calculated using the shoelace formula applied to the interpolation points. An effective diameter was calculated as $2\times \sqrt{\text{area}/\pi }$. Normalized ranks were calculated for organoid size; because the area and effective diameter are monotonic, they yield the same ranks for size.

We then used Python numpy.fft.rfft to perform a fast Fourier transform for real-valued parametric curve {*x*_*j*_, *y*_*j*_} to obtain $\left\{{\hat{x}}_{k},{\hat{y}}_{k}\right\}$ for integer *k* ∈ {0, 1, …, (*M*/2) + 1}, with

$$\begin{array}{c} {\hat{x}}_{k}=\sum_{j=0}^{M-1}e^{-2\pi ijk/M}x_{j}. \end{array}$$ (2)

An analogous transform provided ${\hat{y}}_{k}$. The terms $\left\{{\hat{x}}_{k},{\hat{y}}_{k}\right\}$ are in general complex valued, except for the zero-frequency terms $\left({\hat{x}}_{0},{\hat{y}}_{0}\right)$ which, when normalized by *M*, give the boundary centroid. Note that ${\hat{x}}_{k}={\hat{x}}_{k\;\text{mod}\;M}$, so that ${\hat{x}}_{-1}={\hat{x}}_{M-1}$ and so on; the entire Fourier spectrum has frequencies *k* ∈ {0, 1, …, *M* − 1} or equivalently *k* ∈ {0, ±1, …, ±*M*/2} for even-valued *M*. Because (*x*_*k*_, *y*_*k*_) and (*x*_−*k*_, *y*_−*k*_) are complex conjugates, only the *k* ≥ 0 Fourier terms are computed.

The spectral power *P*_*k*_ at frequency *k* is

$$\begin{array}{c} P_{k}={\hat{x}}_{k}\cdot {\hat{x}}_{-k}+{\hat{y}}_{k}\cdot {\hat{y}}_{-k}=|{\hat{x}}_{k}{|}^{2}+{\left|{\hat{y}}_{k}\right|}^{2} \end{array}$$ (3)

and characterizes the fluctuations in the parametric curve describing the boundary. Each term in *P*_*k*_ is invariant to rotation in the imaging plane. The term *P*_0_, representing the boundary centroid, is discarded to make the power invariant to translation in the imaging plane. Thus the total spectral power $\sum_{k=1}^{M/2}P_{k}$ provides a measure of boundary fluctuations that is invariant to translations and rotations in the imaging plane.

We then normalized the spectral power by the power of the first mode, *P*_1_, to make the sum scale-invariant, introduced a smoothing transformation to correct for pixelation, and introduced a derivative transformation that represents curvature (see S1 Methods). We define the resulting sum as the weighted spectral power, *w*,

$$\begin{array}{c} w\equiv \sum_{k=2}^{M/2}{\left(M/\pi \right)}^{2}\;{\text{sin}}^{2}\left(\pi k/M\right)\;{\text{cos}}^{2}\left(\pi k/M\right)P_{k}/P_{1}. \end{array}$$ (4)

We found that this weighted form, compared to unweighted power, provided slightly improved agreement between organoids ranked qualitatively by visual inspection and quantitatively by spectral power (see S1 File, software repository to generate results without smoothing and weighting filters). More sophisticated smoothers are possible [24], but agreement between results with *M* = 256 and *M* = 128 Fourier components indicates that smoothing with $\bar{x}$ and $\bar{y}$ is sufficient. Although sin^2^(*πk*/*M*) cos^2^(*πk*/*M*) = (1/4) sin^2^(2*πk*/*M*), we retain the two-factor form to make the origin of each term clear. At small *k*, the weight has the form of a low-pass Gaussian filter with a weight of *k*^2^ (see S1 Methods).

The full data set **D** is the set of values {*w*_*ti*_}, with index *t* ∈ {1, 2, …, *T*} denoting the tumor, *i* ∈ {1, 2, …, *N*_*t*_} denoting an individual organoid generated from tumor *t*, and *N*_*t*_ denoting the number of organoids generated from tumor *t*. The total number of organoids is denoted *N*,

$$\begin{array}{c} N=\sum_{t=1}^{T}N_{t}. \end{array}$$ (5)

Calculations for 823 organoids required 5 to 7 min (MacOS 10.13, 3.1 GHz Intel Core i7, 16 GB memory).

### Bootstrapped Bayesian model selection

The invasiveness of an organoid is modeled as a random variable from a particular probability distribution. Model selection corresponds to identifying the form of a probability distribution that could have generated the observed data; it also usually involves estimating the values of parameters required by the distribution. We consider three possible models describing the variation of organoid invasiveness within and between tumors. The null model, *M*_0_, assumes an equal mean, *μ*_0_, and variance, $\sigma_{0}^{2}$, for each tumor. The first alternative model, *M*_1_, incorporates an independent mean, *μ*_*t*_, for each tumor, but retains a shared within-group variance $\sigma_{W}^{2}$. The second alternative model, *M*_2_, incorporates both a tumor-dependent mean, *μ*_*t*_, and a tumor-dependent variance, $\sigma_{t}^{2}$.

Bayesian model selection is used to select the most likely model. The posterior probability of a model *M* given observed data **D** depending on parameters Θ is

$$\begin{array}{c} \text{Pr}(M|D)=\text{Pr}(D|M)\;\text{Pr}(M)/\text{Pr}(D) \end{array}$$ (6)

$$\begin{array}{ccc} & = & \frac{\int_{\Theta }\text{Pr}\left(D|\Theta \right)\;\text{Pr}\left(\Theta |M\right)\;\text{Pr}\left(M\right)}{\sum_{M^{\prime }}\text{Pr}\left(M^{\prime }|D\right)}. \end{array}$$ (7)

where *M*′ sums over the models being considered. We use Schwarz’s Bayesian information criterion asymptotic limit for the logarithm of the numerator [25] to define the model score *S*_*M*_,

$$\begin{array}{c} \text{ln}\;\text{Pr}\left(D|M\right)∼\text{ln}\;\text{Pr}\left(D|\tilde{\Theta },M\right)-\left(1/2\right)\sum_{\theta \in \Theta }\text{ln}\;n_{\theta }\equiv S_{M}, \end{array}$$ (8)

where $\tilde{\Theta }$ are the maximum likelihood parameters,

$$\begin{array}{c} \tilde{\Theta }=\text{arg}\;\underset{\Theta }{\text{max}}\;\text{Pr}\left(D|\Theta ,M\right), \end{array}$$ (9)

*θ* is one of the parameters, and *n*_*θ*_ is the number of observations used to obtain $\tilde{\theta }$. With a uniform prior over models, the posterior probability of each model is

$$\begin{array}{c} \text{Pr}\left(M|D\right)=\text{exp}\left(S_{M}\right)/\sum_{M^{\prime }}\text{exp}\left(S_{M^{\prime }}\right). \end{array}$$ (10)

In practice, to avoid numeric underflow, we identify the most likely model $\tilde{M}$,

$$\begin{array}{c} \tilde{M}=\text{arg}\;\underset{M}{\text{max}}\;S_{M}, \end{array}$$ (11)

define $\Delta S_{M}=S_{M}-S_{\tilde{M}}$, and then calculate posterior probabilities as

$$\begin{array}{c} \text{Pr}\left(M|D\right)=\text{exp}\left(\Delta S_{M}\right)/\sum_{M^{\prime }}\text{exp}\left(\Delta S_{M^{\prime }}\right). \end{array}$$ (12)

An important additional consideration for model selection is the scale of the invasiveness data. Many biological processes involve multiplicative noise, generating a log-normal distribution (the exponential of a normally distributed random variable) rather than a normal distribution arising from additive noise. Applying a logarithmic transform, as is usually done when comparing gene or protein expression levels, usually works well to recover normally-distributed data. This is valuable because statistical models and hypothesis tests often assume normally distributed data, and in many cases further assume that variance can be modeled as a single parameter independent of group.

To assess the arithmetic scale and the logarithmic scale via model selection, the data **D** are defined as {*y*_*ti*_}, with *y*_*ti*_ ≡ *w*_*ti*_ for the arithmetic scale and *y*_*ti*_ ≡ log_10_ *w*_*ti*_ for the logarithmic scale. The probabilities of the observed data for the three models are

$$\begin{array}{c} M_{0}:\;\;\text{Pr}\left(D|\mu_{0},\sigma_{0}^{2}\right)=\prod_{t=1}^{T}\prod_{i=1}^{N_{t}}{\left(2\pi \sigma_{0}^{2}\right)}^{-1/2}\text{exp}\left[-{\left(y_{it}-\mu_{0}\right)}^{2}/2\sigma_{0}^{2}\right]; \end{array}$$ (13)

$$\begin{array}{c} M_{1}:\;\;\text{Pr}\left(D|\left\{\mu_{t}\right\},\sigma_{W}^{2}\right)=\prod_{t=1}^{T}\prod_{i=1}^{N_{t}}{\left(2\pi \sigma_{W}^{2}\right)}^{-1/2}\text{exp}\left[-{\left(y_{it}-\mu_{t}\right)}^{2}/2\sigma_{W}^{2}\right]; \end{array}$$ (14)

$$\begin{array}{c} M_{2}:\;\;\text{Pr}\left(D|\left\{\mu_{t}\right\},\left\{\sigma_{t}^{2}\right\}\right)=\prod_{t=1}^{T}\prod_{i=1}^{N_{t}}{\left(2\pi \sigma_{t}^{2}\right)}^{-1/2}\text{exp}\left[-{\left(y_{it}-\mu_{t}\right)}^{2}/2\sigma_{t}^{2}\right]. \end{array}$$ (15)

The maximum likelihood parameters and the corresponding model scores are readily calculated (see S1 Methods).

Model selection based on normal distributions can be sensitive to noise in the data, particularly when group sizes are small. Bootstrap replicates were used to increase the robustness of estimates from small populations [26]. A bootstrap sample was obtained by sampling tumors uniformly with replacement. This bootstrap sample was then used as the input for model selection, and the posterior probability of each model was recorded. This procedure was repeated to generate 10,000 bootstrap replicates, which provided converged estimates.

An additional procedure was used to guard against the difficulty of estimating the within-tumor variance for tumors with small numbers of measured organoids. We performed model selection again, but this time restricted the tumors to those having at least two organoids. A new series of 10,000 bootstrap replicates was generated to obtain converged estimates for this sample. The same procedure was then performed requiring at least 3 organoids, at least 4 organoids, and so on up to at least 10 organoids.

### Variance components model

Variance components models describe the distributions of random variables in structured population where subgroups have shifted means but share a common variance. These models also provide a framework for hypothesis testing and provide unbiased estimates of variances. In standard usage, populations refer to individuals, and the structure arises from families within the population. Here, the population refers to individual organoids, and the structure arises because subsets of organoids correspond to a single tumor. After model selection as described above, the observation of organoid *i* from tumor *t* is denoted *y*_*ti*_. The variance components model considers nested hypotheses for *y*_*ti*_, expressed in terms of hypotheses *H*_0_ and *H*_1_ equivalent to models *M*_0_ and *M*_1_ above:

$$\begin{array}{c} H_{0}\;:\;y_{ti}=\mu_{0}+\epsilon_{ti},\;\;\epsilon_{ti}∼\text{Norm}\left(0,\sigma_{0}^{2}\right); \end{array}$$ (16)

$$\begin{array}{c} H_{1}\;:\;y_{ti}=\mu_{t}+\epsilon_{ti},\;\;\epsilon_{ti}∼\text{Norm}\left(0,\sigma_{W}^{2}\right). \end{array}$$ (17)

Calculation of unbiased estimates for the null variance ${\hat{\sigma }}_{0}^{2}$, the within-tumor variance ${\hat{\sigma }}_{W}^{2}$, and the modeled variance ${\hat{\sigma }}_{M}^{2}$, are standard (see S1 Methods).

The ANOVA test statistic, *Q*_1_, is

$$\begin{array}{c} Q_{1}={\hat{\sigma }}_{M}^{2}/{\hat{\sigma }}_{W}^{2}. \end{array}$$ (18)

Under the null hypothesis of equal means, *μ*_1_ = *μ*_2_ = … = *μ*_*T*_, *Q*_1_ is a random variable following an *F*-distribution,

$$\begin{array}{c} Q_{1}∼F_{T-1,N-K}. \end{array}$$ (19)

If the null hypothesis is rejected, an unbiased estimate for the between-tumor heterogeneity is ${\hat{\sigma }}_{B}^{2}$, with ${\hat{\sigma }}_{B}^{2}+{\hat{\sigma }}_{W}^{2}={\hat{\sigma }}_{0}^{2}$.

### Power analysis

The phenotypes *y*_*ti*_ permit discovery of associations with biological or experiment factors, including gene expression levels, genetic variants, or culture conditions. These biological factors are denoted *x*_*ti*_ for factor *x* measured in organoid *i* from tumor *t*. The tumor mean *μ*_*t*_ is incorporated as a random effect and the associated factor as a fixed effect, leading to a mixed effect model:

$$\begin{array}{c} y_{ti}=\mu_{t}+\beta x_{ti}+\epsilon_{ti},\;\epsilon_{ti}∼\text{Norm}\left(0,\sigma_{\epsilon }^{2}\right). \end{array}$$ (20)

Separate tests can be conducted for tumor means, generally denoted $\bar{x}$ and $\bar{y}$, and within-tumor deviations, generally denoted *δx* and *δy*:

$$\begin{array}{ccc} {\bar{y}}_{t} & = & \mu_{t}+\beta_{B}{\bar{x}}_{t}+{\bar{\epsilon }}_{t},\;{\bar{\epsilon }}_{t}∼\text{Norm}\left[0,N_{t}^{-1}\sigma_{\epsilon }^{2}\right]; \end{array}$$ (21)

$$\begin{array}{ccc} \delta y_{ti} & = & \beta_{W}\delta x_{ti}+\delta \epsilon_{ti},\;\delta \epsilon_{ti}∼\text{Norm}\left[0,\left(1-N_{t}^{-1}\right)\sigma_{\epsilon }^{2}\right]. \end{array}$$ (22)

Here we have represented the parameter *β* from the mixed effects model as two separate parameters, *β*_*B*_ for the between-tumor test and *β*_*W*_ for the within-tumor tests.

The relationship between the type I and II error rates, the fraction of variance explained $R_{B}^{2}$, and the population size (number of tumors *T*) is

$$\begin{array}{c} Q_{B}^{2}=\left(T-1\right)R_{B}^{2}/\left(1-R_{B}^{2}\right)={\left(z_{I}-z_{II}\right)}^{2}, \end{array}$$ (23)

where $Q_{B}^{2}$ is the non-centrality parameter, *z*_*I*_ is the normal quantile corresponding to the desired false-positive rate, and *z*_*II*_ is the normal quantile corresponding to the desired false-negative rate (see S1 Methods). Similarly, for the within-tumor test,

$$\begin{array}{c} Q_{W}^{2}=\left(N-T-1\right)R_{W}^{2}/\left(1-R_{W}^{2}\right)={\left(z_{I}-z_{II}\right)}^{2}. \end{array}$$ (24)

The within-tumor test and the between-tumor test are typically controlled at the same significance threshold, for example *p* < 0.05/(number of genes tested). The non-centrality parameters $Q_{B}^{2}$ and $Q_{W}^{2}$ are usually different, however, which implies that the quantiles *z*_*II*_ and hence the power are usually different.

### Pooled designs

While measuring the invasiveness of each organoid is feasible, performing genomic analysis of each organoid could be prohibitively expensive. An alternative approach is to pool the most invasive and least invasive organoids, and then to perform genomic analysis of the pooled upper and lower tails. We have shown that power is optimized by selecting the upper and lower 27%, with efficiency equivalent to individual measurements of a population 80% as large; the selection threshold and efficiency are robust to sibship size, effect size, and allele frequency in the context of genetic studies [27].

Assuming pooled tests with *f*_*P*_ representing pooling efficiency, the relationship between power and variance explained is

$$\begin{array}{c} Q_{W}^{2}=f_{P}\left(N-T-1\right)R_{W}^{2}/\left(1-R_{W}^{2}\right)={\left(z_{I}-z_{II}\right)}^{2} \end{array}$$ (25)

for within-tumor tests.

We use these power relationships to calculate the critical effect size required to achieve specified Type I and Type II error given an experimental design:

$$\begin{array}{ccc} R_{B}^{2} & = & \left[1+\left(\sigma_{W}^{2}/N_{t}\sigma_{B}^{2}\right)\right]\frac{{\left(z_{I}-z_{II}\right)}^{2}/\left(T-1\right)}{1+{\left(z_{I}-z_{II}\right)}^{2}/\left(T-1\right)}, \end{array}$$ (26)

$$\begin{array}{ccc} R_{W}^{2} & = & \frac{{\left(z_{I}-z_{II}\right)}^{2}/f_{P}\left(N-T-1\right)}{1+{\left(z_{I}-z_{II}\right)}^{2}/f_{P}\left(N-T-1\right)}. \end{array}$$ (27)

Note that although in principle the true values of the regression coefficients *β*_*B*_ and *β*_*W*_ should be identical, the true correlations *R*_*B*_ and *R*_*W*_ may be very different. Between-tumor variation in both the invasiveness {*y*_*ti*_} and the features {*x*_*ti*_} often weaken the between-tumor correlation, yielding *R*_*B*_ < *R*_*W*_. Furthermore, with 100-1000 organoids generated per tumor, *N* ≫ *T*, giving substantially more power to the within-tumor test.

### Correlation with K14 and organoid size

Linear models were used to perform between-tumor and within-tumor tests for correlation of invasion with protein expression of K14. For these statistical tests, we used tumors with at least 5 organoids, restricting analysis to 47 tumors and 811 organoids. Defining *y*_*ti*_ as before as the log_10_-transformed spectral power for organoid *i* from tumor *t*, we defined *x*_*ti*_ in turn as the rank-transformed total K14 and mean K14 of each organoid. Tumor means ${\bar{y}}_{t}$ and ${\bar{x}}_{t}$ and organoid deviations *δy*_*ti*_ and *δx*_*ti*_ were calculated. The between-tumor test used a linear model with an intercept, while the within-tumor test used a linear model without an intercept. We performed similar tests for rank-transformed organoid area.

For analysis restricted to extreme tails, the organoids with greatest spectral power (upper tail) and least spectral power (lower tail) corresponding to fraction *f* for each tumor were identified by multiplying the number of organoids for that tumor by *f* and then rounding fractional numbers up to the closest integer, ensuring at least one organoid in the upper and lower tail. For example, with a pooling fraction of 0.1, tumors with 5 through 10 organoids would have 1 organoid in the upper tail and 1 in the lower tail, and tumors with 11 through 20 organoids would have 2 in each tail. A 50% pooling fraction corresponds to analysis of all organoids; for an odd number of organoids in this case, the median organoid was assigned to the lower pool. Linear models were then re-calculated for this subset of organoids as an extreme-tails version of the within-tumor test. These models included an intercept term. A pooled test was then performed by calculating tumor-by-tumor mean values of *δx*_*ti*_ for organoids within the upper and lower tails. These were entered into a paired *t*-test for the paired upper and lower pool means for each tumor. Note that because a paired test was performed, results with pooled values of *x*_*ti*_ rather than *δx*_*ti*_ would give identical results.

## Results

### Invasiveness heterogeneity

**Fig 1:**
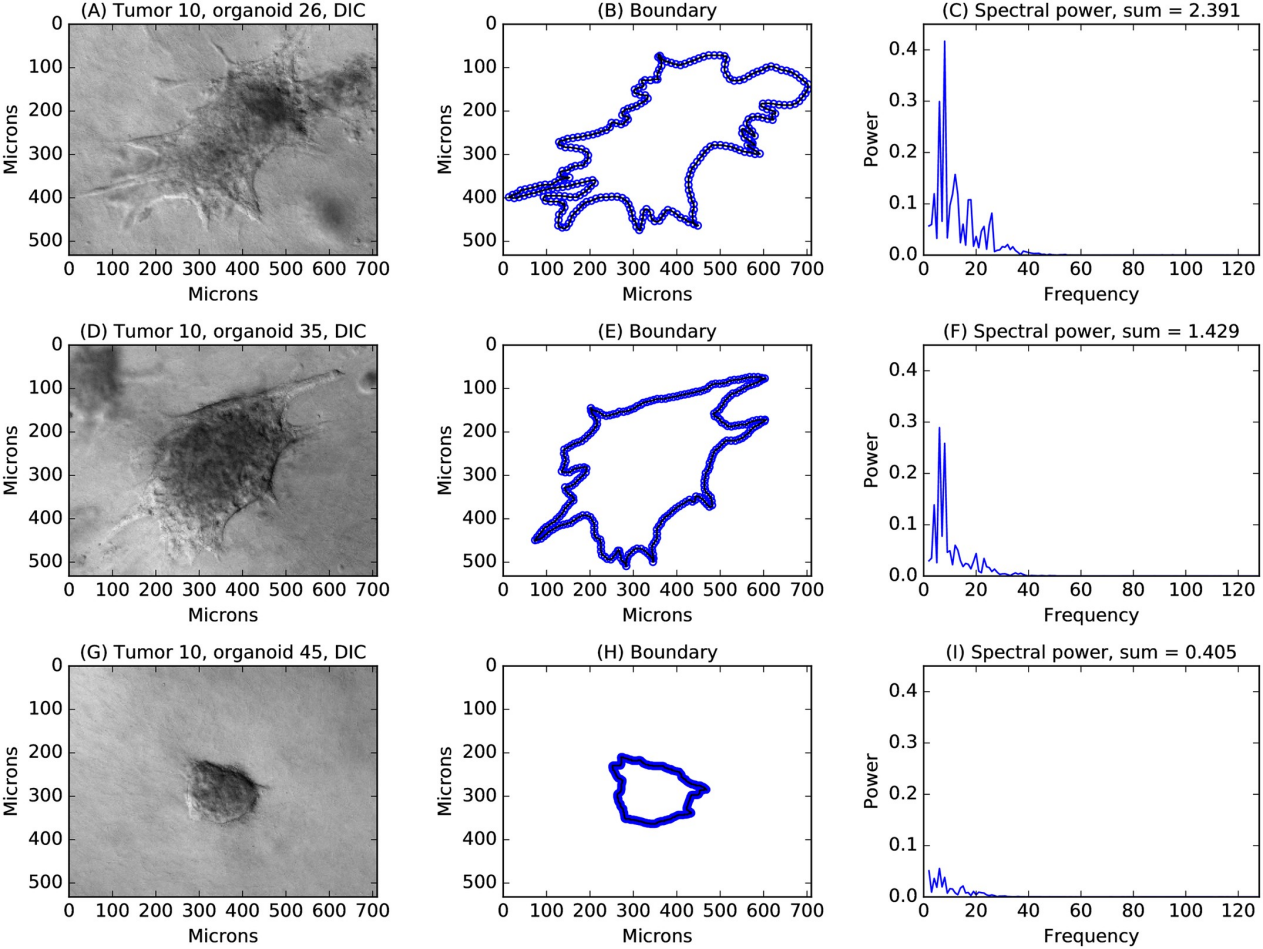
Defining a quantitative phenotype for invasion. The method used to define a quantitative phenotype for invasion is outlined for an organoid that is highly invasive (panels A, B, C), moderately invasive (panels D,E,F), and weakly invasive (panels G,H,I). These three organoids were selected from the 43 organoids generated from tumor 10, illustrating heterogeneity within a single tumor sample. Differential interference contrast (DIC) microscopy was used for image acquisition, with a scale of approximately 0.5 *μ*m per pixel and a field of view of approximately 530×710 *μ*m (panels A,D,G). Boundaries were segmented manually from DIC images and interpolated to 256 equally spaced points, sufficiently dense to track even the most invasive boundaries (panels B,E,H). A discrete Fourier transform was then applied separately to the *x* and *y* components of the discrete points, and the magnitudes of the corresponding Fourier amplitudes were squared and added to obtain the raw spectral power. Fourier mode 0, which represents the centroid of the boundary, was set to 0. The remaining modes were normalized by the power of Fourier mode 1 to provide scale invariance. Filters were applied to smooth effects from discrete pixel size and to emphasize the contributions of higher frequency modes, yielding a smoothed and weighted power spectrum for each organoid (panels C,F,I). The sum of the area under the spectrum, termed the spectral power, provides a single quantitative measure of invasiveness.

Tissue samples from breast cancer tumors were acquired from the Cooperative Human Tissue Network. Organoids were generated from 52 specimens, each from a different individual. Organoids were cultured in a three-dimensional collagen I matrix that, in previous work, has been shown to promote invasion [22], with each gel containing organoids from a single tumor. Organoids were imaged at day 6 using differential interference contrast (DIC) microscopy to identify organoid boundaries in the imaging plane. Boundaries were segmented manually using ImageJ (Fig 1).

**Fig 2:**
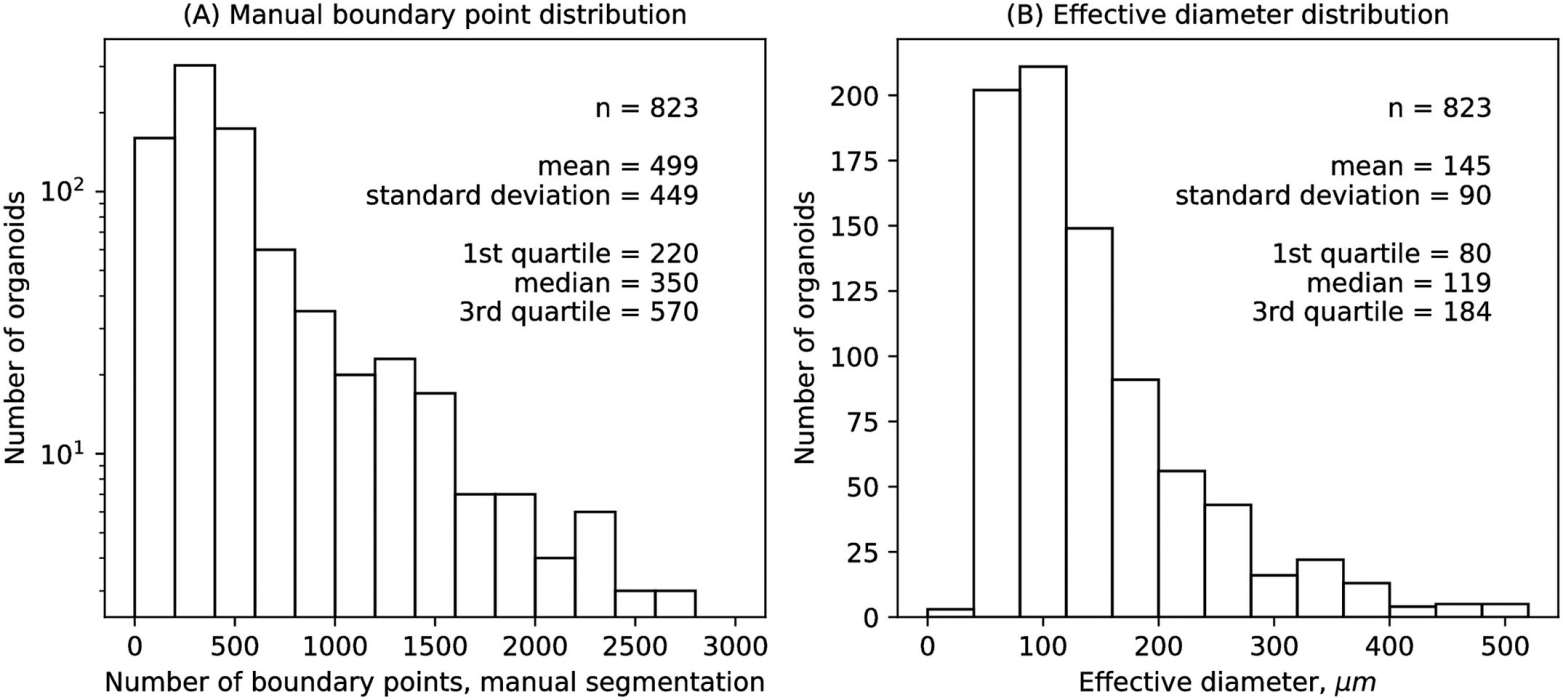
Organoid data distributions. (A) Distribution of the number of manual segmentation boundary points per organoid. (B) Distribution of organoid effective diameter, defined as $2\times \sqrt{\text{area}/\pi }$.

The number of boundary points from manual segmentation was variable (Fig 2A). For use with Fourier transforms, the boundary points were interpolated to 256 points with equal contour length between adjacent points. Interpolated boundary points were used to calculate the organoid area and effective diameter, defined as $2\sqrt{\text{area}/\pi }$. The organoid sizes were also variable (Fig 2B). The *x* and *y* coordinates were then Fourier transformed to yield spectral power over the entire bandwidth. Mode 0, corresponding to the boundary centroid, was set to 0, and the remaining modes were normalized by the power of mode 1, corresponding to normalizing the size to a unit circle. Filters corresponding to a spatial domain average, removing pixelation and jitter artifacts, and to a spatial domain derivative, corresponding to enhancing the detection of changes in curvature, were applied in the spectral domain. The resulting weighted and filtered power was summed for modes with magnitude 2 and higher to yield the spectral power, denoted *w*_*ti*_ for organoid *i* from tumor *t* (Eq 4).

This process is illustrated for 3 of the 43 organoids generated from tumor sample 10 (Fig 1). These three organoids qualitatively appear to be highly invasive, moderately invasive, and weakly invasive. The boundary of each organoid is depicted showing the linear interpolation points. The density of interpolation is clearly sufficient to capture the details of the boundary, even for highly invasive organoids, and results were robust to halving the number of interpolation points from 256 to 128 (see S1 File). Power spectra are plotted using the same *y*-axis scale to highlight the increase of spectral power with visually observed invasiveness. The area under the spectrum, termed the spectral power, is the single quantitative measure used here to characterize invasion. This continuous measure avoids the need for arbitrary binning with subjective criteria. It instead permits a reproducible assessment that could be automated for high-throughput characterization.

The individual spectral components provide invasive behavior fingerprints. Lower-order components generally reflect overall lengthening of an organoid along one or more axes, and higher-order components arise from more convoluted boundaries. While spectral components are not considered individually here, they could be used to cluster similar invasive patterns that may correspond to distinct invasion mechanisms.

**Fig 3:**
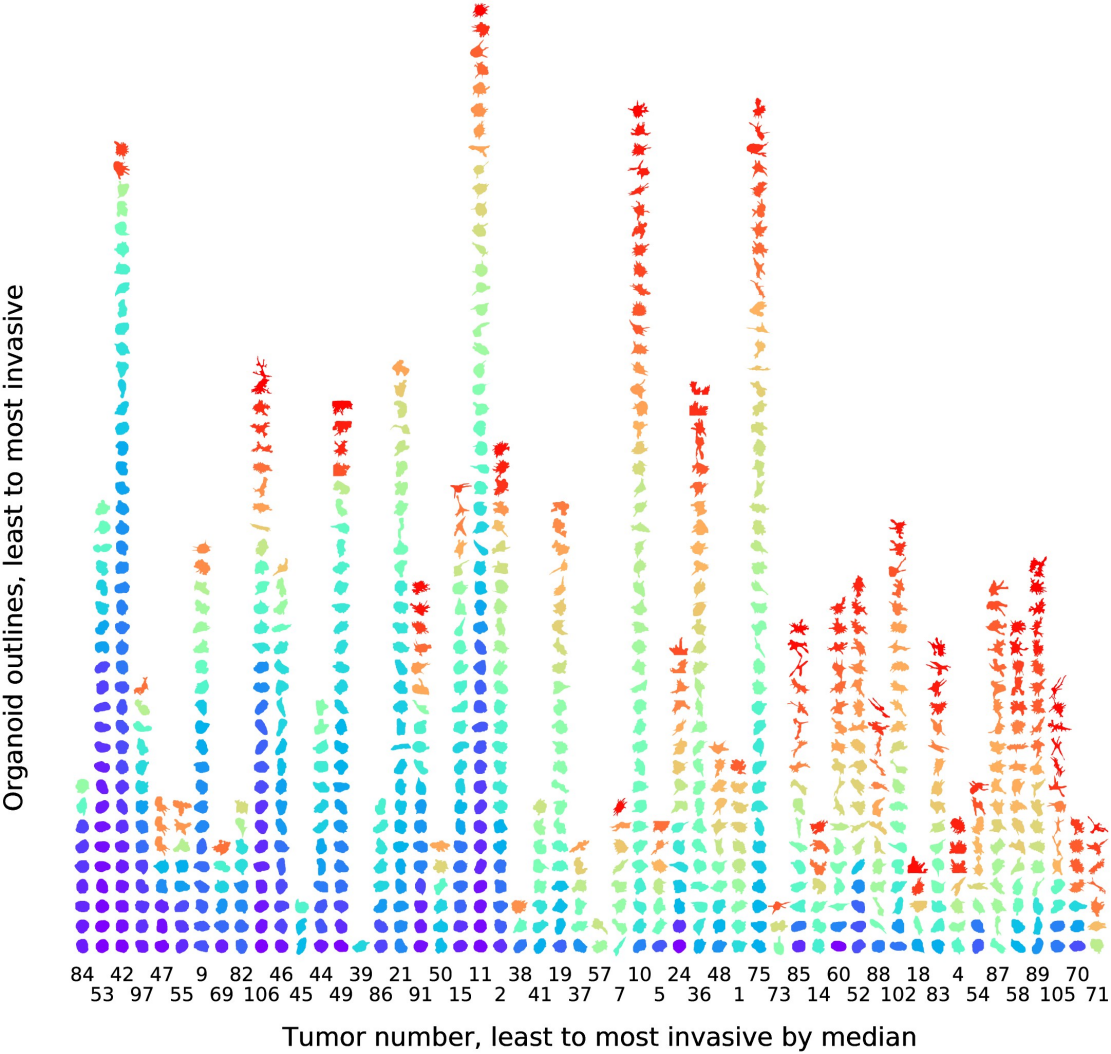
Invasiveness is heterogeneous between and within tumors. Organoid boundaries are shown for 823 organoids generated from 52 breast tumors and imaged after six days of growth in 3D culture. Each column corresponds to organoids from a single tumor, denoted by an identifier underneath the column. Organoid boundaries were converted to a quantitative spectral power phenotype, represented by a false color map from blue (non-invasive) to red (highly invasive). For each tumor, organoids are stacked from less invasive to more invasive as characterized by the spectral power. Tumors are then arranged from left to right based on the median organoid invasiveness. Differences in numbers of organoids per tumor are from constraints on experimental capacity rather than biological differences between tumors. Heterogeneity is observed on both the horizontal axis (between-tumor variation) and the vertical axis (within-tumor variation).

Organoids for all 52 tumor samples were characterized using this spectral method, with a resulting ordering that generally agrees with visual impressions of invasiveness (Fig 3). Each column corresponds to organoids generated from a single tumor. Within each column, organoids are stacked from less invasive to more invasive. Tumors are ordered left to right by the median organoid invasiveness. The number of organoids per tumor varies due to experimental constraints, not due to any inherent difficulty in generating or characterizing organoids from specific tumors. Invasiveness is heterogeneous both between tumors and within tumors. Between-tumor heterogeneity is apparent along the horizontal axis, and within-tumor heterogeneity is similarly apparent along the vertical axis.

These data indicate that tumors differ systematically in their ability to generate invasive organoids. Similarly, organoids generated from different cells within a tumor show different abilities to invade. Finally, the spectral power method is robust even for highly invasive organoids whose boundaries are partially truncated by the field of view, for example the top boundaries of the two most invasive organoids from Tumor 49 (Fig 3, 14^th^ from the left). The spectral power nevertheless properly characterizes these organoids as invasive.

### Statistical model selection

**Fig 4:**
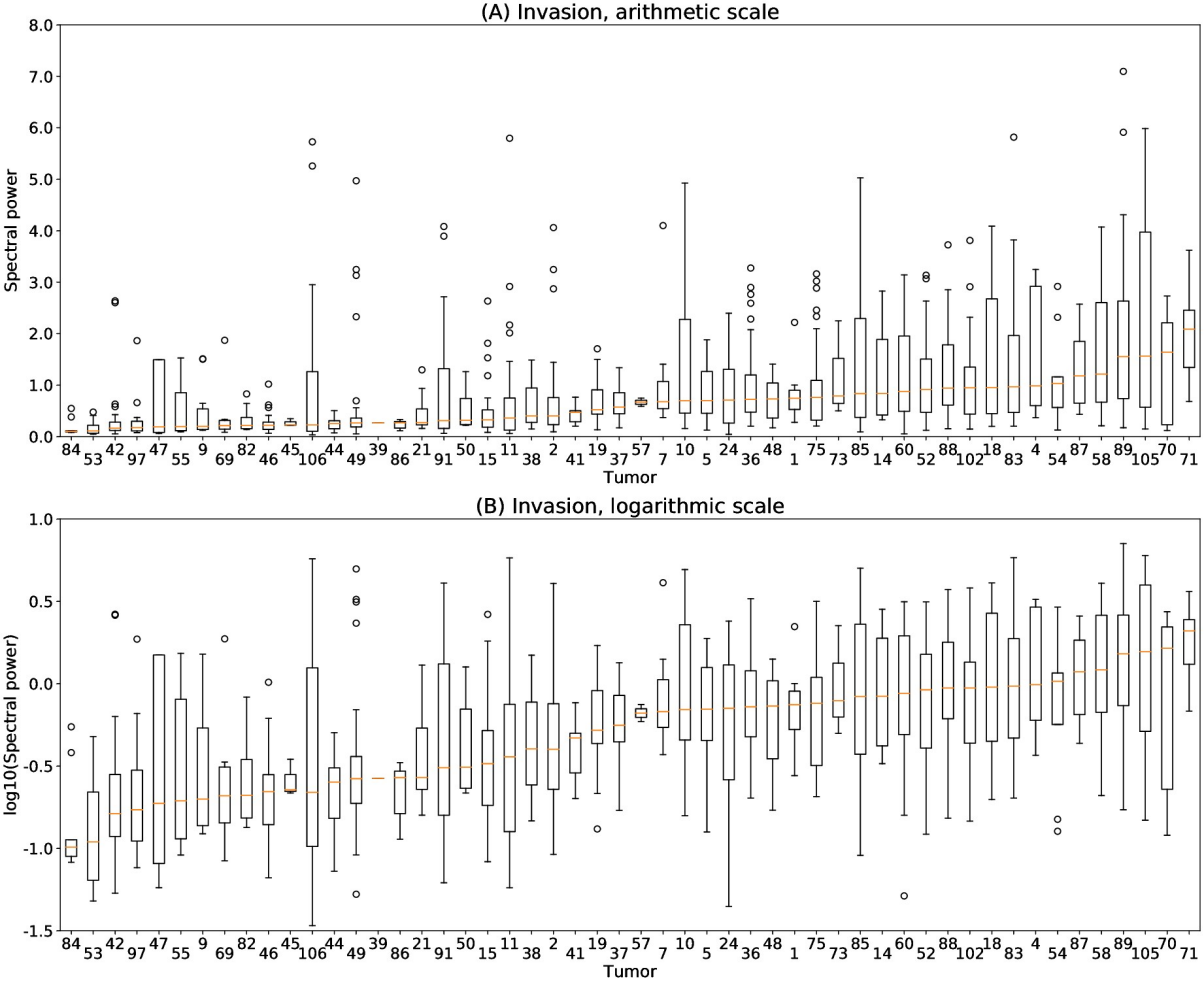
Distribution of quantitative invasion phenotypes. Each boxplot represents the distribution of invasion scores for organoids generated from a single tumor, with tumors ordered from left to right by median organoid invasiveness. The boxplot for each tumor indicates the median value (red bar), lower and upper quartile values (box extent), and outliers as individual points. (A) Distributions generated using invasion on an arithmetic scale are asymmetric, with the median closer to the first quartile and a larger upper tail. The interquartile range increases substantially with the median invasiveness. (B) Distributions generated using invasion on a logarithmic scale are more symmetric, with the median approximately halfway between the first and third quartile. The interquartile range increases less with the median.

Bayesian model selection was used to guide the choice of scale, arithmetic versus logarithmic for the invasion spectral power, and the choice of statistical model describing within-tumor and between-tumor heterogeneity. Invasion heterogeneity has qualitatively different appearance on an arithmetic versus logarithmic scale (Fig 4). On an arithmetic scale, distributions of invasiveness for each tumor are asymmetric, with the median usually closer to the first quartile and a larger upper tail (Fig 4A). In contrast, the distributions on a logarithmic scale are much more symmetric about the median (Fig 4B). A second difference is the dependence of the interquartile range on the median value. On an arithmetic scale, as the median invasiveness increases, the range increases as well. On a logarithmic scale, however, the interquartile range appears more constant. The logarithmic scale provides a phenotype that is closer to the assumptions of normal statistics because it has less skew, less heteroscedasticity, and permits negative values.

We used Bayesian statistics to provide a quantitative analysis of suitability of the arithmetic versus logarithmic scale for statistical modeling. For each scale, we considered three generative models for invasiveness within and between tumors, giving each model an equal prior of 1/3 (Eqs 13–15). Model 0, the null model, assumes equal mean and variance for each tumor. Model 1, the first alternative, introduces a different mean invasiveness for each tumor, but retains a shared variance across all tumors. Model 2, the second alternative, also permits different means, and further adds additional parameters to permit a separate variance for each tumor. Model 1 is a good description for traits that result from the combined effect of many genetic factors, each with a small contribution; by the Central Limit Theorem, this can lead to equal variance for different groups. Model 1 has the additional benefits of being the standard model for most work in statistical genetics and in permitting pooled estimates of variance, which are more robust than group-specific estimates. We then used maximum likelihood parameter estimates together with Schwarz’s Bayesian information criterion [25] to calculate the posterior probability of each model (Eq 12).

For invasion on an arithmetic scale, Model 2 is selected, with less than 1 × 10^−50^ probability assigned to Model 0 or Model 1. The assignment of all probability to Model 2 is a quantitative reflection of the qualitative observations of distribution skew and increasing variance with increasing median invasiveness (Fig 4A).

**Fig 5:**
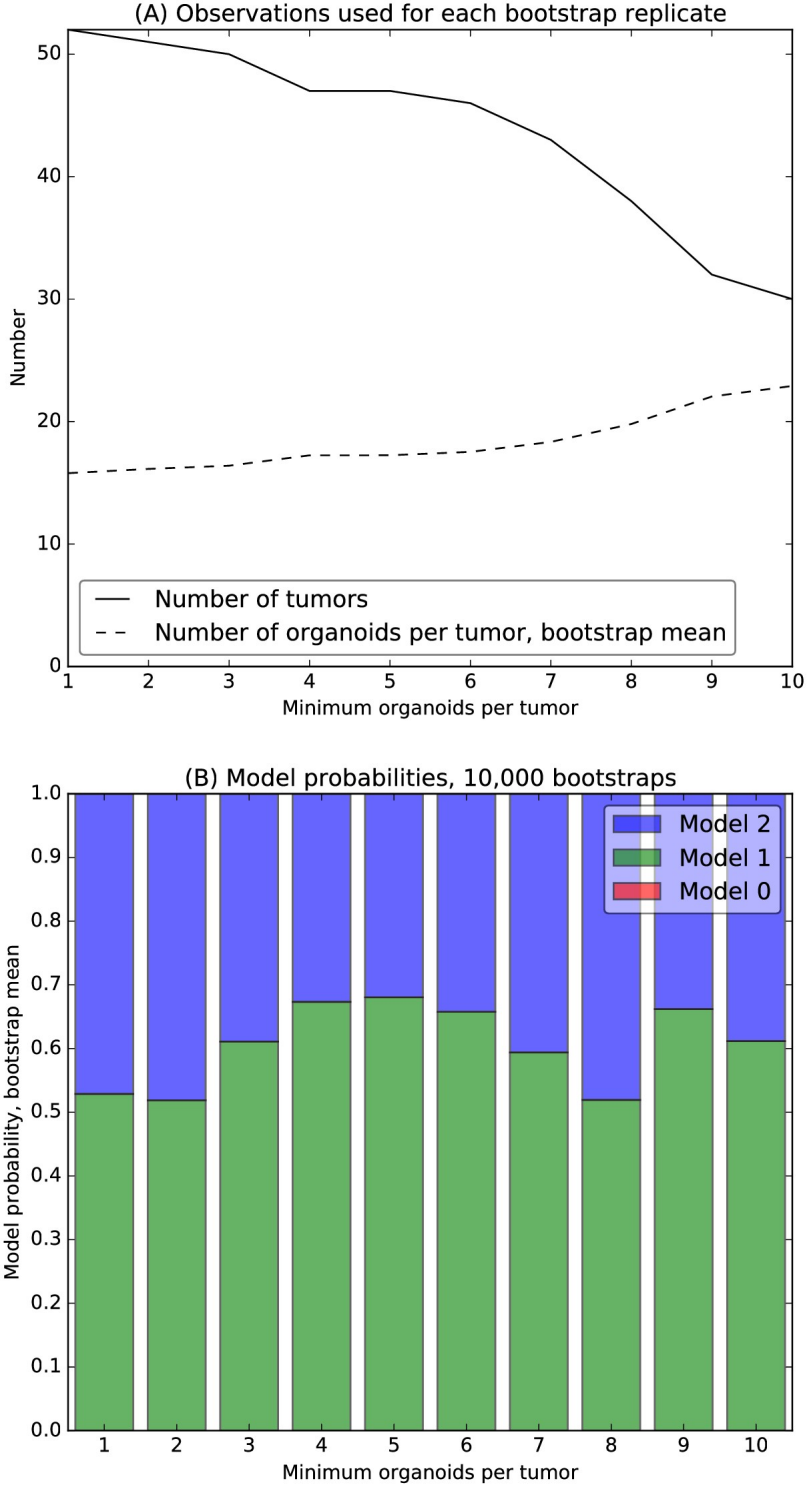
Bootstrapped Bayesian model selection. (A) Bootstrap replicates were used to increase robustness to limited number of tumors and organoids per tumor. Bootstraps were conducted for all 52 tumors and then, with increasing stringency, for tumors generating at least 2 through 10 organoids, with 30 tumors meeting the final requirement (solid line). The average number of organoids per tumor increased from 15.8 to 22.9 for these replicates (dashed line). (B) Three generative models were considered for between-tumor and within-tumor variation in logarithmic-scale organoid invasiveness: Model 0 assumes a single mean and variance shared by all tumors; Model 1 assumes a shared variance, but assigns each tumor its own mean; Model 2 assigns each tumor its own mean and variance. Converged estimates were obtained from 10,000 bootstrap replicates for thresholds of 1 organoid per tumor up to 10 organoids per tumor. For these thresholds, the posterior probability is 55-65% for Model 1 (green bars), with the remaining probability assigned to Model 2 (blue bars). Model 0 (red bars) had vanishing probability, not visible on this scale.

In contrast, for the logarithmic scale, Model 1 and Model 2 both have appreciable probability, indicating the suitability of statistical models that assume equal variance for organoid invasiveness within each tumor (Fig 5). To ensure that posterior probabilities for the logarithmic scale were robust to a variable number of organoids per tumor, we performed calculations that required up to 10 organoids per tumor. Of the 52 tumors, 30 met this requirement. We also performed calculations using all 52 tumors, and all thresholds in between (Fig 5).

We further increased the robustness of estimates for the logarithmic scale by using 10,000 bootstrap replicates. These calculations suggest a posterior probability of 55-65% for Model 1, the remaining probability assigned to Model 2, and vanishing probability for Model 0 (Fig 5B). The posterior probabilities vary for different values of the organoid-per-tumor threshold. This variation reflects statistical noise inherent in a small data set. It is an interesting open question whether a larger data set would definitively select the simpler Model 1 or the more complex Model 2. Systematic differences between tumors, for example differences in overall mutation rates, could lead to variation in the scale of within-tumor heterogeneity. Nevertheless, for the data at hand, our analysis indicates that a standard shared-variance model, Model 1, is adequate to describe the variation of log-scale invasiveness as a quantitative trait.

We conclude from this analysis that the log-transformed spectral power is compatible with a normal mixture model, in which each tumor has an individual mean and all tumors share a single variance. This model is the standard statistical framework for analyzing quantitative traits in population genetics and is described by conventional parametric statistics. The spectral measure on its original arithmetic scale is less suitable because of skew and heteroscedasticity. The log-transform in this context is similar to log-transforms used for gene expression and other quantitative characters that are better described by log-normal distributions than by normal distributions, possibly reflecting multiplicative rather than additive noise.

### Within-tumor variance can be larger than between-tumor variance

The previous results indicate that the standard framework for analyzing genetic and phenotypic variation in a structured population, a variance components model, is appropriate for analysis of variation of invasion for organoids generated from tumors. Each tumor is analogous to a family in a population-based study, and each organoid is analogous to a sibling within the family. For organoid *i* generated from tumor *t*, the log-scale invasiveness score *y*_*ti*_ is modeled as a tumor mean *μ*_*t*_ plus a deviation *ϵ*_*ti*_, with $\mu_{t}∼\text{Norm}\left(\mu_{0},\sigma_{B}^{2}\right)$ and $\epsilon_{ti}∼\text{Norm}\left(0,\sigma_{W}^{2}\right)$. The total variance for an organoid, denoted $\sigma_{0}^{2}$, is the sum of the between-tumor variance, $\sigma_{B}^{2}$, and the within-tumor variance, $\sigma_{W}^{2}$.

This structure is essentially identical to the structure of an ANOVA model testing the hypothesis that all *μ*_*t*_ values are identical; under the null hypothesis, the test statistic follows an *F*_*T*−1, *N*−*K*_ distribution for *T* tumors and *K* total organoids. Applying this test to the full organoid data (52 tumors and 823 organoids corresponding to *F*_51,771_, we find ANOVA test statistic *F* = 7.21 and *p*-value 8.8 × 10^−39^. The strong significance of this hypothesis test is the frequentist equivalent of the Bayesian model selection assigning vanishing probability to the null model.

The ANOVA model provides an estimate of the variance components from the between-tumor heterogeneity, $\sigma_{B}^{2}$, and the within-tumor heterogeneity, $\sigma_{W}^{2}$, to the overall variance, $\sigma_{0}^{2}$. The variance components model estimates that between-tumor heterogeneity is responsible for 28% of the total variance, and within-tumor heterogeneity is responsible for 72% of the total (Table 1). Thus, within-tumor invasion heterogeneity is 2.6× larger than between-tumor heterogeneity. It may be surprising that in these data, the within-tumor heterogeneity is larger than between-tumor heterogeneity. Nevertheless, large within-tumor heterogeneity is consistent with similar observations for heterogeneity within tumor specimens. Our prior experimental observations have shown that invasion is led by specialized cancer cells with a gene expression pattern termed “basal”, and that the abundance of basal-type cells within a tumor can range from 1–30% of cancer cells [10]. Inhomogeneous distributions of basal-type cells within a tumor could lead to organoids with different basal cell fractions and consequently different invasiveness. We also note that clinical considerations restrict tissue acquisition to relatively large tumors, usually larger than 1 cm in at least one dimension with a definitive clinical diagnosis from diagnostic biopsy, and greater variation may be present in the entire clinical population including smaller tumors.

**Table 1:** Variance components of invasion.

Component	Value	Fraction of toal
$\sigma_{0}^{2}$ (total)	0.2344	1.000
$\sigma_{B}^{2}$ (between-tumor)	0.0652	0.278
$\sigma_{W}^{2}$ (within-tumor)	0.7218	0.722

Variance components were calculated on a log_10_ scale from 823 organoids generated from 52 tumors.

### Expected power

In population genetics, heterogeneity within families often provides a powerful substrate for identifying genetic or genomic factors that drive phenotypes. Within-tumor heterogeneity is problematic in the clinical setting, as region-specific and cell-specific differences in resistance to chemotherapeutics makes it more challenging to eliminate the entire tumor with a given drug regimen [4]. However, in the context of an association study, heterogeneity can be exploited to identify the molecular determinants of the quantitative trait. By removing between-tumor sources of variation, within-tumor tests can increase the power to detect genetic, genomic, and epigenomic factors that drive invasion and, more generally, metastasis. Power analysis to identify the critical number of tumors and organoids to detect a biologically relevant effect is essential to assess the prospect of using organoids as part of a human population study.

We envision statistical tests that model the dependence of the invasiveness, denoted *y*, on biological factors considered one at a time, denoted *x*. These factors may represent expression levels of particular transcripts, germ-line or somatic genetic variants, epigenomic states, or other collective measures. Simplifying to a single organoid per tumor (formally equivalent to multiple organoids measured, but all giving identical results and thus not adding information), the statistical model for the invasiveness *y*_*t*_ of tumor *t* with biological factor *x*_*t*_ is

$$\begin{array}{c} y_{t}∼\text{Norm}\left[\mu_{0},\beta \left(x_{t}-x_{0}\right)\right], \end{array}$$ (28)

where *μ*_0_ is the population-level mean invasiveness and *x*_0_ is the population-level biological factor. The null hypothesis *β* = 0 and alternative hypothesis *β* ≠ 0. The fraction of variance explained by *x* under the alternative hypothesis is

$$\begin{array}{c} R^{2}\equiv \beta^{2}/\sqrt{\text{Var}(y)\text{Var}(x)}. \end{array}$$ (29)

The corresponding test statistic is

$$\begin{array}{c} Q^{2}\equiv {\hat{\beta }}^{2}/\sigma_{\beta }^{2}, \end{array}$$ (30)

where $\hat{\beta }$ is the maximum likelihood estimate of *β* and $\sigma_{\beta }^{2}$ is its estimated variance. Under the null hypothesis, $Q^{2}∼\chi_{1}^{2}$, a *χ*^2^ random variable with 1 degree of freedom. Under the alternative, *Q*^2^ follows a non-central *χ*^2^ distribution with non-centrality parameter (*T* − 1)*R*^2^/(1 − *R*^2^) for *T* total tumors observed.

A compact equation connects the test statistic *Q*^2^, the effect size *R*^2^, the two-tailed type I error *ϵ*_*I*_ with quantile *z*_*I*_ defined by Φ(*z*_*I*_) = 1 − *ϵ*_*I*_/2 for cumulative normal distribution Φ, and quantile *z*_*II*_ defined through the type II error *ϵ*_*II*_ as Φ(*z*_*II*_) = *ϵ*_*II*_ (Eqs 23 and 24):

$$\begin{array}{c} Q^{2}=\left(T-1\right)R^{2}/\left(1-R^{2}\right)={\left(z_{I}-z_{II}\right)}^{2}. \end{array}$$ (31)

For genome-wide significance with 20,000 gene-based tests, *ϵ*_*I*_ = 0.05/20, 000, and *z*_*I*_ = 4.708. For a typical 80% requested power, *ϵ*_*II*_ = 0.2, and *z*_*II*_ = −0.842. Under these assumptions, the population required to detect effect size *R*^2^ is *T* ≈ 30.8(1 − *R*^2^)/*R*^2^.

In a simple model for a phenotype that depends on *G* genes with each gene contributing equally, for example, the *R*^2^ for an individual gene would be 1/*G*. Mendelian disease genes often have *R*^2^ in the range 0.05 to 0.1, with mutant alleles in 10–20 different genes leading to the same syndrome through phenocopy. Variants identified from genome-wide association studies (GWAS) have *R*^2^ ∼ 0.01, or even smaller in large meta-analyses. Thus, factors that explain even 1% of the variation in tumor invasion could have high biological relevance in identifying pathways and potential targets. Corresponding population sizes required are 280 tumors required to detect a Mendelian-like association with *R*^2^ = 0.1 and ∼3000 tumors required to detect a GWAS-like association with *R*^2^ = 0.01.

These population sizes can be vastly reduced, however, by exploiting the within-tumor heterogeneity, ignored in the above analysis. Each observation of tumor invasiveness *y*_*ti*_ is separated into the population mean *μ*_0_, the tumor-based mean ${\bar{y}}_{t}$, and the deviation *δy*_*ti*_ for organoid *i* generated from tumor *t*. Similarly, biological factors *x*_*ti*_ are separated into the population mean *y*_0_, the tumor mean $\bar{x}$, and the deviation *δx*_*ti*_. In the framework of a variance components model, these lead to between-tumor and within-tumor tests that are statistically independent:

$$\begin{array}{ccc} {\bar{y}}_{t} & ∼ & \text{Norm}[\mu_{0},\beta_{B}\left({\bar{x}}_{t}-x_{0}\right)] \end{array}$$ (32)

$$\begin{array}{ccc} \delta y_{ti} & ∼ & \text{Norm}(\mu_{t},\beta_{W}\delta x_{ti}). \end{array}$$ (33)

The null hypothesis for the between-tumor test is *β*_*B*_ = 0, with two-tailed alternative *β*_*B*_ ≠ 0; similarly, the null for the within-tumor test is *β*_*W*_ = 0, with alternative *β*_*W*_ ≠ 0. Equations similar to Eq 31 can be derived for the between-tumor test, Eq 23, and the within-tumor test, Eq 24. In a typical scenario in which 100’s to 1000’s of organoids are generated per tumor, the within-tumor test can have excellent power. For a GWAS-type association with *R*^2^ = 0.01, the number of observations required remains 3000. These may be obtained from 100’s of organoids generated per tumor from only 10’s of tumors, rather than 1000’s of tumors required for a between-tumor test.

These power relationships assume that individual measurement of invasiveness *y* and biological factor *x* are available for each of *N* total organoids. This assumption is reasonable for invasiveness characterized through semi-automated microscopy, but may be impractically expensive for genomics characterization. An experimental design that vastly reduces cost while maintaining power in this scenario is extreme tails pooling [28], motivated and validated by extreme tails analysis in human genetics [29, 30]. Pooling increases the savings, even after accounting for technical sources of variation [27, 28, 31, 32].

In a pooled RNA-Seq study, the most invasive organoids would be pooled to generate a single RNA-Seq library, and similarly the least invasive organoids would be pooled to generate a second library. The number of RNA-Seq libraries for a within-tumor test would then be reduced from the number of organoids to twice the number of tumors, a 100–1000× reduction in effort. A general conclusion for an additive model is that pooling the upper 27% and the lower 27% optimizes the power and has 80% efficiency, defined as having equivalent power to an individual-level test conducted on 80% of the original population [27, 28].

**Fig 6:**
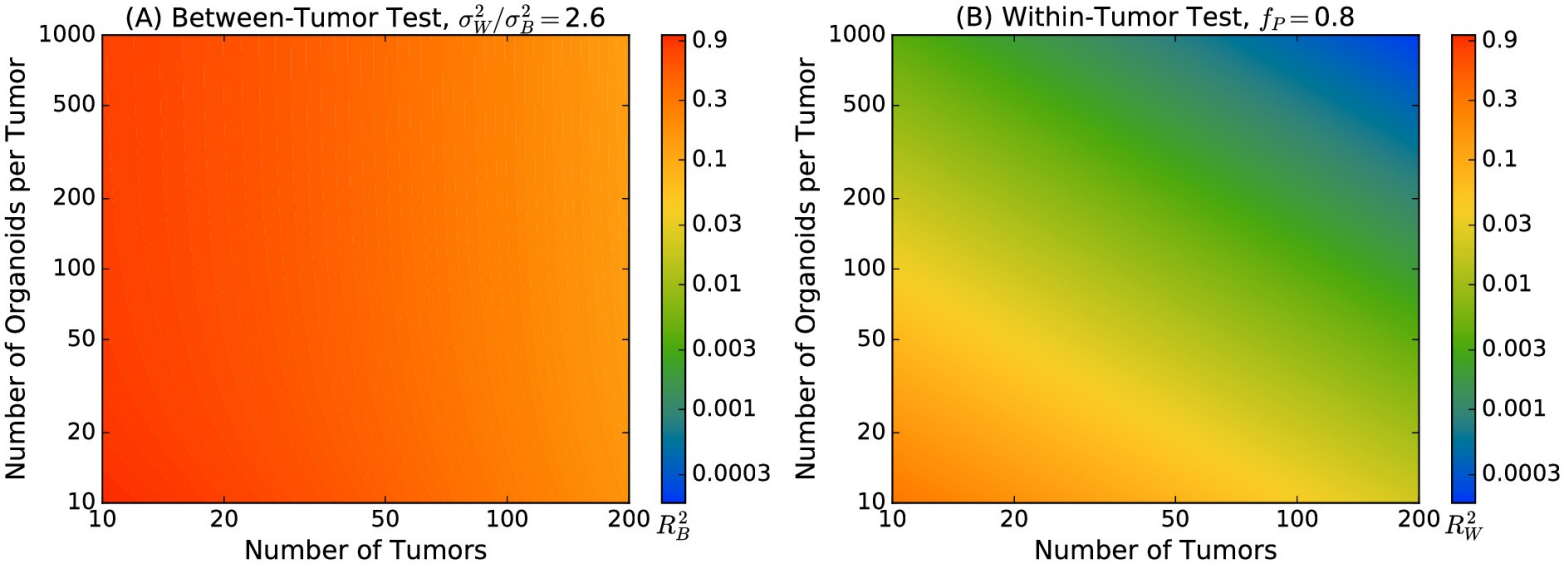
Power of a pooling-based design. The critical effect size defined as variance explained, *R*^2^, is shown for (A) between-tumor tests and (B) within-tumor tests. Calculations assume 20,000 two-tailed gene-based tests with genome-wide significance level 2.5 × 10^−6^ and 80% power. For between-tumor tests, the ratio of within-tumor to between-tumor variance is set to the observed value of 2.6. For within-tumor tests, pooling is assumed to reduce efficiency to 80%. Color bars indicate contour levels; between-tumor tests are limited to much larger effects and use only the upper region of the scale.

A pooling design slightly modifies the power relationships, primarily by increasing the number of organoids required by 10–20% relative to a design in which each organoid is individually characterized genomically (Eqs 26 and 27). This analysis indicates that studies of 10’s to 100’s of tumors could have excellent power to detect biological factors that are drivers or effectors of invasion (Fig 6). These figures illustrate the critical *R*^2^ values required to detect an effect at 80% power, assuming 20,000 gene-based tests with a corresponding two-tailed *p*-value of 2.5 × 10^−6^. For between-tumor tests (Fig 6A), the ratio of within-tumor to between-tumor variance is set to the observed value of 2.6. For within-tumor tests (Fig 6B), pooling is assumed to reduce efficiency to 80%. Contour values for the critical effect size use a common color scale.

While Fig 6A for between-tumor tests is has only slight color variation, Fig 6B for within-tumor tests uses the entire color scale. The limited region of the color scale observed for between-tumor tests is because they can only detect very strong effects, $R_{B}^{2}\geq 0.1$. The power, even after 200 tumors, permits only the observation of Mendelian-like driver or effector genes. In contrast, the power for within-tumor tests is far greater, with the ability to detect effects similar to those observed in GWAS, $R_{W}^{2}∼10^{-3}$ and below for 200 tumors. This improvement comes from exploiting the heterogeneity of organoids generated from a single tumor. Due to this heterogeneity, each organoid is similar to an independent observation, yielding a 100× to 1000× increase in the number of effective samples.

The contour lines for between-tumor tests are vertical (Fig 6A), whereas the the contour lines for within-tumor tests are at a 45° angle (Fig 6B). Between-tumor tests have vertical contours because 10–20 organoids are sufficient to define tumor-based means, and additional organoids provide little new information. In contrast, for the within-tumor test, additional organoids continue to improve power. Moving vertically therefore results in crossing contour lines corresponding to the ability to observe smaller and smaller effects. The number of observations for within-tumor tests is approximately the number of tumors times the number of organoids per tumor; on a contour plot with logarithmic scale, this corresponds to the observed 45° angle for contour lines. Thus, rather than recruiting more individuals to a study, generating many organoids from individual samples may be a more efficient route to increased study power.

### Associations with Keratin 14

Motivated by the predicted power of a population-based test, we used this approach to test the association of invasion with protein expression of Keratin 14 (K14). We chose protein characterization rather than RNA-Seq because these samples were not consented for genomics, and we chose K14 because of strong evidence linking K14 with laboratory analysis for tumor invasion in mouse and human [10] and clinical outcomes for breast cancer survival [12].

**Fig 7:**
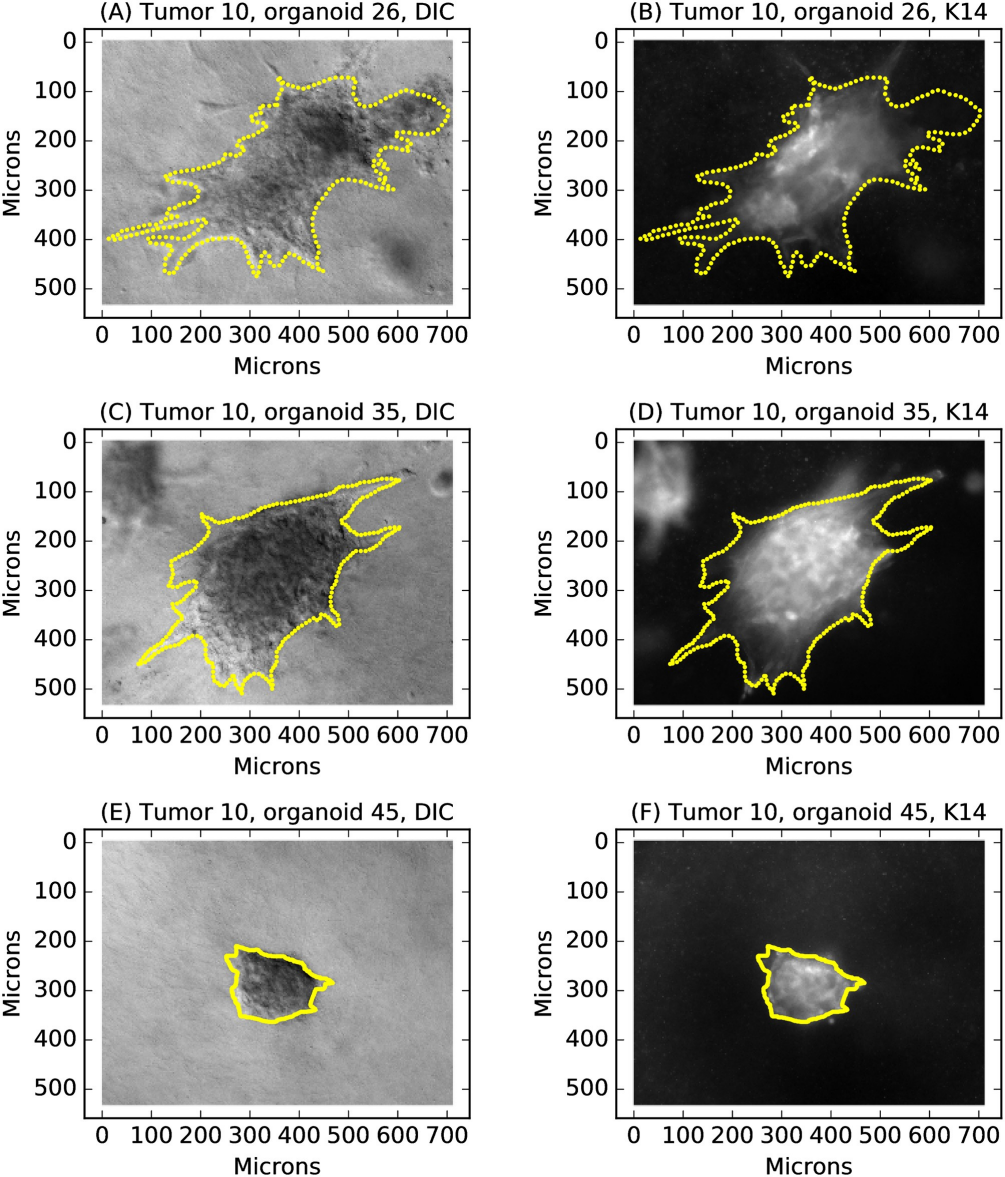
Defining a quantitative phenotype for invasion. The DIC images (panels A,C,E) were paired with K14 epifluorescence images obtained at identical resolution (panels B,D,F). Dots indicate boundaries from the DIC images interpolated to 256 equally spaced points and superimposed on both the DIC and K14 images.

**Fig 8:**
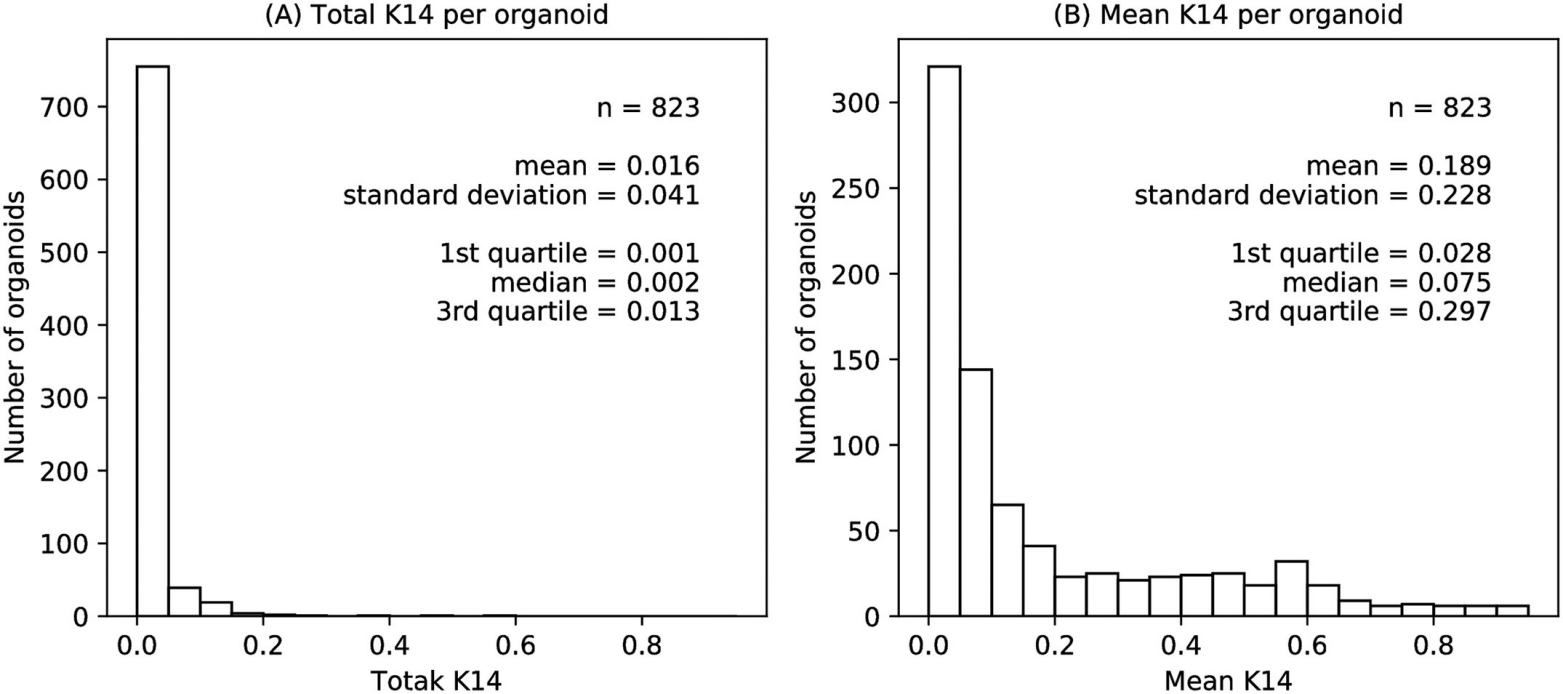
Organoid data distributions. (A) Histogram of total Keratin 14 (K14) expression per organoid, calculated as the sum of the K14 intensity on a [0, 1] scale for pixels within the organoid divided by the total number of image pixels. (B) Histogram of mean K14 expression, calculated as the sum of the K14 intensity divided by the area of the organoid in pixels. The organoid size is less than the image size, and therefore the mean K14 is greater than the total K14. Both the total and mean were rank-normalized to generate uniform distributions for robust statistical analysis.

We therefore quantified K14 by immunofluorescence using epifluorescence microscopy, with pixel values mapped from 0 (no expression) to 1 (saturation), for the same series of organoids imaged by DIC for invasion. Boundaries identified from DIC images were superimposed on the K14 images, and pixel intensities within each organoid boundary were gathered (Fig 7, the same organoids depicted in Fig 1)). The expression level for each organoid was quantified in two ways: summing over all pixels and then dividing by the total number of pixels in the image (“Total K14”) or by just the number of pixels inside the organoid boundary (“Mean K14”). The K14 distributions were right-skewed, with most organoids expressing low levels of K14 (Fig 8A and 8B). For robust analysis, total and mean K14 values were rank-normalized to obtain a uniform distribution.

Analyses were conducted according to the strategy outlined above: between-tumor tests for the tumor means estimated from the individual organoids, similar to a standard analysis of the tumor bulk, and within-tumor tests for invasiveness and K14 values corrected for the tumor mean. The within-tumor tests were conducted using three separate methods to explore the power of pooling. First, we performed a standard regression test that used each organoid as a single observation. Next, we restricted attention to the organoids in the tails of the distribution, and again performed a standard regression test using this subset of organoids. The tail fraction *f* ranged from symmetric tails of 5%, the most and least invasive 5% of organoids (fractional values rounded to the next integer) for each tumor, up to 50% corresponding to the entire data set, to investigate sensitivity to this parameter. Finally, we performed pooled tests by calculating the average value of either total K14 or mean K14 within each tail, then using the upper and lower pools in a paired-sample *t*-test with one set of paired observations for each tumor. This stepwise approach permits an understanding of loss of power from a reduced data set (all observations to extreme tails) versus loss of power from pooling the individual measurements into a single average value (extreme tails to pooled tests). Analyses were restricted to the 47 tumors generating at least 5 organoids, corresponding to 811 total organoids.

**Fig 9:**
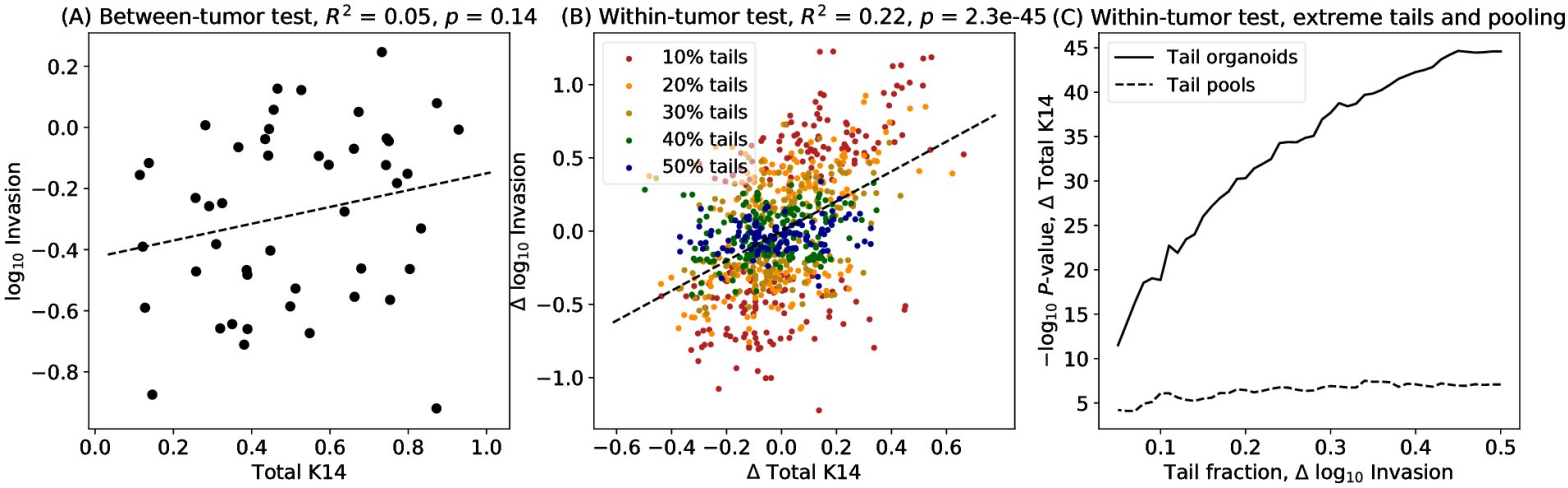
Association of invasiveness with total Keratin 14 protein expression. (A) Between-tumor tests of tumor means (points) do not yield significance for a linear model (dashed line). (B) The within-tumor test shows a highly significant association (*p* = 2.3 × 10^−45^) for a linear model (dashed line) between invasiveness and total Keratin 14 protein expression for individual organoids corrected for their tumor-specific baselines. Organoids in the extreme tails are shown for symmetric tails of 10% through 50%, with organoids in the 10% tail also belonging to larger tails and so on. The dashed regression line uses all the observations (50% tails). (C) Tests performed using organoids restricted to extreme tails are also highly significant (solid line). Pooled tests of mean values for organoids in the upper vs. lower tail, performed as a paired-sample *t*-test for each tumor, retain sufficient power for a gene-based test at 0.05 family-wise error rate, *p* < 2.5 × 10^−6^ when correcting for 20,000 genes or proteins tested, compatible for use with RNA-Seq (dashed line).

Between-tumor tests of total K14 versus invasion show a positive correlation, but are not significant, even at the single-test level (*p* = 0.14, Fig 9A). In contrast, within-tumor tests of total K14 were highly significant (*p* = 2 × 10^−45^, Fig 9B). Tests of organoids in the extreme tails retained high power, with *p* < 10^−10^ even for tail fractions as low as 5% (Fig 9C). Pooling reduced the power, but nevertheless retained sufficient power for *p* < 2.5 × 10^−6^, the typical threshold for a 0.05 family-wise error rate (FWER) when correcting for tests of 20,000 human genes or proteins.

Thus, the pooling approach described here should have power to detected a similarly sized effect from RNA-Seq data generated from pooled highly invasive versus non-invasive organoids. The statistical significance is weakly sensitive to pooling fraction, with similar results for pooling fractions from 20% to 50%. These results are in accord with theory developed for pooled analysis in the context of genome-wide association studies [27, 28, 32]. Two factors contribute to the lack of power of the between-tumor test relative to the within-tumor test. First, the number of observations is far smaller, the number of tumors that generated at least 5 organoids (47) versus the corresponding number of organoids (811). Second, variation in tumor-to-tumor baseline levels of invasiveness and K14 lead to an observed effect that is smaller for the between-tumor test, *R*^2^ = 0.05, than for the within-tumor test, *R*^2^ = 0.22.

**Fig 10:**
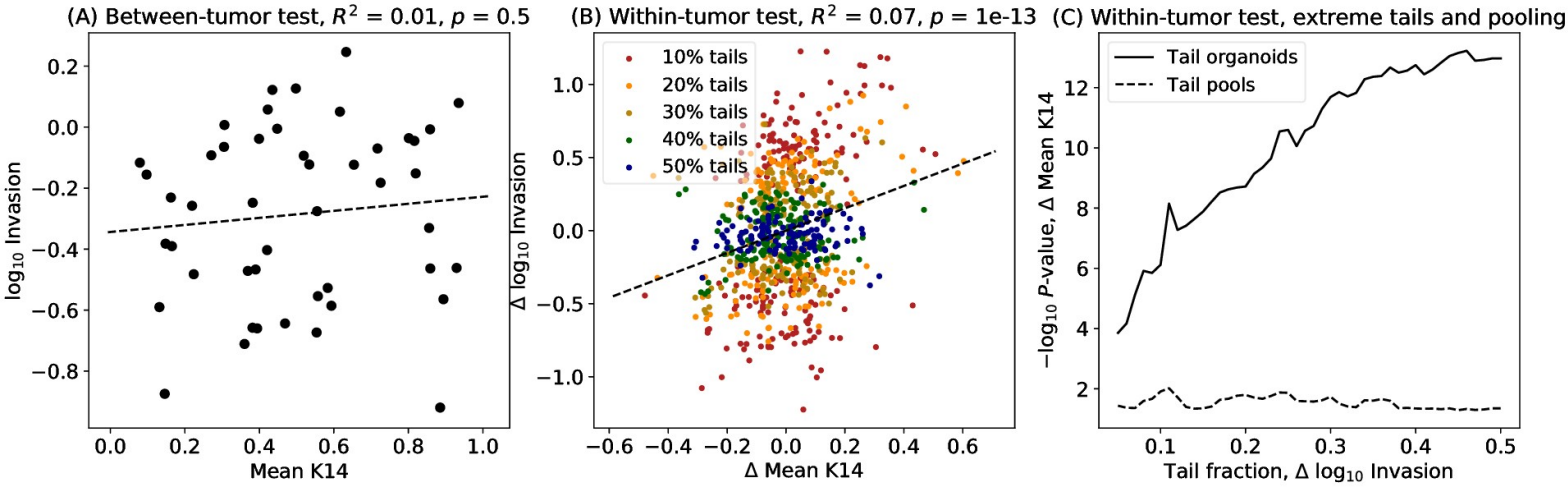
Association of invasiveness with mean organoid Keratin 14 protein expression. (A) Between-tumor tests of tumor means (points) do not yield significance for a linear model (dashed line). (B) The within-tumor test shows a highly significant association (1.0 × 10^−13^) for a linear model (dashed line) between invasiveness and mean Keratin 14 protein expression for individual organoids corrected for their tumor-specific baselines. Organoids in the extreme tails are shown for symmetric tails of 10% through 50%, with organoids in the 10% tail also belonging to larger tails and so on. The dashed regression line uses all the observations (50% tails). (C) Tests performed using organoids restricted to extreme tails are also highly significant (solid line). Pooled tests of mean values for organoids in the upper vs. lower tail, performed as a paired-sample *t*-test for each tumor, retain sufficient power for validation of individual findings (*p* < 0.05) but would not have sufficient power if corrected for multiple testing with gene-based (RNA-Seq) or proteome-wide tests.

We performed a similar series of tests for mean K14, correcting for possible confounding with tumor size (Fig 10). The within-tumor test remains highly significant (*p* = 1.0 × 10^−13^, Fig 10A), although less significant than the test of total K14. Tests of organoids in the extreme tails retain high power (Fig 10B). Pooled tests have sufficient power for this smaller effect for significance at the single-test level, *p* = 0.02 to 0.05 for a two-sided test with most pooling fractions, but not sufficient power for application to unbiased discovery from RNA-Seq. Between-tumor tests are not significant (*p* = 0.5), again highlighting the greater power of within-tumor tests.

**Fig 11:**
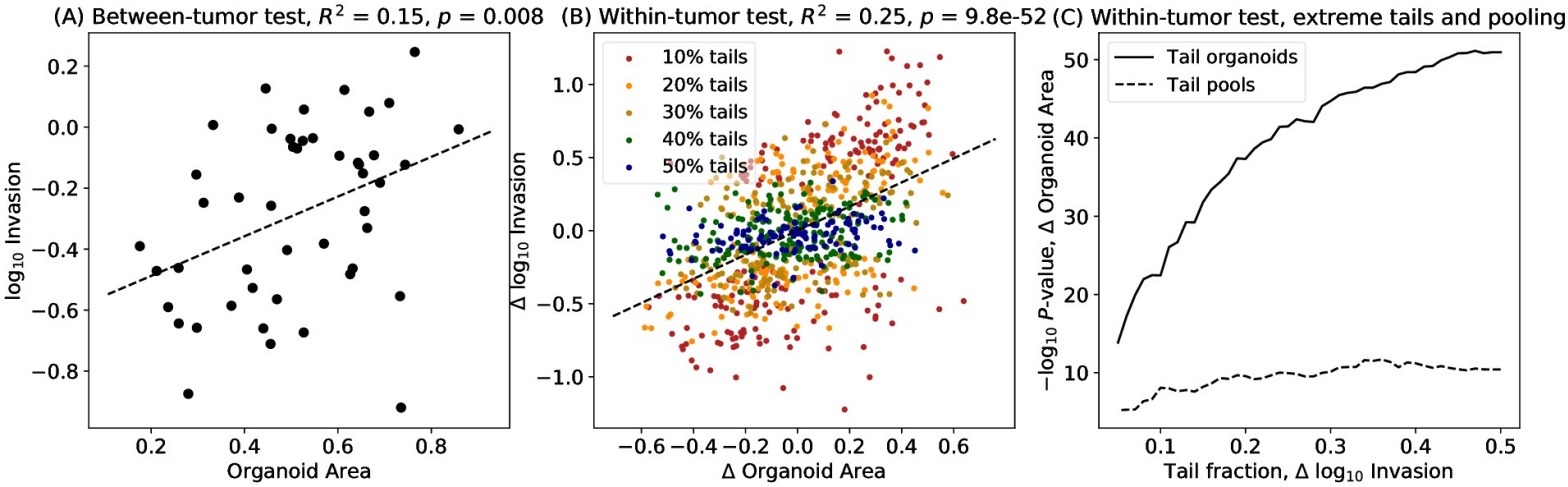
Association of invasiveness with organoid area. (A) Between-tumor tests between organoid size and invasiveness remain significant. (*p* = 0.008). (B) The within-tumor test shows a highly significant association (9.8 × 10^−52^) for a linear model (dashed blue line) between invasiveness and rank-transformed organoid area. Organoids are colored according to extreme tail membership. (C) Tests performed using organoids restricted to extreme tails are also highly significant (solid line), as are pooled tests for association of area with invasiveness.

Given the stronger association with total K14 than mean K14, we next investigated associations with organoid area, rank-transformed to a uniform distribution to permit robust analysis. The between-tumor test was significant at a single-test level (Fig 11A, *p* = 0.008). The within-tumor tests were highly significant (Fig 11B, *p* = 9.8 × 10^−52^), and extreme tail and pooled tests were also significant for genome-wide or proteome-wide tests (Fig 11C, *p* < 1 × 10^−10^ for many pooling fractions).

## Discussion

Population-based studies have been highly effective in revealing the genetic architecture of complex disease through genome-wide association studies (GWAS). Similar studies of somatic aberrations in cancer, whether genetic mutations or epigenetic or gene expression drivers, have not had the cohort sizes to permit similarly powered studies. Most tumor studies enroll fewer than 1000 individuals, whereas many GWAS have populations over 100,000. Our insight is that tumor heterogeneity, probed by organoids, permits 100’s to 1000’s of independent measurements from a single tumor, with tumors and organoids analogous to families and sibships in a population genetics study. Furthermore, similar to efficient genetic study designs using the most extreme or discordant siblings, we can increase efficiency be restricting analysis to organoids in the extreme tails of a phenotype distribution, or even single measurements of pooled tails. We have developed this approach successfully by validating the association of Keratin 14 with a quantitative phenotype for organoid invasion.

To permit quantitative analysis, we also have developed a new spectral-based phenotype for assessing the invasiveness of organoids generated from human breast tumors. This phenotype, when measured on a logarithmic scale, is ideal for quantitative trait analysis. Bayesian model selection indicates that a mixed effects model describes the data well: while each tumor has its own mean invasiveness, tumors share a common variance describing within-tumor heterogeneity. A variance components model finds that the within-tumor variance is approximately 2.6× larger than the between-tumor heterogeneity. The implication of this finding is that measurements of bulk tumor capture only a small fraction of the information inherent in heterogeneous tumor tissue. The ability to probe heterogeneity is the motivating factor for single-cell DNA and RNA sequencing. Here, we demonstrate that organoids are also able to probe this heterogeneity.

The organoid phenotype analyzed here is a surrogate for an initial step of metastasis, which in addition to invasion by individual cells or collectives also includes dissemination, re-seeding, and outgrowth. Organoids can provide invasion-related quantitative phenotypes as a step towards more comprehensive analysis of the genetic and genomic determinants of metastasis. In patients, tumor invasion can be assessed from two-dimensional sections by various methods as part of clinical prognosis [33]. Serial sections may be required to distinguish groups of cells that break off or bud from from the main organoid body from organoid extensions that enter and leave a single imaging plane [33, 34]. In this study, we have not observed images where organoid extensions may be mistaken for buds. Furthermore, such artifacts would be very unlikely to affect any conclusions: mistaking appendages for buds would result in an underestimate of invasion, but the organoids in question would already be ranked among the most invasive.

Molecular characterization of invasive versus non-invasive organoids could identify biological factors that are drivers and effectors of metastasis. These could provide new hypotheses for therapeutic targets and for predictive biomarkers. Again using the proven success of GWAS as a model, we analyze the power of between-tumor and within-tumor tests, analogous to between-family and within-family tests in population genetics. We find that between-tumor tests have limited power; even after 200 tumors have been analyzed, power is limited to detect effects similar to Mendelian genes in hereditary disorders. Within-tumor tests have excellent power, however, potentially equivalent to well-powered GWAS that can identify genes and variants that contribute as little as 1% to 0.1% to population-level variation. These results provide a possible explanation for the challenges in converting bulk tumor genomics data to therapeutically usefully knowledge: only the very largest effects have been detected because much of the information inherent in individual cells and sub-regions has been lost.

In addition to gene expression markers, direct observation of protein levels can be informative. Keratin 14 was quantified here using immunofluorescence. In previous work, we have used genetic engineering of fusion proteins for live imaging of fluorescent tags [7, 10, 11]. Direct tagging is most suited for work with genetically engineered mouse model systems. For human tumor tissue, antibody arrays may provide increased capabilities for proteomic profiling [35].

To validate the power of within-tumor tests, extreme tails analysis, and pooled tests, we demonstrated highly significant associations between increased expression and increased invasiveness, *p* = 2.3 × 10^−45^ for total Keratin 14 and *p* = 1.0 × 10^−13^ for Keratin 14 normalized to the imaged cross-sectional area. Tests restricted to organoids in the extreme tails retained high power and strong significance. Thus, generating 100’s of organoids per tumor and restricting analysis to the most invasive 5 to 10, selected either visually or by segmentation followed by automated analysis of the tumor boundary, could lead to new discoveries. Even greater experimental savings come with a pooled design, for example collecting the most extreme organoids from each tumor to generate a single RNA-Seq library for each tail. Pooled tests would have power to detect association for molecular features with effect sizes similar to total Keratin 14, even after correcting for multiple testing of 20,000 genes.

We conclude that organoid-based studies, enrolling on the scale of 100 participants with breast cancer and generating 100-1000 organoids per tumor, will have the ability to discover clinically relevant driver and effector genes for basic molecular drivers of phenotypes relevant for breast cancer. This population genetics framework is directly applicable to analyzing the molecular determinants in different cancer types and in future studies designed to correlate organoid phenotypes with clinical outcomes. Our approach could also be generalized to later stages of metastasis through development and validation of additional quantitative traits that capture biological variation in the capacity of cancer cells to, for example, disseminate or seed distant organs.

## Supporting information

S1 Methods Supporting methods. (PDF)

S1 Table Clinical cohort demographics. (XLSX)

S1 File Compressed archive including (i) free and open source software, released under a permissive BSD license, to perform spectral transforms and analysis (ibis2d.py); (ii) sample input data (due to size limitations, full data including boundary coordinates, DIC images, and K14 images for 823 organoids generated from 52 human breast tumors are available by contacting the authors); (iii) output tables with results for each organoid; (iv) implementation of within-tumor and between-tumor tests. (GZ)
