# Supplementary material for: Between-tumor and within-tumor heterogeneity in invasive potential
Source: PLoS Comput Biol. 2020 Jan 21;16(1):e1007464. doi: 10.1371/journal.pcbi.1007464 (PMC6994152; doi:10.1371/journal.pcbi.1007464)
Supplement: S1 Methods — (PDF) [file pcbi.1007464.s001.pdf]

Between-tumor and within-tumor heterogeneity in invasive potential
Supporting Methods

Veena Padmanaban, Yohannes Tsehay, Kevin J. Cheung, Andrew J. Ewald, Joel S. Bader

1 Spectral power

The total spectral power $\sum_{k=1}^{M/2} P_k$ is not invariant to scaling. Multiplying each (x_j, y_j) by a scale factor ρ yields a scale factor ρ^2 in each P_k. Experimental procedures were designed to yield approximately similar sized organoids. We therefore normalized the P_k terms to remove the scale factor, focusing on shape rather than scale. Normalization was performed by re-scaling each organoid to a unit circle. A unit circle centered at the origin with coordinates $(x_j, y_j) = (\cos[2\pi j/M], \sin[2\pi j/M])$ has Fourier components $\hat{x}_k$ that may be calculated explicitly,

$$\begin{aligned} \hat{x}_k &= \sum_{j=0}^{M-1} e^{-2\pi ijk/M} \cos(2\pi j/M) \\ &= (1/2) \sum_{j=0}^{M-1} e^{-2\pi ij(k-1)/M} + (1/2) \sum_{j=0}^{M-1} e^{-2\pi ij(k+1)/M}. \end{aligned} \tag{1}$$

These sums are of the form $S_n = \sum_{j=0}^{M-1} e^{-2\pi ijn/M}$ with $n = k-1$ and $n = k+1$. For n mod $M = 0$, $S_n = M$. For other values of n,

$$S_n = (1 - e^{-2\pi in})/(1 - e^{-2\pi in/M}) = 0. \tag{2}$$

Thus for the unit circle and $k \in \{0, \pm 1, \ldots, \pm M/2\}$, we find that $\hat{x}_k = M/2$ for $k = \pm 1$ and $\hat{x}_k = 0$ otherwise. Similarly, $\hat{y}_k = M/2$ for $k = \pm 1$ and 0 otherwise. Finally, for a circle of radius r centered at the origin, $P_k = r^2 M^2/2$ for $k = \pm 1$ and 0 otherwise. We therefore divide each term P_k by P_1 to normalize the power spectrum. Since the $k = 1$ term always contributes a value of 1 to the sum, it is omitted from further consideration.

We incorporated two additional factors in the spectral characterization of invasion based on means and parametric derivatives estimated at the midpoint of each interval:

$$\bar{x}_{j'} \equiv (x_{j'+1/2} + x_{j'-1/2})/2, \tag{3}$$
$$\bar{y}_{j'} \equiv (y_{j'+1/2} + y_{j'-1/2})/2, \tag{4}$$
$$\dot{x}_{j'} \equiv (x_{j'+1/2} - x_{j'-1/2})/(2\pi/M), \tag{5}$$
$$\dot{y}_{j'} \equiv (y_{j'+1/2} - y_{j'-1/2})/(2\pi/M), \tag{6}$$

with $j' \in \{1/2, 3/2, \ldots, M - 1/2\}$. The mappings $\bar{x}$ and $\bar{y}$ smooth artifacts in the boundary due to pixelation. A zigzag or staircase motif with (x, y) coordinates $(0,0),\ (1,0),\ (1,1)\,(2,1)$, for example, is mapped to the line $\bar{y} = \bar{x} - 1/2$.

The transform of $\bar{x}$ is

$$\begin{aligned} \hat{\bar{x}}_k &= \sum_{j'=1/2}^{M-1/2} e^{-2\pi ij'k/M} \bar{x}_{j'} \\ &= (1/2) \sum_{j'=1/2}^{M-1/2} e^{-2\pi ij'k/M} x_{j'+1/2} + (1/2) \sum_{j'=1/2}^{M-1/2} e^{-2\pi ij'k/M} x_{j'-1/2} \\ &= (e^{\pi ik/M}/2) \sum_{j=1}^{M} e^{-2\pi ijk/M} x_j + (e^{-\pi ik/M}/2) \sum_{j=0}^{M-1} e^{-2\pi ijk/M} x_j \\ &= \cos(\pi k/M) \hat{x}_k, \end{aligned} \tag{7}$$

the cosine low-pass filter. We have used the periodic property that $e^{2\pi ijk/M} x_j$ is identical for $j = 0$ and $j = M$. Using this filter, $P_k \to \cos^2(\pi k/M) P_k$, which smoothly switches the power to 0 as the frequency approaches its largest magnitude, $|k| = M/2$.

The mappings $\dot{x}$ and $\dot{y}$ are the local tangents to the parametric curve, normalized by the constant distance $2\pi/M$ between adjacent interpolation points. Derivatives of parametric curves are related to curvature, which is constant for a non-invasive round shape and varies over the boundary for an invasive structure. With a similar approach, the transform of $\dot{x}$ is

$$\begin{aligned}\hat{\dot{x}}_k &= (M/2\pi)(e^{\pi ik/M} - e^{-\pi ik/M}) \\ &= (iM/\pi)\sin(\pi k/M)\hat{x}_k. \end{aligned} \tag{8}$$

We therefore introduce the weight $(M/\pi)^2 \sin^2(\pi k/M)$. For low frequencies, $\sin(\pi k/M) \approx \pi k/M$, and $\hat{\dot{x}}_k \to ik\hat{x}_k$, the analogous result for continuous coordinates. In this limit, the weighting factor is k^2.

We combined these transforms and the normalization by the power of the first mode to arrive at a weighted spectral power, w, defined as

$$w \equiv \sum_{k=2}^{M/2} (M/\pi)^2 \sin^2(\pi k/M)\cos^2(\pi k/M) P_k/P_1. \tag{9}$$

For small values of k, and defining the spacing $2\pi/M$ as λ, the leading terms of the weighting and filtering factors are

$$\begin{aligned}\lambda^{-2}\sin^2(\lambda k) &\approx \lambda^{-2}[\lambda k - \lambda^3 k^3/6]^2 \\ &= k^2[1-\lambda^2 k^2/6]^2 \\ &\approx k^2 e^{-(2/3)\lambda^2 k^2}, \end{aligned} \tag{10}$$

a weight of k^2 and a Gaussian low-pass filter over a width on the order of the spacing $2\pi/M$ between interpolation points.

2 Bootstrapped Bayesian model selection

The normal mixture models are

$$M_0: \quad \Pr(\mathbf{D}|\mu_0, \sigma_0^2) = \prod_{t=1}^{T}\prod_{i=1}^{N_t} (2\pi\sigma_0^2)^{-1/2} \exp[-(y_{it}-\mu_0)^2/2\sigma_0^2]; \tag{11}$$

$$M_1: \quad \Pr(\mathbf{D}|\{\mu_t\}, \sigma_W^2) = \prod_{t=1}^{T}\prod_{i=1}^{N_t} (2\pi\sigma_W^2)^{-1/2} \exp[-(y_{it}-\mu_t)^2/2\sigma_W^2]; \tag{12}$$

$$M_2: \quad \Pr(\mathbf{D}|\{\mu_t\}, \{\sigma_t^2\}) = \prod_{t=1}^{T}\prod_{i=1}^{N_t} (2\pi\sigma_t^2)^{-1/2} \exp[-(y_{it}-\mu_t)^2/2\sigma_t^2]. \tag{13}$$

The maximum likelihood parameters for these normal mixture models are

$$\begin{aligned}
\tilde{\mu}_0 &= N^{-1} \sum_{t=1}^{T} \sum_{i=1}^{N_t} y_{ti}, && (14) \\
\tilde{\mu}_t &= N_t^{-1} \sum_{i=1}^{N_t} y_{ti}, && (15) \\
\tilde{\sigma}_0^2 &= N^{-1} \sum_{t=1}^{T} \sum_{i=1}^{N_t} (y_{ti} - \tilde{\mu}_0)^2, && (16) \\
\tilde{\sigma}_W^2 &= N^{-1} \sum_{t=1}^{T} \sum_{i=1}^{N_t} (y_{ti} - \tilde{\mu}_t)^2, && (17) \\
\tilde{\sigma}_t^2 &= N_t^{-1} \sum_{i=1}^{N_t} (y_{ti} - \tilde{\mu}_t)^2. && (18)
\end{aligned}$$

The resulting model scores are

$$\begin{aligned}
S_0 &= -(N/2)\ln(2\pi\tilde{\sigma}_0^2) - N/2 - \ln N, && (19) \\
S_1 &= -(N/2)\ln(2\pi\tilde{\sigma}_W^2) - N/2 - (1/2)\ln N - (1/2)\sum_{t=1}^{T} \ln N_t, && (20) \\
S_2 &= \sum_{t=1}^{T} -(N_t/2)\ln(2\pi\tilde{\sigma}_t^2) - N_t/2 - \ln N_t. && (21)
\end{aligned}$$

For S_0, all N observations contribute to the two parameters μ_0 and $\tilde{\sigma}_0^2$. For S_1, all N observations contribute to estimating $\tilde{\sigma}_W^2$, and N_t observations contribute to each estimated $\tilde{\mu}_t$. For S_2, N_t observations contribute to each $\tilde{\mu}_t$ and $\tilde{\sigma}_t^2$. For tumors with a single organoid, we used the shared variance estimate rather than the within-tumor estimate.

3 Variance components model

The variance components model considers hypotheses H_0 and H_1 equivalent to models M_0 and M_1:

$$\begin{aligned}
H_0 &: \quad y_{ti} = \mu_0 + \epsilon_{ti}, \quad \epsilon_{ti} \sim \text{Norm}(0, \sigma_0^2); && (22) \\
H_1 &: \quad y_{ti} = \mu_t + \epsilon_{ti}, \quad \epsilon_{ti} \sim \text{Norm}(0, \sigma_W^2). && (23)
\end{aligned}$$

Unbiased estimates of means are

$$\begin{aligned}
\hat{\mu}_0 &= (1/N) \sum_{t=1}^{T} \sum_{i=1}^{N_t} y_{ti}, && (24) \\
\hat{\mu}_t &= (1/N_t) \sum_{i=1}^{N_t} y_{ti}. && (25)
\end{aligned}$$

The notation $\hat{\mu}$ rather than $\tilde{\mu}$ indicates unbiased estimates rather than maximum likelihood estimates. For means these estimates are identical, but for variances they are different. The sums of squares Σ_0 for the total population, Σ_M for the modeled

between-tumor effects, Σ_t for individual tumors, and Σ_W for within-tumor are

$$\begin{aligned}
\Sigma_0 &= \sum_{t=1}^{T}\sum_{i=1}^{N_T}(y_{ti}-\hat{\mu}_0)^2, && (26)\\
\Sigma_M &= \sum_{t=1}^{T} N_t(\hat{\mu}_t-\hat{\mu}_0)^2, && (27)\\
\Sigma_t &= \sum_{i=1}^{N_t}(y_{ti}-\hat{\mu}_t)^2, && (28)\\
\Sigma_W &= \sum_{t=1}^{T}\Sigma_t, && (29)
\end{aligned}$$

with $\Sigma_M + \Sigma_W = \Sigma_0$. The unbiased estimates of variance for H_0 and H_1, and the variance σ_M^2 from the modeled between-tumor effects, are

$$\begin{aligned}
\hat{\sigma}_0^2 &= \Sigma_0/(N-1), && (30)\\
\hat{\sigma}_W^2 &= \Sigma_W/(N-K), && (31)\\
\hat{\sigma}_M^2 &= \Sigma_M/(T-1). && (32)
\end{aligned}$$

The ANOVA test statistic, Q_1, is

$$Q_1 = \hat{\sigma}_M^2/\hat{\sigma}_W^2. \quad (33)$$

Under the null hypothesis of equal means, $\mu_1 = \mu_2 = \ldots = \mu_T$, Q_1 is a random variable following an F-distribution,

$$Q_1 \sim F_{T-1,N-K}. \quad (34)$$

If the null hypothesis is rejected, then the invasiveness of an organoid may be described as a random variable y composed of a between-tumor effect ϵ_B and a within-tumor effect ϵ_W, with

$$\begin{aligned}
y &= \epsilon_B + \epsilon_W, && (35)\\
\epsilon_B &\sim \mathrm{Norm}(\mu_0, \sigma_B^2), && (36)\\
\epsilon_W &\sim \mathrm{Norm}(0, \sigma_W^2), && (37)\\
y &\sim \mathrm{Norm}(\mu_0, \sigma_B^2 + \sigma_W^2), && (38)\\
\sigma_0^2 &= \sigma_B^2 + \sigma_W^2. && (39)
\end{aligned}$$

The unbiased estimator $\hat{\sigma}_B^2$ is

$$\begin{aligned}
\hat{\sigma}_B^2 &= \hat{\sigma}_0^2 - \hat{\sigma}_W^2\\
&= \frac{\Sigma_M + \Sigma_W}{N-1} - \frac{\Sigma_W}{N-T}\\
&= \frac{1}{N-1}\left[\Sigma_M - \frac{T-1}{N-T}\Sigma_W\right]\\
&= \frac{1}{N-1}\left[\Sigma_M - (T-1)\hat{\sigma}_W^2\right]. && (40)
\end{aligned}$$

Of the total variance, $\hat{\sigma}_B^2$ is estimated to arise from between-tumor heterogeneity, and $\hat{\sigma}_W^2$ from within-tumor heterogeneity.

Power analysis

The mixed effect model is

$$y_{ti} = \mu_t + \beta x_{ti} + \epsilon_{ti},\ \epsilon_{ti} \sim \mathrm{Norm}(0, \sigma_\epsilon^2). \tag{41}$$

Tumor means and organoid deviations from the mean are

$$\begin{aligned}
\bar{y}_t &= N_t^{-1} \sum_{i=1}^{N_t} y_{ti}, && (42)\\
\bar{x}_t &= N_t^{-1} \sum_{i=1}^{N_t} x_{ti}, && (43)\\
\delta y_{ti} &= y_{ti} - \bar{y}_t, && (44)\\
\delta x_{ti} &= x_{ti} - \bar{x}_t. && (45)
\end{aligned}$$

These transformations define two models, a between-tumor model for the means and a within-tumor model for the deviations:

$$\begin{aligned}
\bar{y}_t &= \mu_t + \beta_B \bar{x}_t + \bar{\epsilon}_t,\ \bar{\epsilon}_t \sim \mathrm{Norm}[0, N_t^{-1}\sigma_\epsilon^2]; && (46)\\
\delta y_{ti} &= \beta_W \delta x_{ti} + \delta\epsilon_{ti},\ \delta\epsilon_{ti} \sim \mathrm{Norm}[0, (1 - N_t^{-1})\sigma_\epsilon^2]. && (47)
\end{aligned}$$

Here we have represented the parameter β from the mixed effects model as two separate parameters, β_B for the between-tumor test and β_W for the within-tumor tests. These tests are conducted separately as follows.

For the between-tumor test, means and sums of squares are

$$\begin{aligned}
\hat{\bar{y}} &= T^{-1} \sum_t \bar{y}_t, && (48)\\
\Sigma_{\bar{y}\bar{y}} &= \sum_{t=1}^{T} (\bar{y}_t - \hat{\bar{y}})^2, && (49)\\
\Sigma_{\bar{y}\bar{x}} &= \sum_{t=1}^{T} (\bar{y}_t - \hat{\bar{y}})(\bar{x} - \hat{\bar{x}}), && (50)\\
\Sigma_{\bar{x}\bar{x}} &= \sum_{t=1}^{T} (\bar{x}_t - \hat{\bar{x}})^2, && (51)\\
\hat{\beta}_B &= \Sigma_{\bar{y}\bar{x}} / \Sigma_{\bar{x}\bar{x}}, && (52)\\
\hat{R}_B &= \Sigma_{\bar{y}\bar{x}} / \sqrt{\Sigma_{\bar{y}\bar{y}} \Sigma_{\bar{x}\bar{x}}}, && (53)\\
\hat{Q}_B &= \hat{R}_B \sqrt{(T-1)/(1 - \hat{R}_B^2)}. && (54)
\end{aligned}$$

Under the null hypothesis that $\beta_B = 0$, the test statistic $\hat{Q}_B^2 \sim F_{1,T-1}$, which for large T is distributed approximately as χ_1^2. Under the alternative hypothesis, $\hat{Q}_B^2$ is distributed as a non-central χ_1^2 distribution whose expected value depends on the true fraction of variance explained by the between-tumor model, R_B^2,

$$\hat{Q}_B \sim \mathrm{Norm}\left(R_B \sqrt{(T-1)/(1 - R_B^2)}, 1 \right). \tag{55}$$

Define the cumulative probability integral $\Phi(z)$ as

$$\Phi(z) = (2\pi)^{-1/2} \int_{-\infty}^{z} du\, \exp(-u^2/2), \tag{56}$$

with $\Phi^{-1}(\alpha)$ giving the value of z with lower-tail area α. A two-tailed test of $\beta_B \neq 0$ with type I error controlled at level α corresponds to $|\hat{Q}_B| > z_I$, with

$$z_I = \Phi^{-1}(1-\alpha/2). \tag{57}$$

Given the true value Q_B, the type II error rate (or false-negative rate) is $\Phi(z_I - |Q_B|)$, with corresponding quantile

$$z_{II} = z_I - |Q_B|. \tag{58}$$

The relationship between the type I and II error rates, the true effect size R_B, and the population size (number of tumors T) is summarized as

$$Q_B^2 = (T-1)R_B^2/(1-R_B^2) = (z_I - z_{II})^2. \tag{59}$$

This expression depends explicitly on the number of tumors, T. It depends implicitly on the number of organoids per tumor through the factor R_B^2, which is inversely proportional to the variance of the estimated tumor-level invasiveness. This variance is $\sigma_B^2 + \sigma_W^2/N_t$. We use R_B^2 to represent the variance explained in the limit of large N_t, and for simplicity assume that the number of organoids per tumor is the same for all tumors, $N_t = N/T$ for all t. For a particular choice of N_t, the variance explained is reduced by the factor $1 + (\sigma_W^2/N_t\sigma_B^2)$. The explicit dependence of power on T and N_t is then

$$Q_B^2 = (T-1)\frac{R_B^2/[1+(\sigma_W^2/N_t\sigma_B^2)]}{1-R_B^2/[1+(\sigma_W^2/N_t\sigma_B^2)]}(z_I - z_{II})^2, \tag{60}$$

where R_B^2 represents the variance explained in the limit of large N_t and perfect knowledge of the tumor mean invasiveness.

The within-tumor test follows a similar pattern. The sums of squares and estimates are

$$\Sigma_{\delta y \delta y} = \sum_{t=1}^{T}\sum_{i=1}^{N_t} \delta y_{ti}^2, \tag{61}$$

$$\Sigma_{\delta y \delta x} = \sum_{t=1}^{T}\sum_{i=1}^{N_t} \delta y_{ti}\delta x_{ti}, \tag{62}$$

$$\Sigma_{\delta x \delta x} = \sum_{t=1}^{T}\sum_{i=1}^{N_t} \delta x_{ti}^2, \tag{63}$$

$$\hat{\beta}_W = \Sigma_{\delta y \delta x}/\Sigma_{\delta x \delta x}, \tag{64}$$

$$\hat{R}_W = \Sigma_{\delta y \delta x}/\sqrt{\Sigma_{\delta y \delta y}\Sigma_{\delta x \delta x}}, \tag{65}$$

$$\hat{Q}_W = \hat{R}_W\sqrt{(N-T-1)/(1-\hat{R}_W^2)}. \tag{66}$$

In this case, $\hat{Q}_W^2 \sim F_{1,N-T-1}$. The relationship between error rates, effect size, and population size is

$$Q_W^2 = (N-T-1)R_W^2/(1-R_W^2) = (z_I - z_{II})^2. \tag{67}$$
