# Supplementary material for: Between-tumor and within-tumor heterogeneity in invasive potential
Source: PLoS Comput Biol. 2020 Jan 21;16(1):e1007464. doi: 10.1371/journal.pcbi.1007464 (PMC6994152; doi:10.1371/journal.pcbi.1007464)
Supplement: S1 File — (GZ) [file pcbi.1007464.s003.tar.gz › S1_File/OUTPUT_FIG1/fig7_CTN010.pdf]

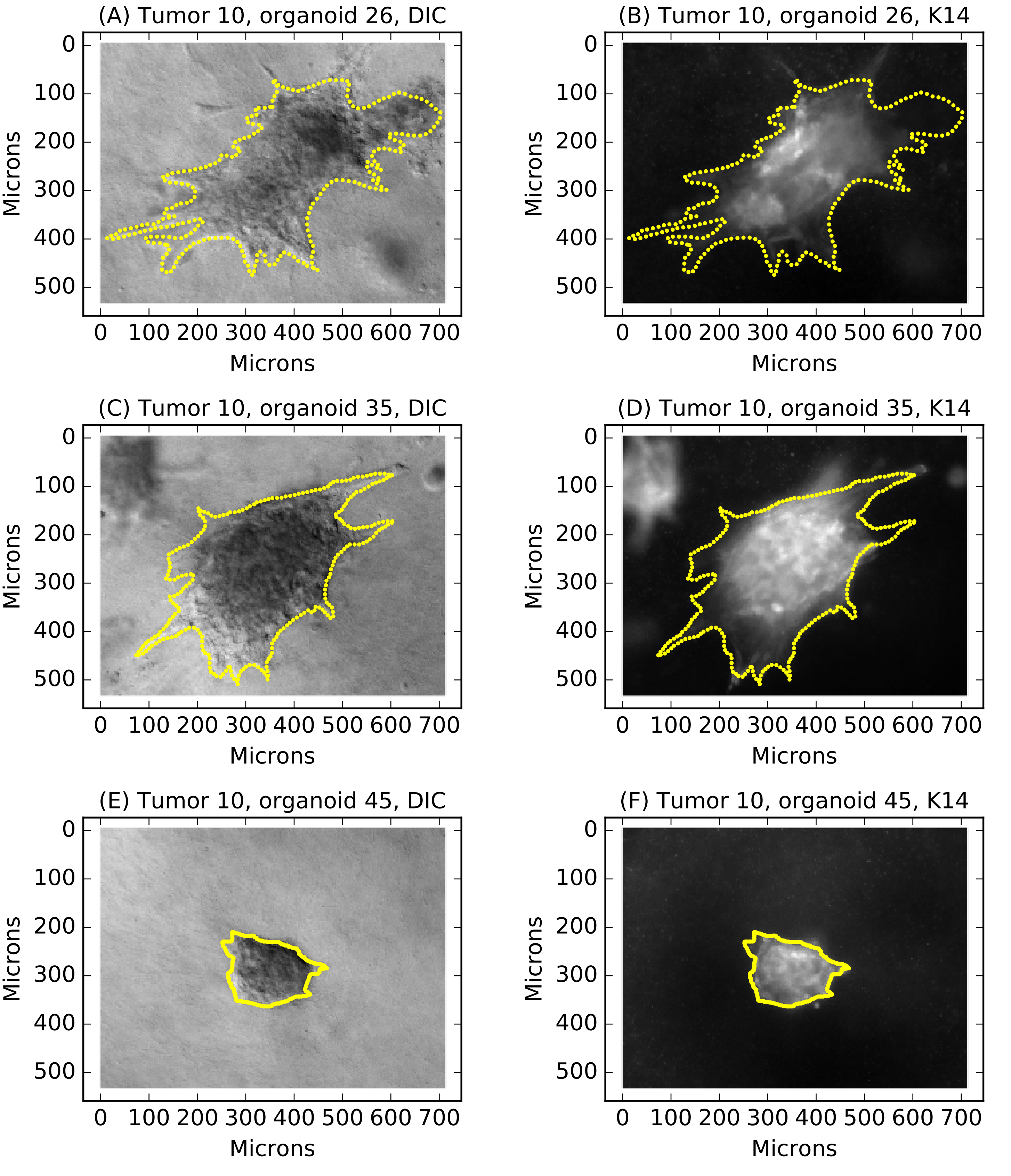
(A) Tumor 10, organoid 26, DIC
(B) Tumor 10, organoid 26, K14
(C) Tumor 10, organoid 35, DIC
(D) Tumor 10, organoid 35, K14
(E) Tumor 10, organoid 45, DIC
(F) Tumor 10, organoid 45, K14
Microns
0
100
200
300
400
500
600
700
