# Supplementary figures and images for: Between-tumor and within-tumor heterogeneity in invasive potential
Source: PLoS Comput Biol. 2020 Jan 21;16(1):e1007464. doi: 10.1371/journal.pcbi.1007464 (PMC6994152; doi:10.1371/journal.pcbi.1007464)

(A) Invasion, arithmetic scale

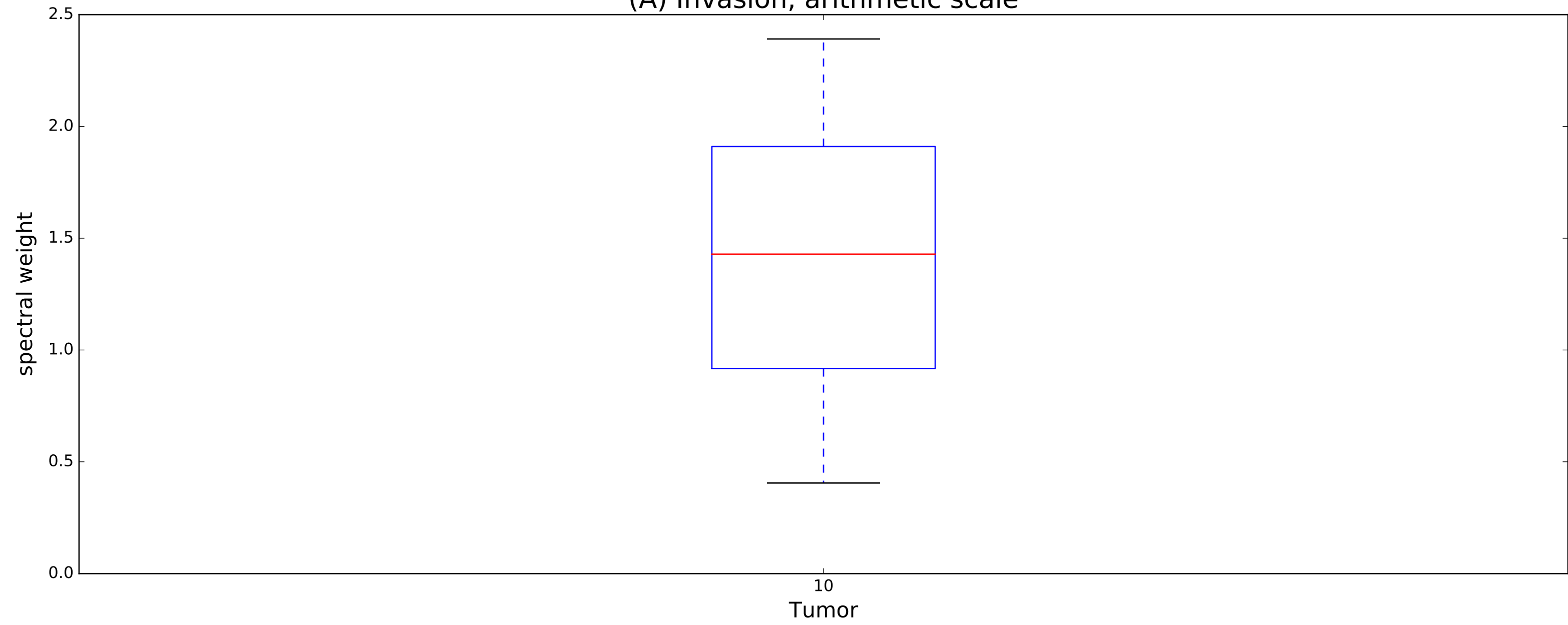

(B) Invasion, logarithmic scale

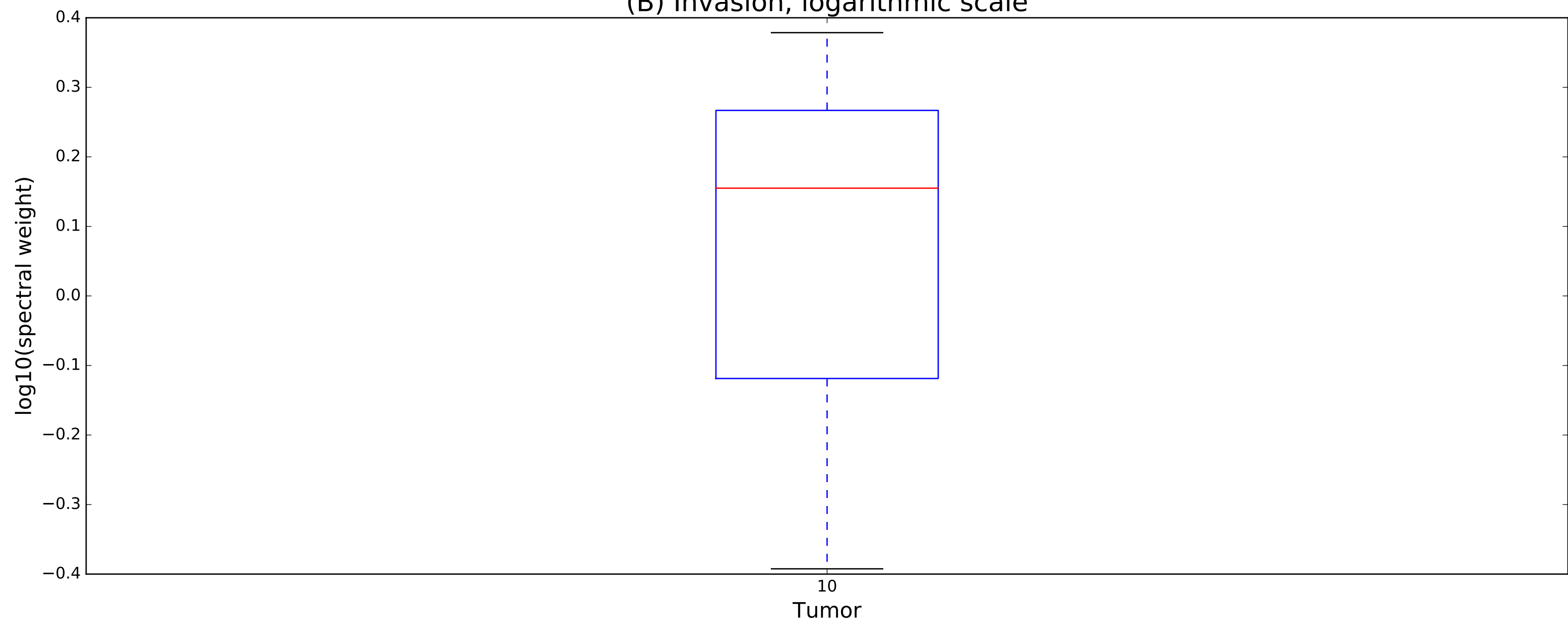

Supplement: S1 File — (GZ) [file pcbi.1007464.s003.tar.gz › S1_File/OUTPUT_FIG1/fig4_boxplot.pdf]

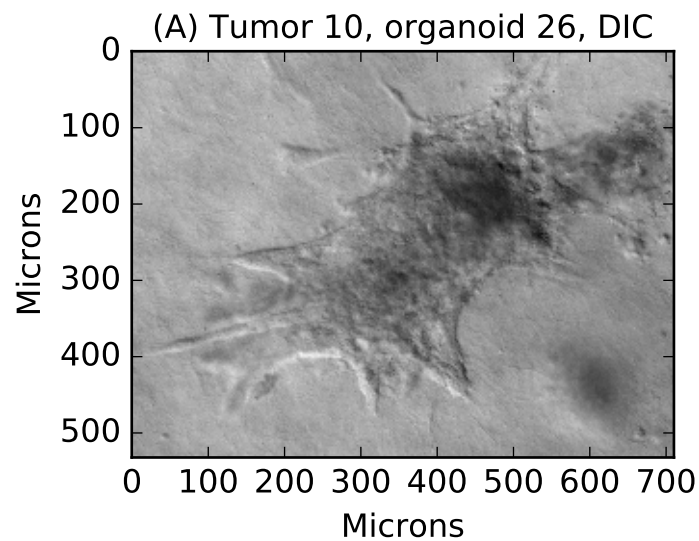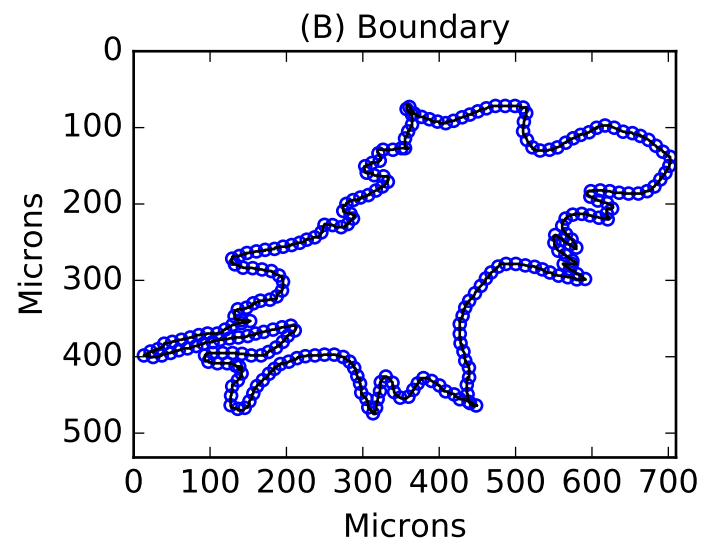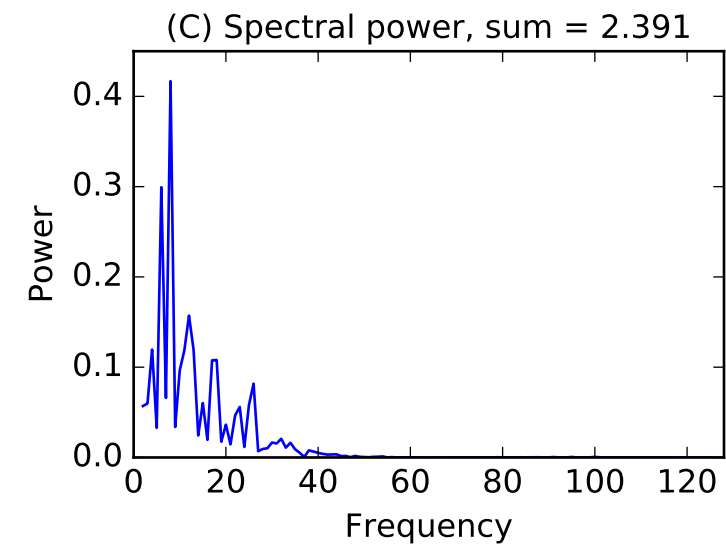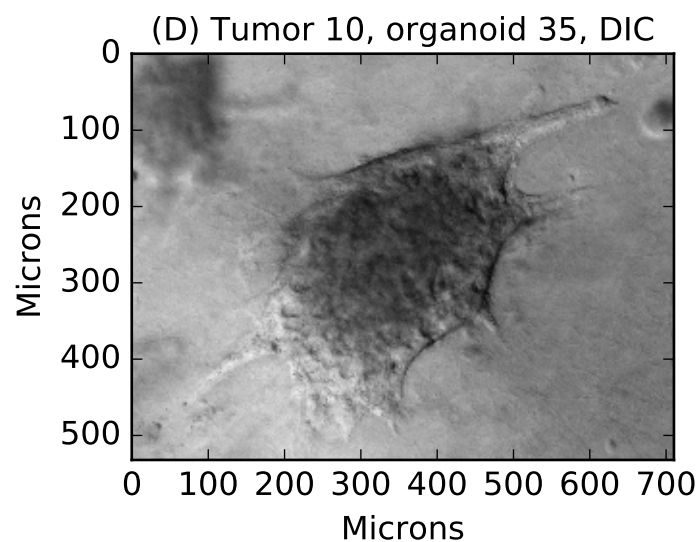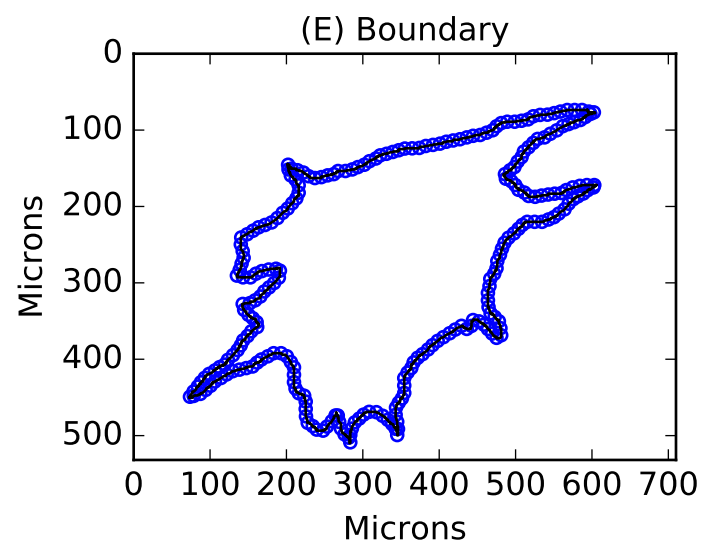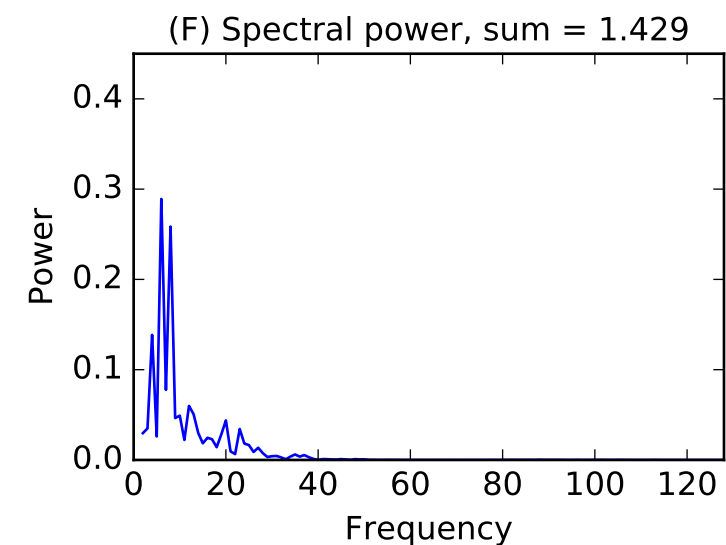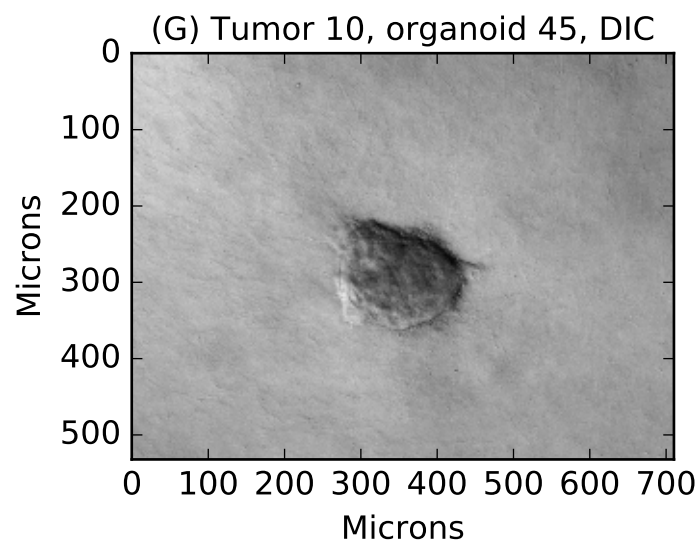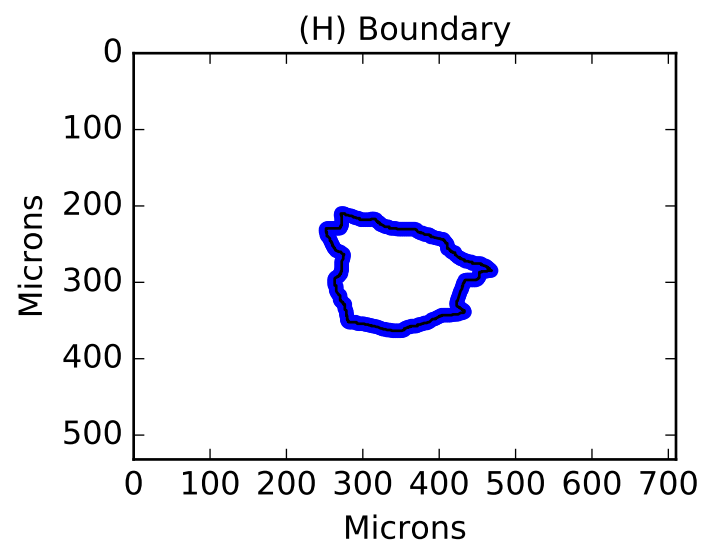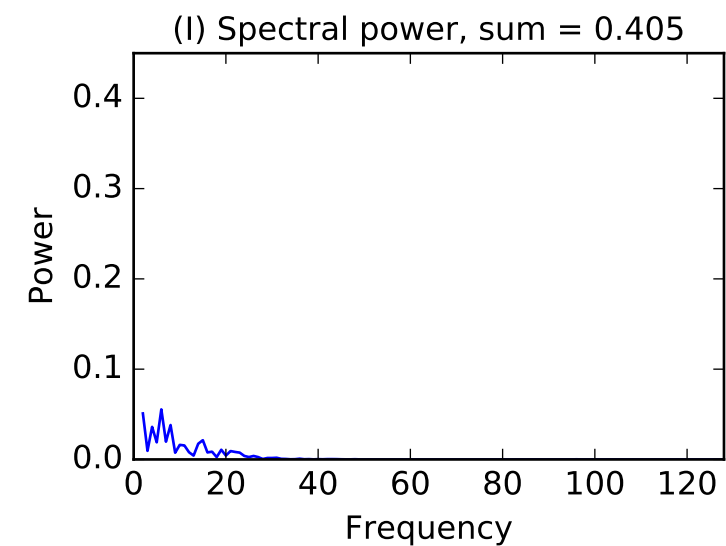

Supplement: S1 File — (GZ) [file pcbi.1007464.s003.tar.gz › S1_File/OUTPUT_FIG1/fig1_CTN010.pdf]

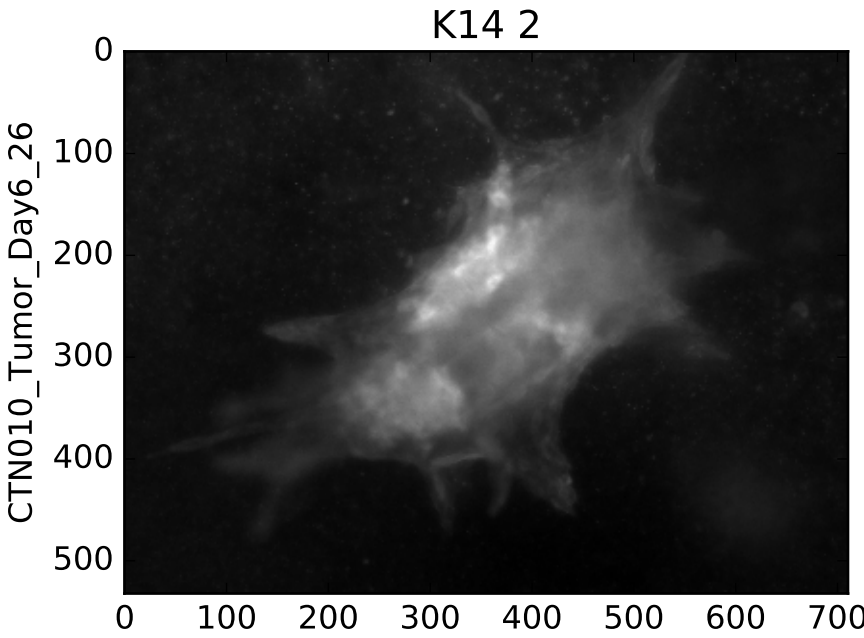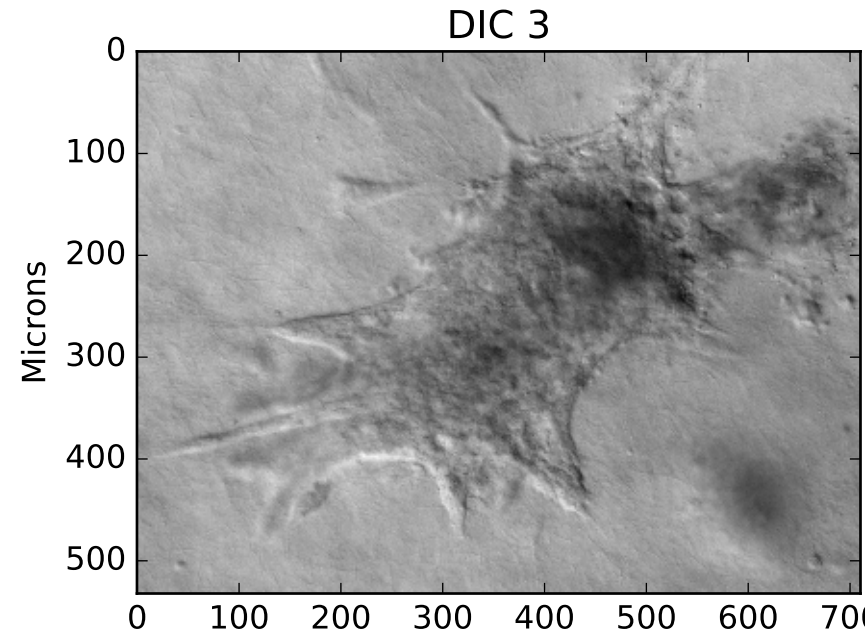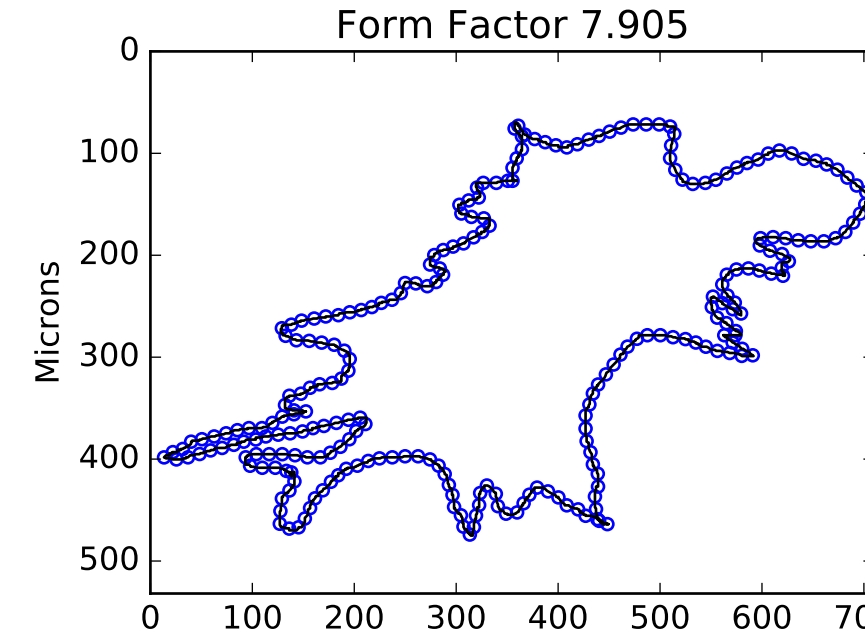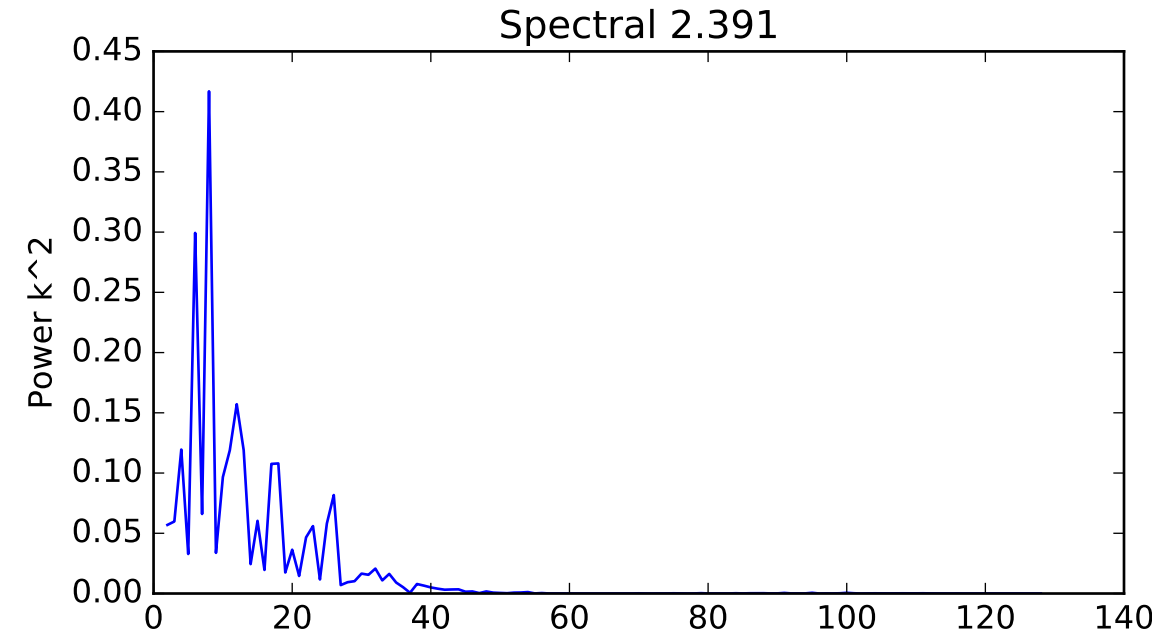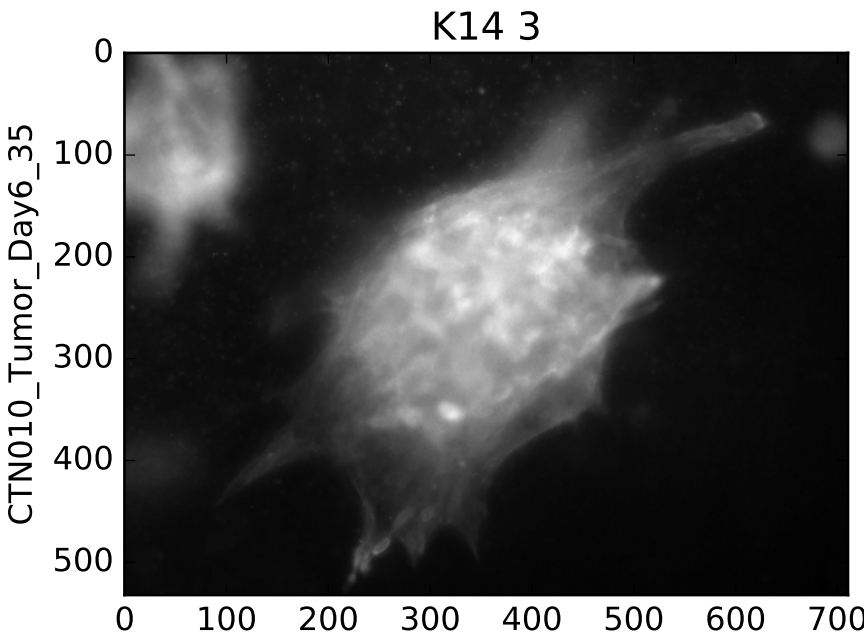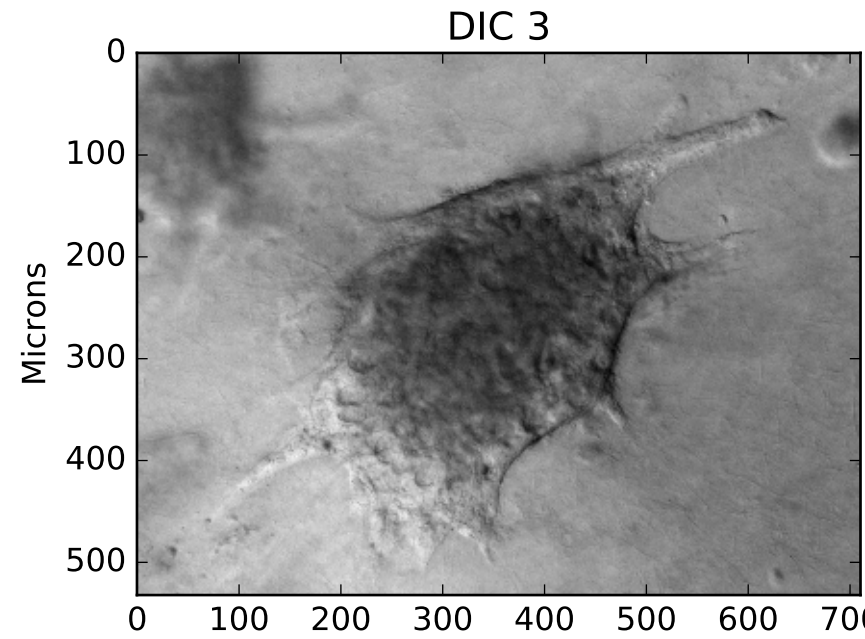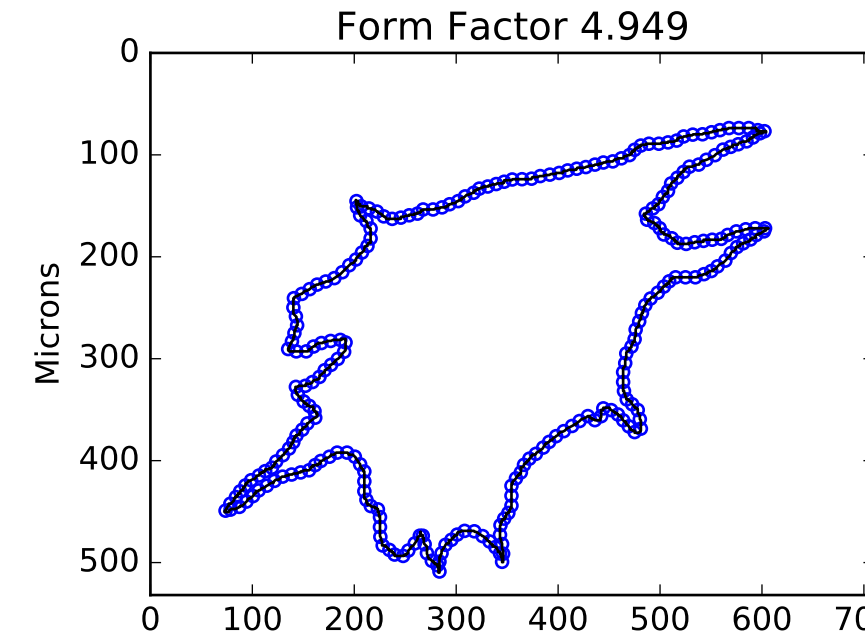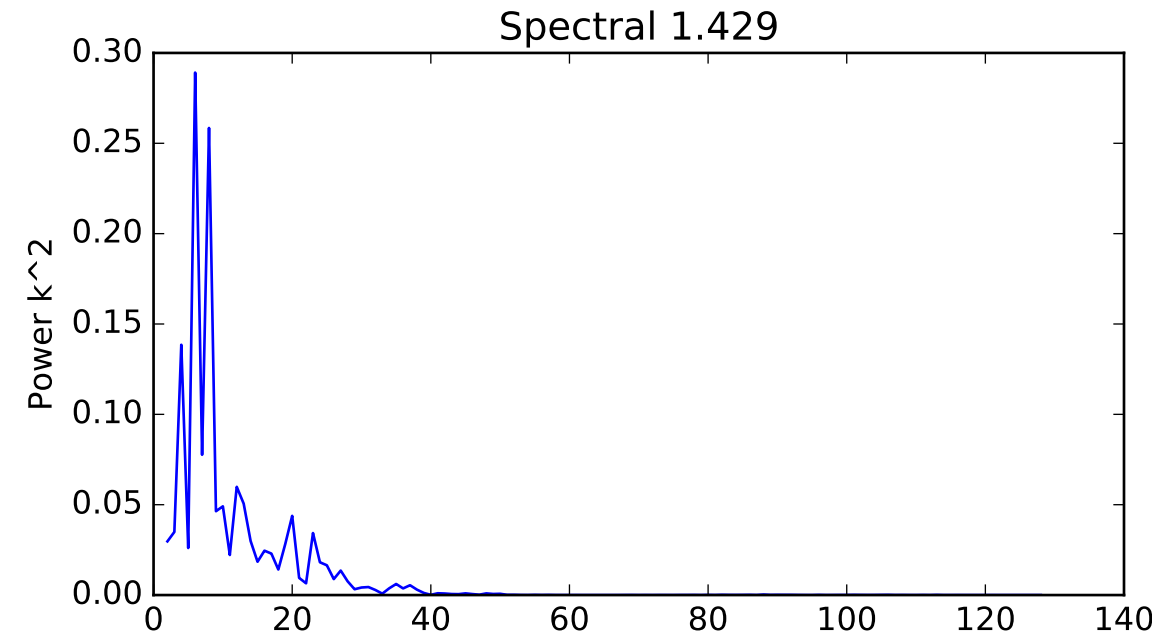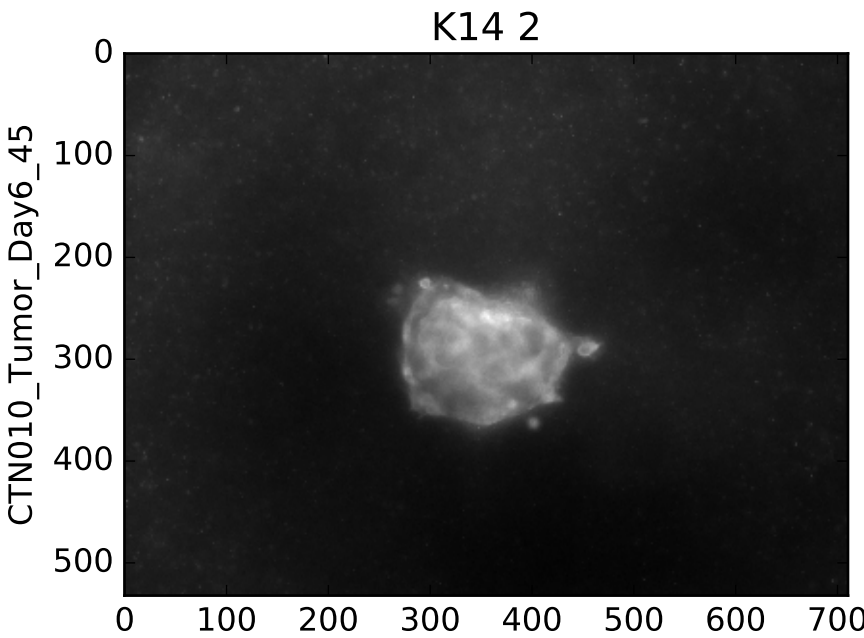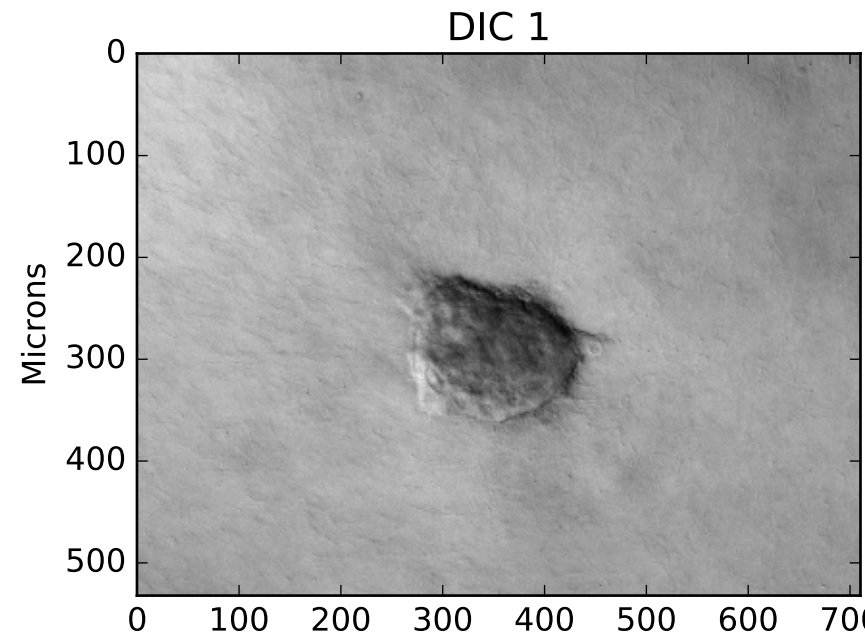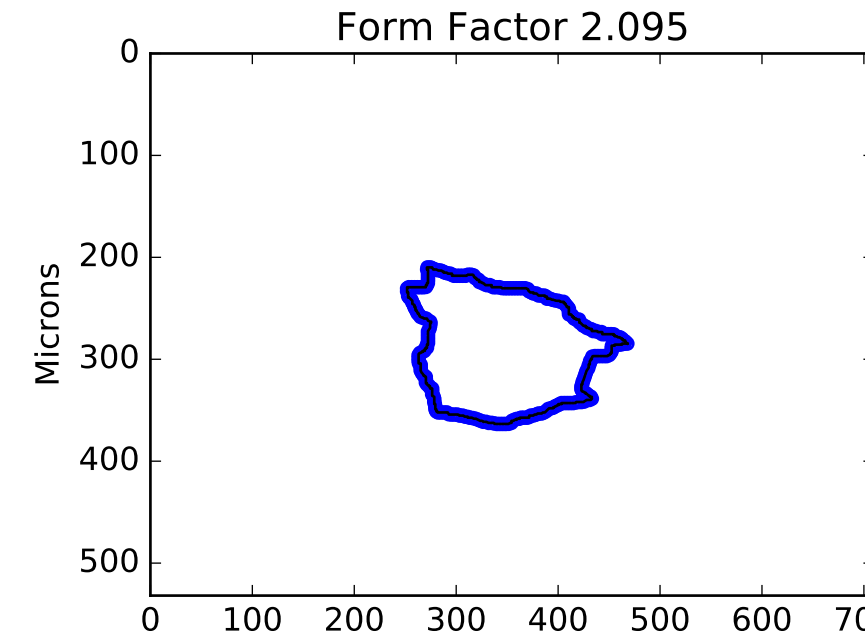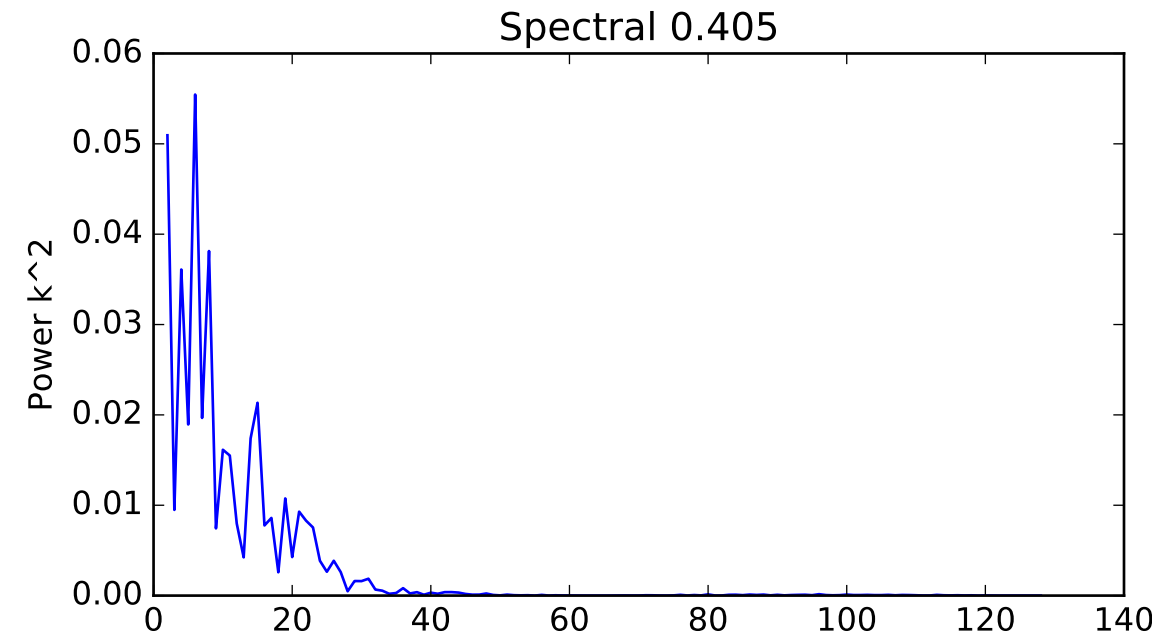

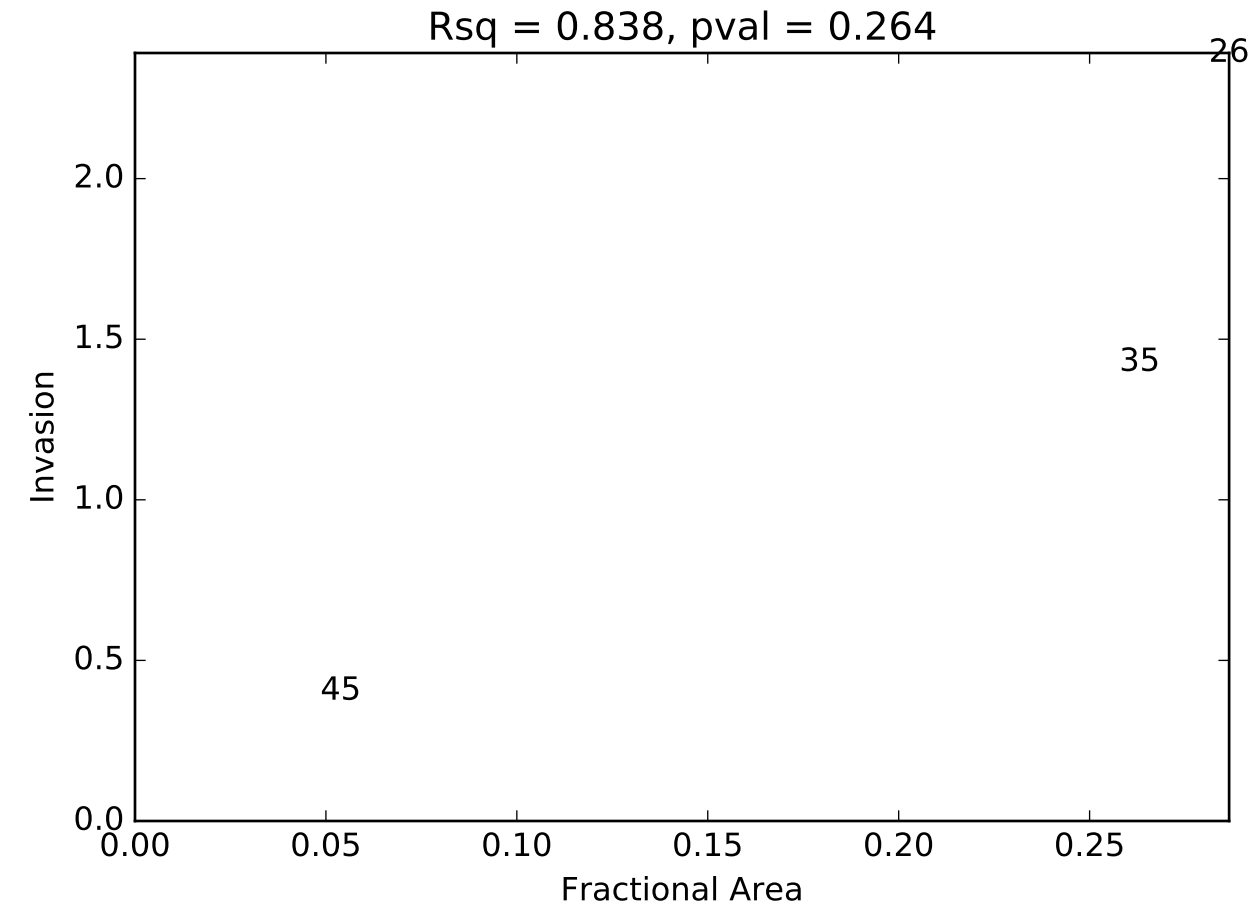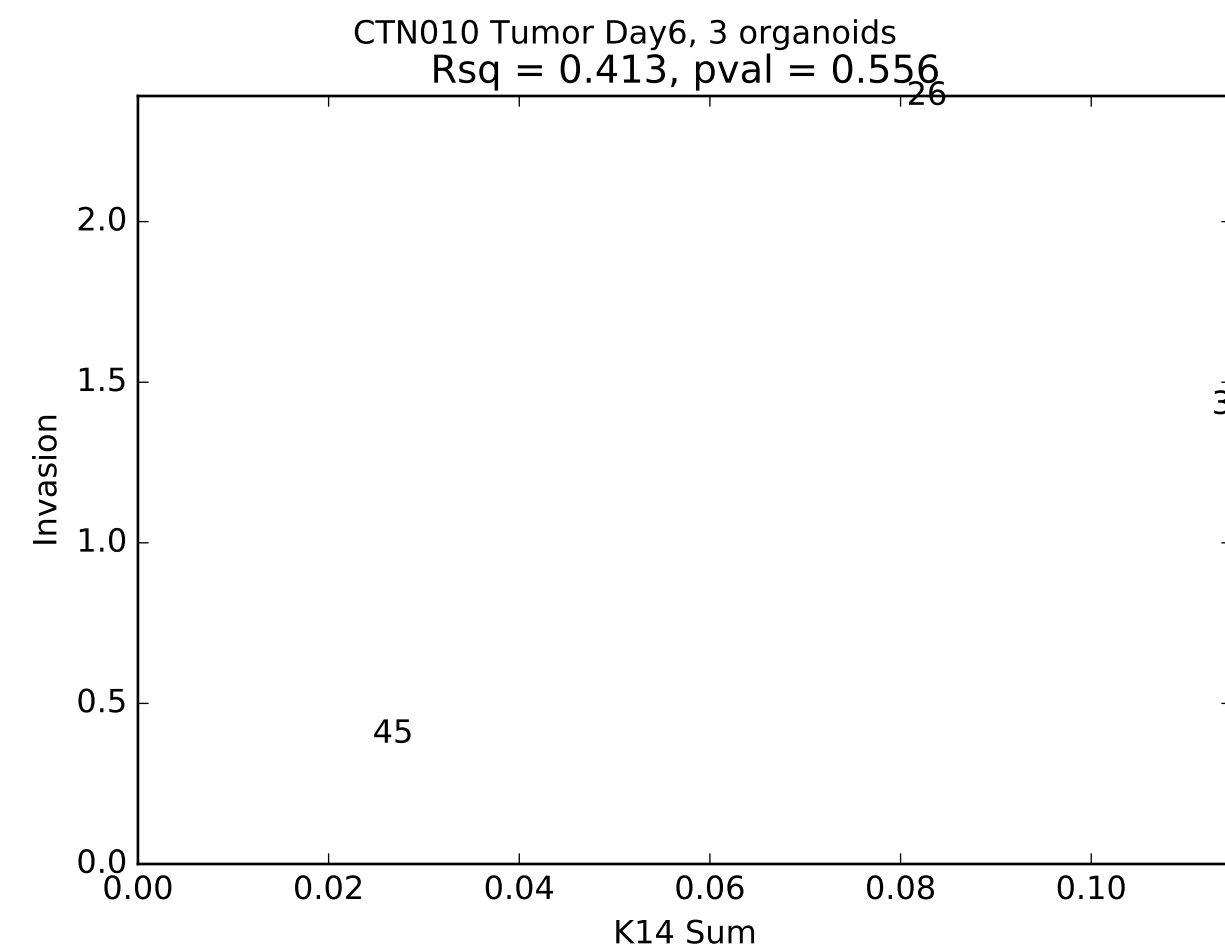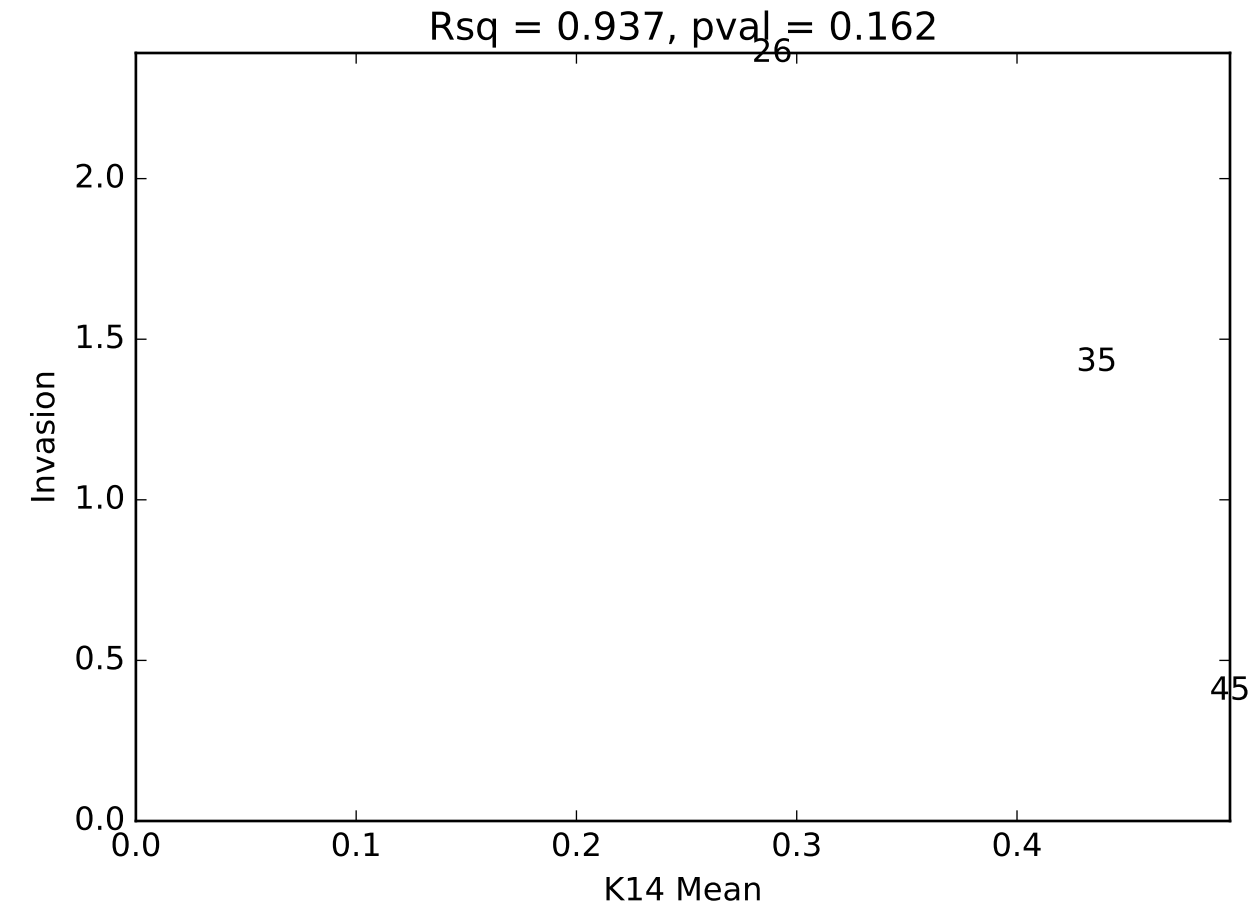

Supplement: S1 File — (GZ) [file pcbi.1007464.s003.tar.gz › S1_File/OUTPUT_FIG1/organoids.pdf]

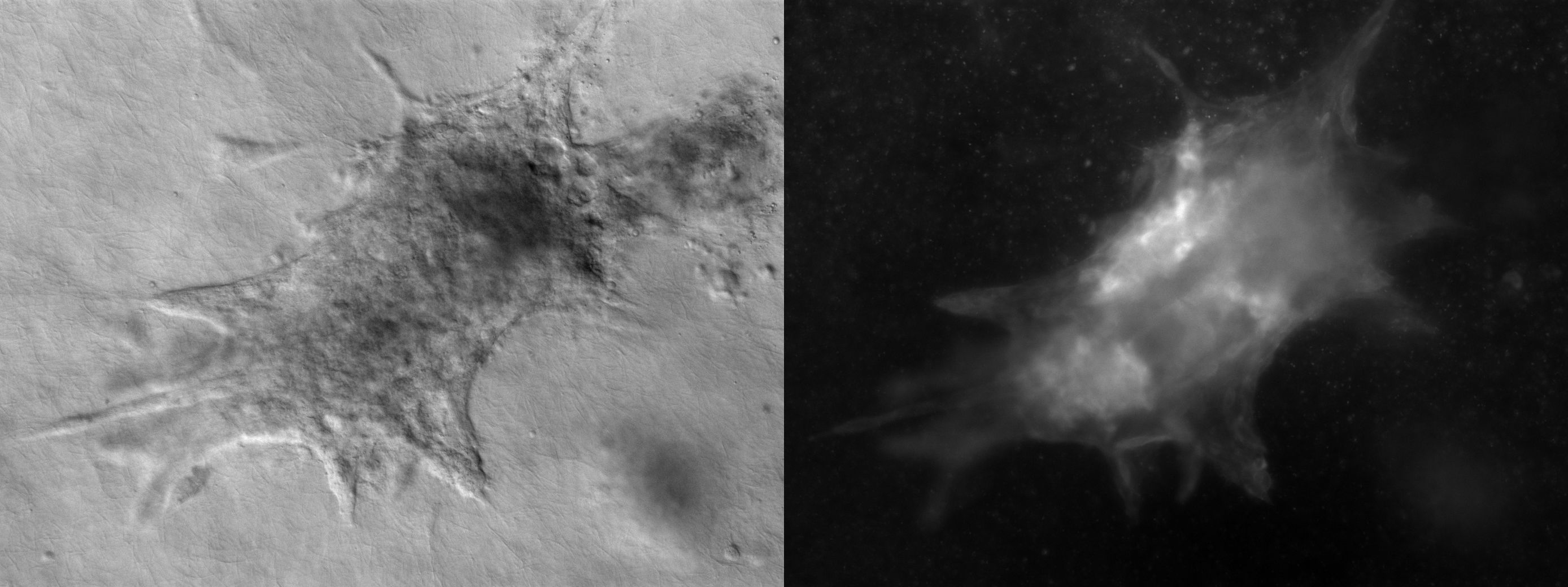

Supplement: S1 File — (GZ) [file pcbi.1007464.s003.tar.gz › S1_File/IMAGES_FIG1/CTN010_Day6-003_AC04_26.zvi.tif]

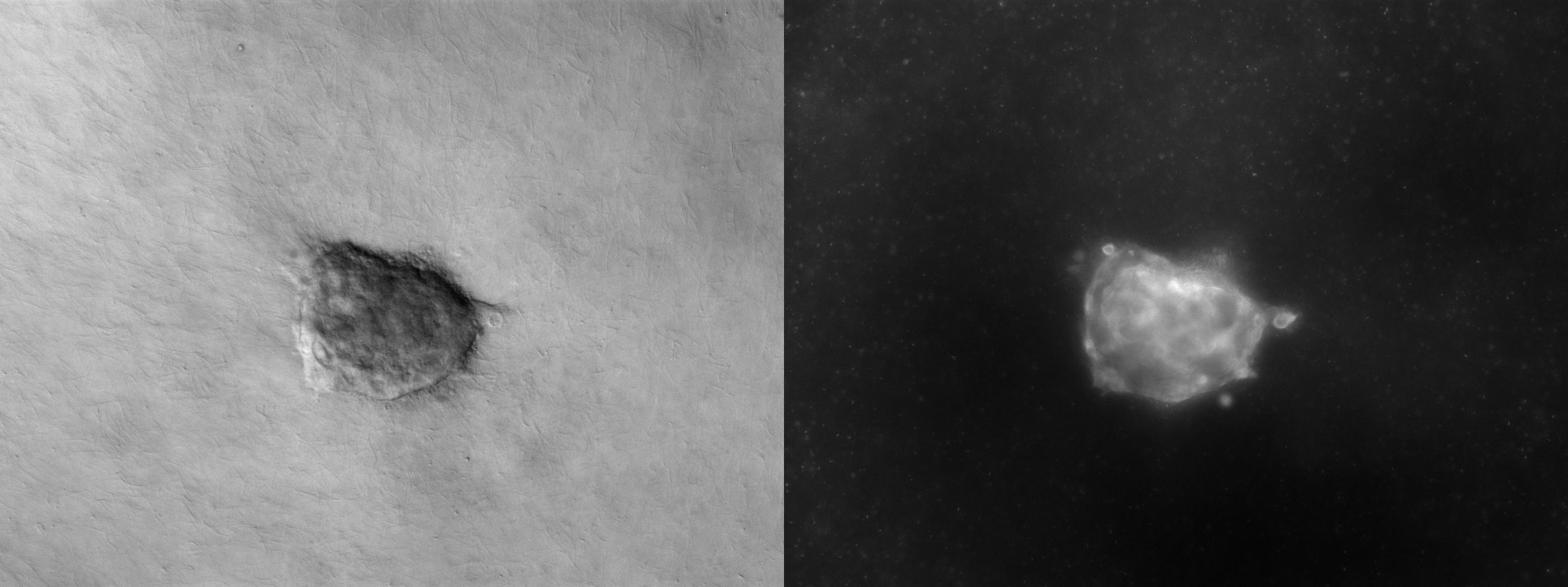

Supplement: S1 File — (GZ) [file pcbi.1007464.s003.tar.gz › S1_File/IMAGES_FIG1/CTN010_Day6-003_AC04_45.zvi.tif]

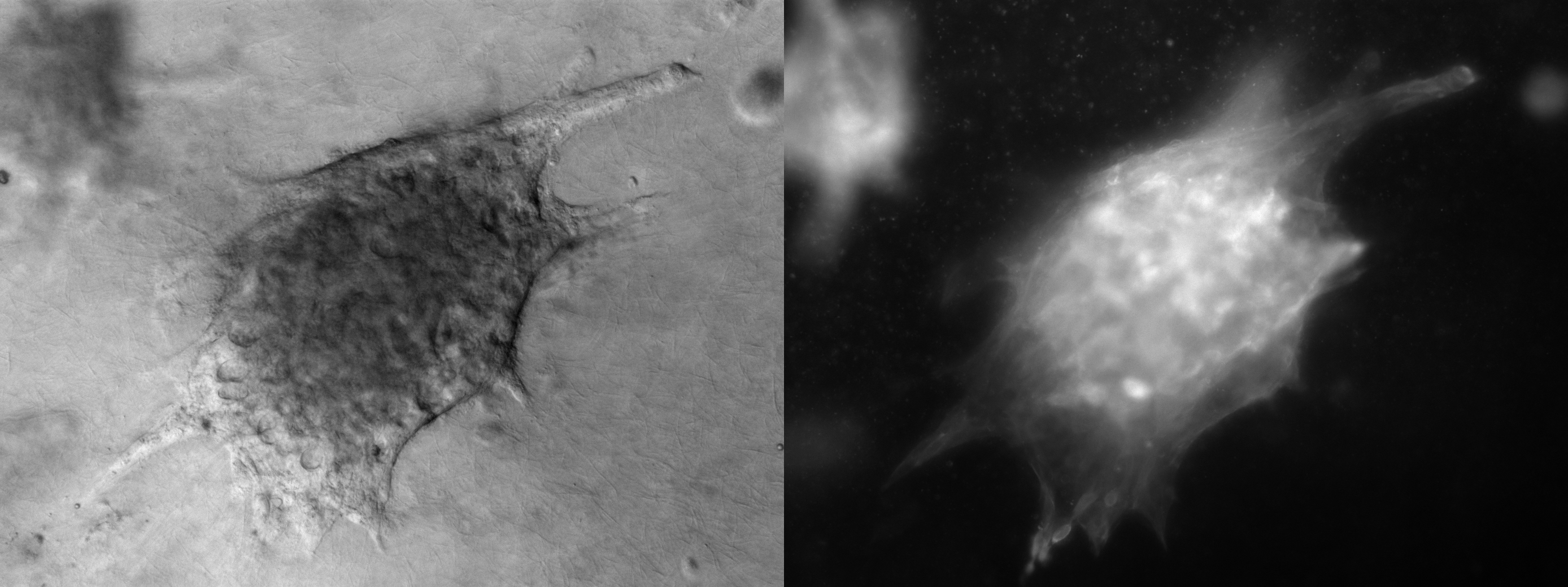

Supplement: S1 File — (GZ) [file pcbi.1007464.s003.tar.gz › S1_File/IMAGES_FIG1/CTN010_Day6-003_AC04_35.zvi.tif]

(A) Between-tumor test,  $R^2 = 0.05$ ,  $p = 0.14$

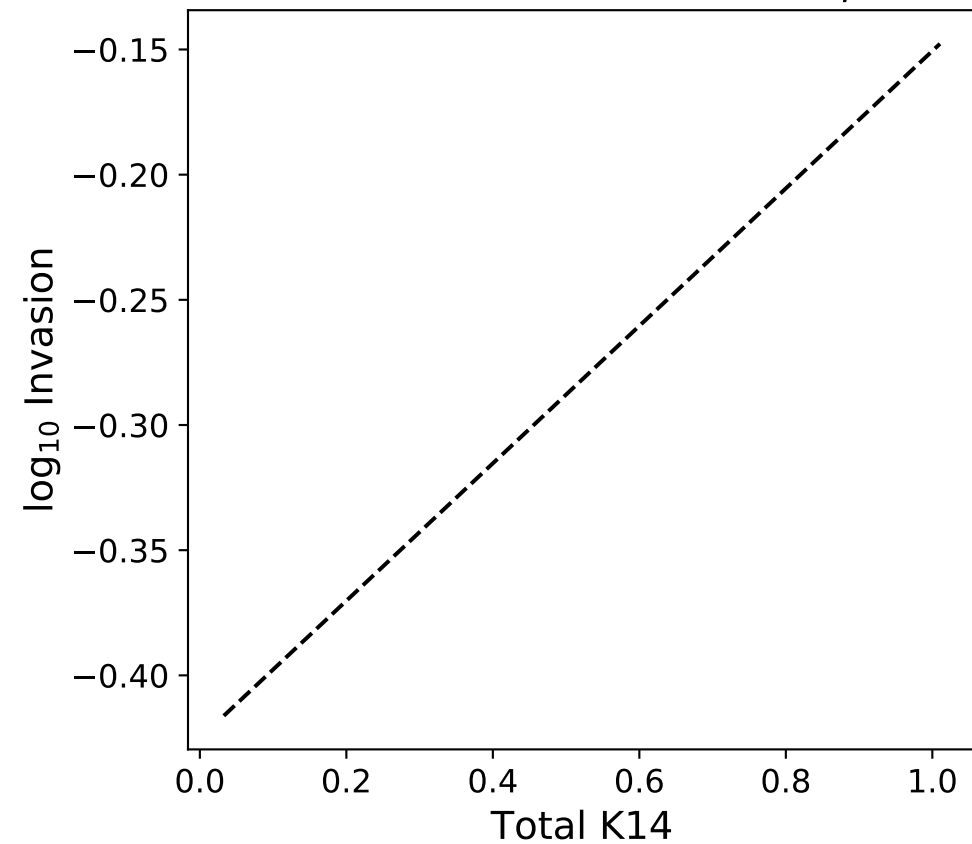

(B) Within-tumor test,  $R^2 = 0.22$ ,  $p = 2.3e-45$

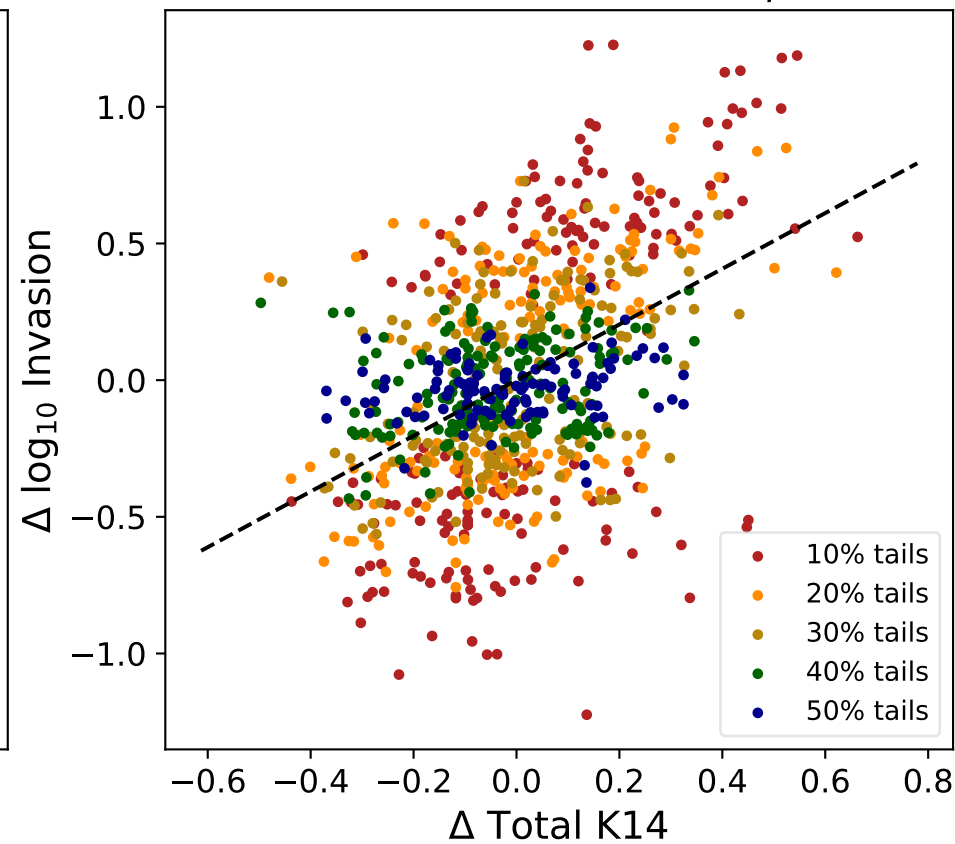

(C) Within-tumor test, extreme tails and pooling

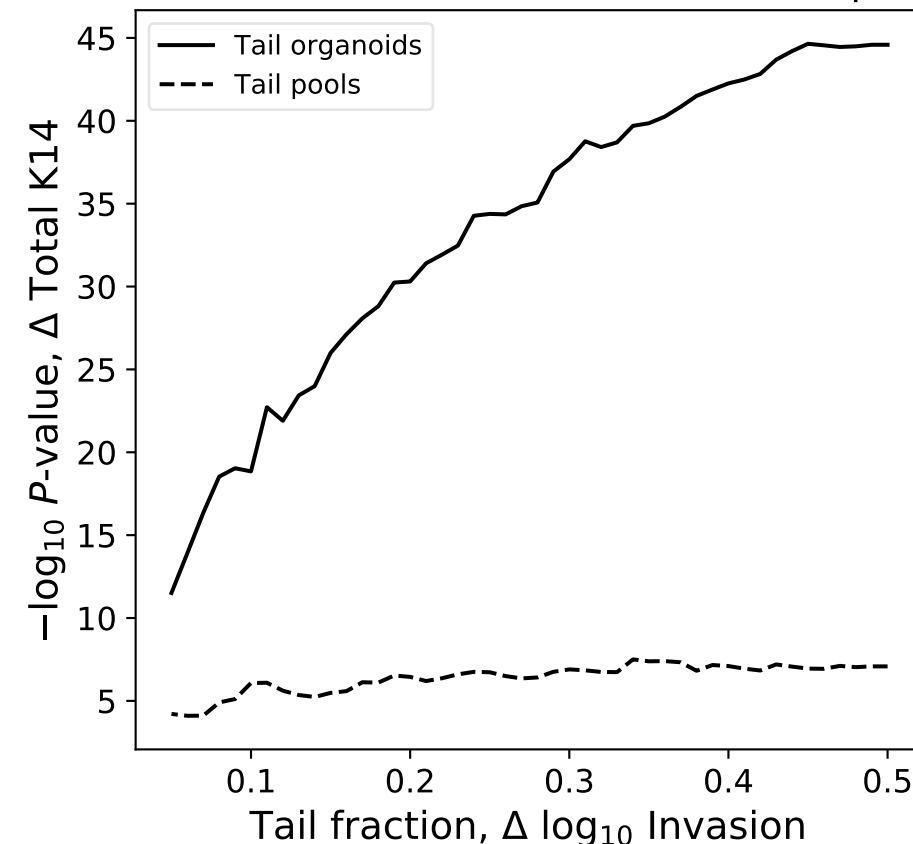

Supplement: S1 File — (GZ) [file pcbi.1007464.s003.tar.gz › S1_File/OUTPUT_ALL/fig9_k14total.pdf]

(A) Between-tumor test,  $R^2 = 0.15$ ,  $p = 0.008$

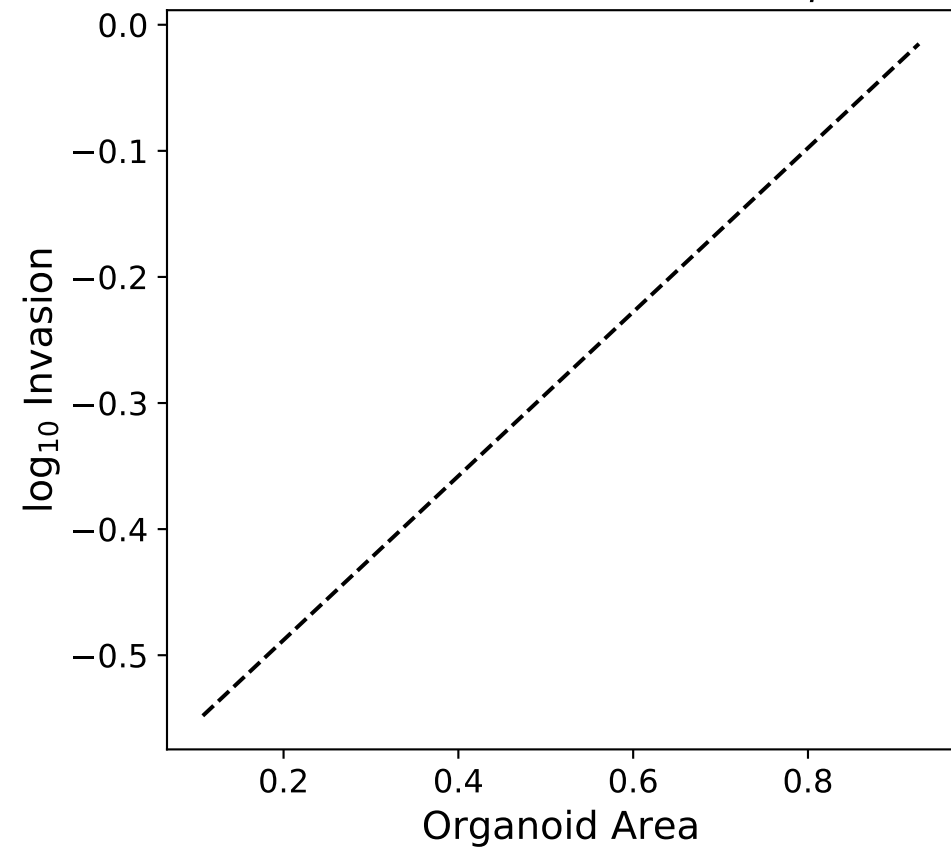

(B) Within-tumor test,  $R^2 = 0.25$ ,  $p = 9.8e-52$

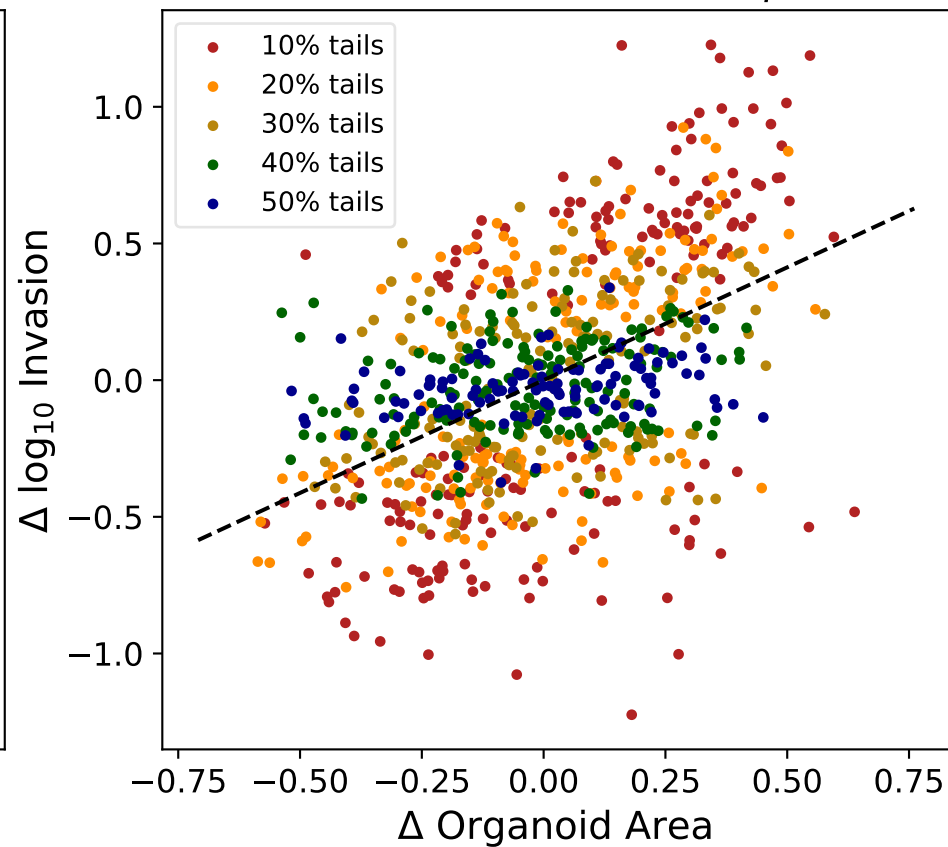

(C) Within-tumor test, extreme tails and pooling

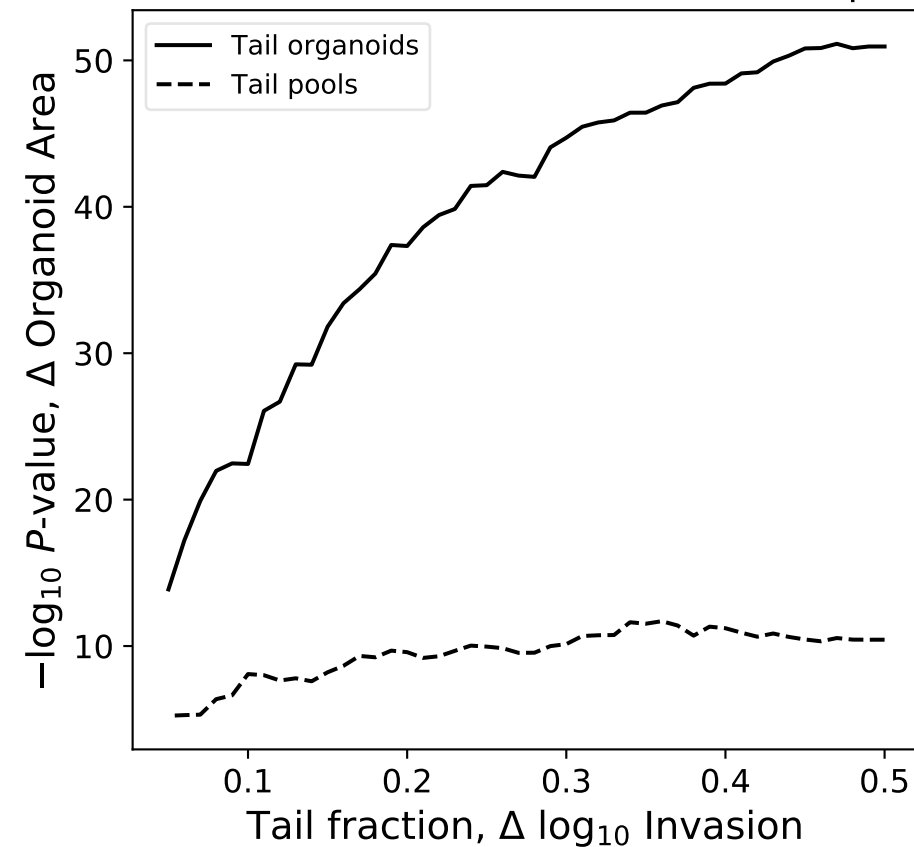

Supplement: S1 File — (GZ) [file pcbi.1007464.s003.tar.gz › S1_File/OUTPUT_ALL/fig11_area.pdf]

(A) size\_area, arithmetic scale

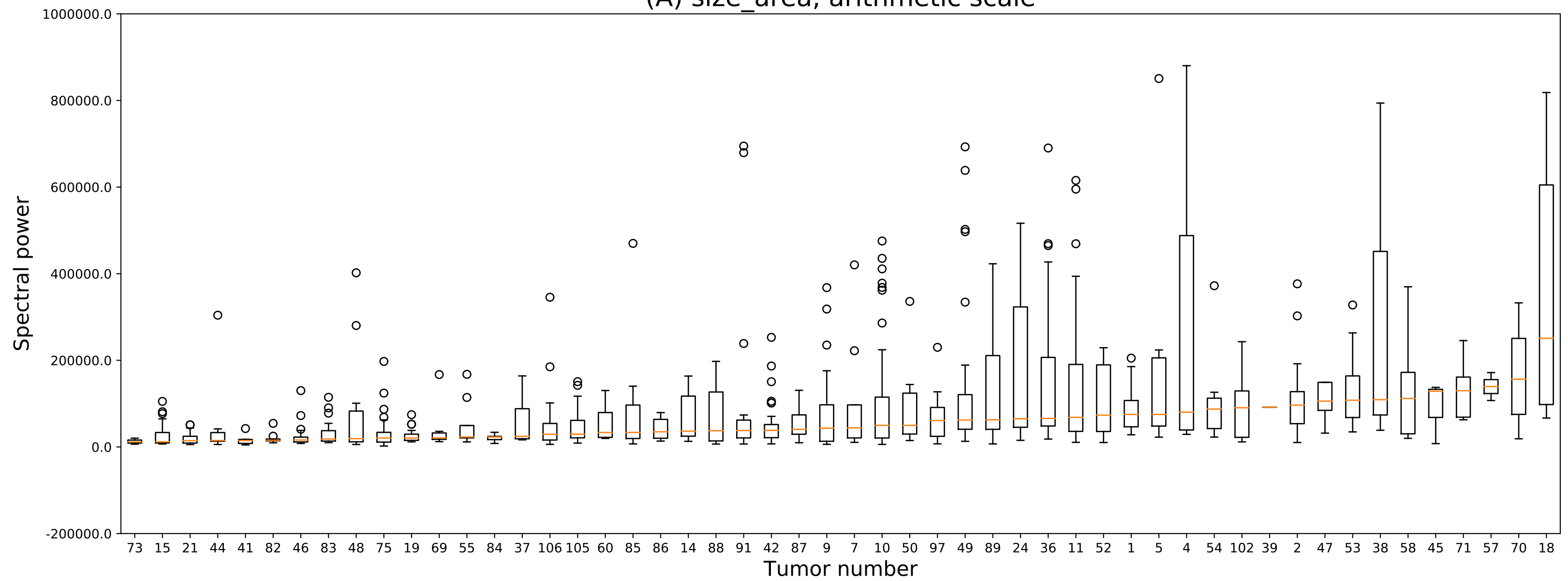

(B) size\_area, logarithmic scale

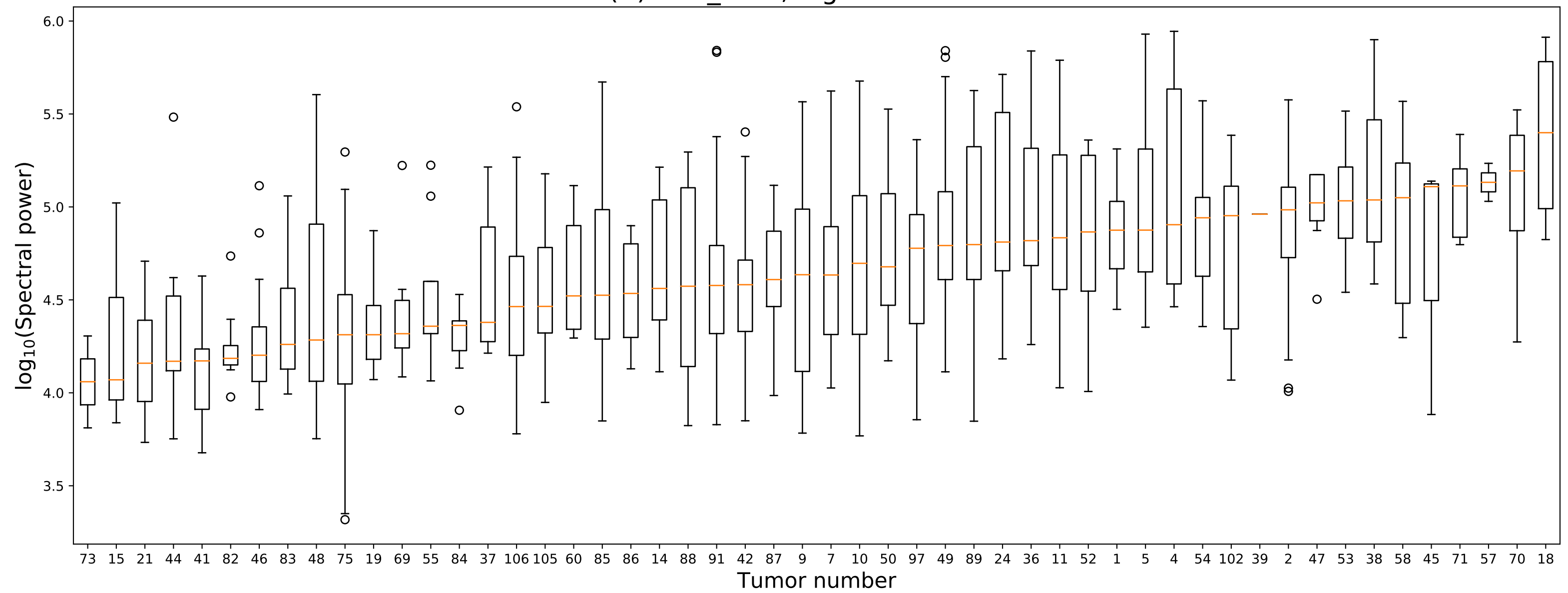

Supplement: S1 File — (GZ) [file pcbi.1007464.s003.tar.gz › S1_File/OUTPUT_ALL/boxplot_pair_size_area.pdf]

(A) Invasion, arithmetic scale

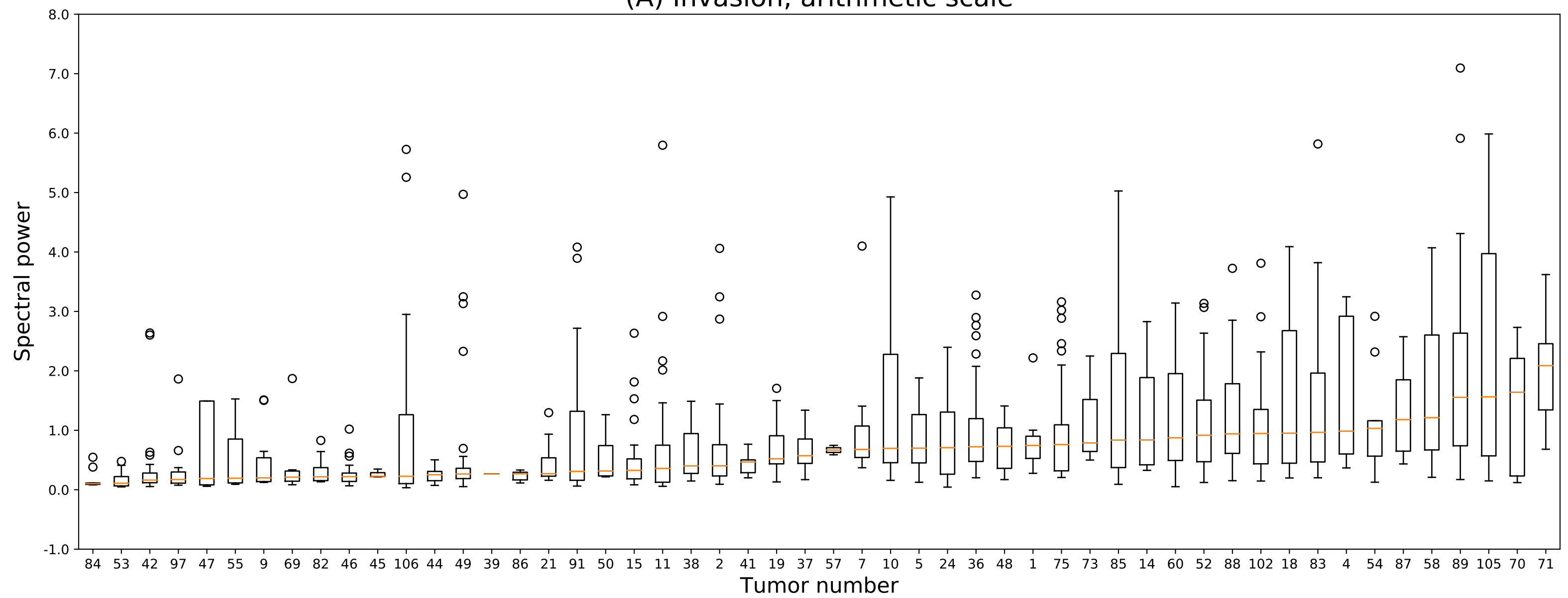

(B) Invasion, logarithmic scale

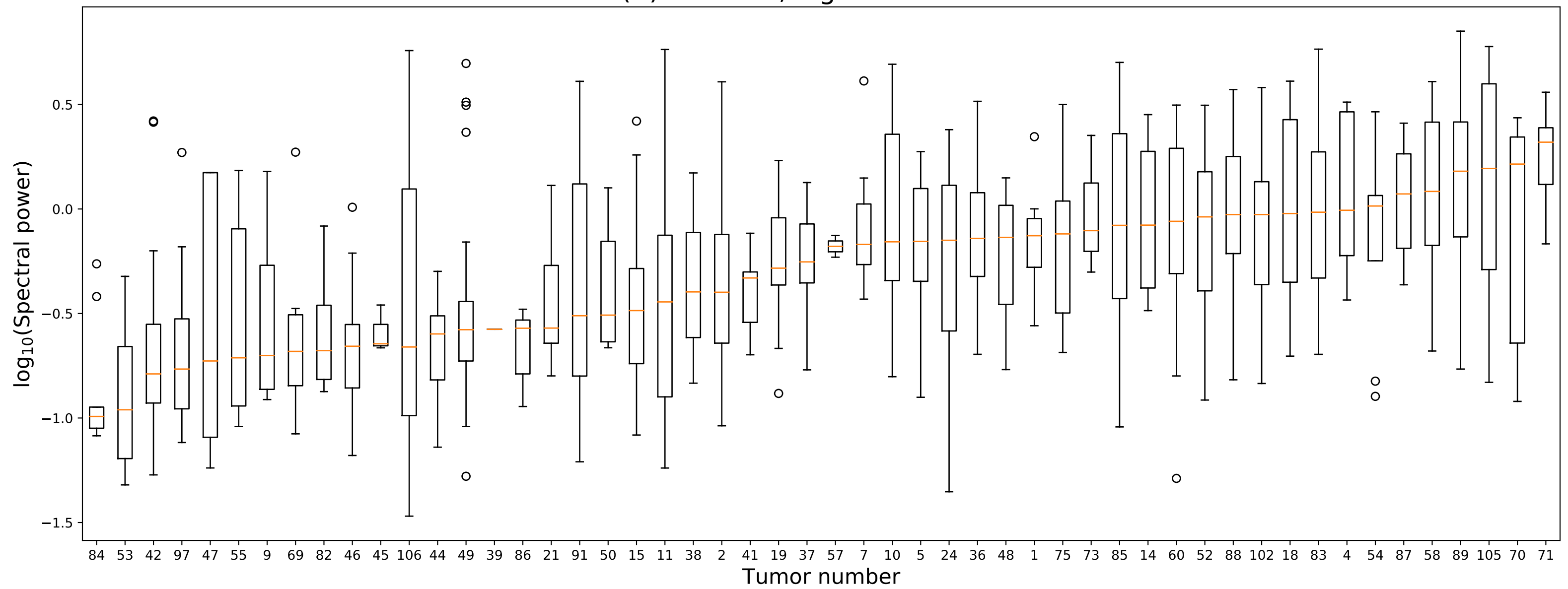

Supplement: S1 File — (GZ) [file pcbi.1007464.s003.tar.gz › S1_File/OUTPUT_ALL/boxplot_pair_Invasion.pdf]

(A) size\_perimeter, arithmetic scale

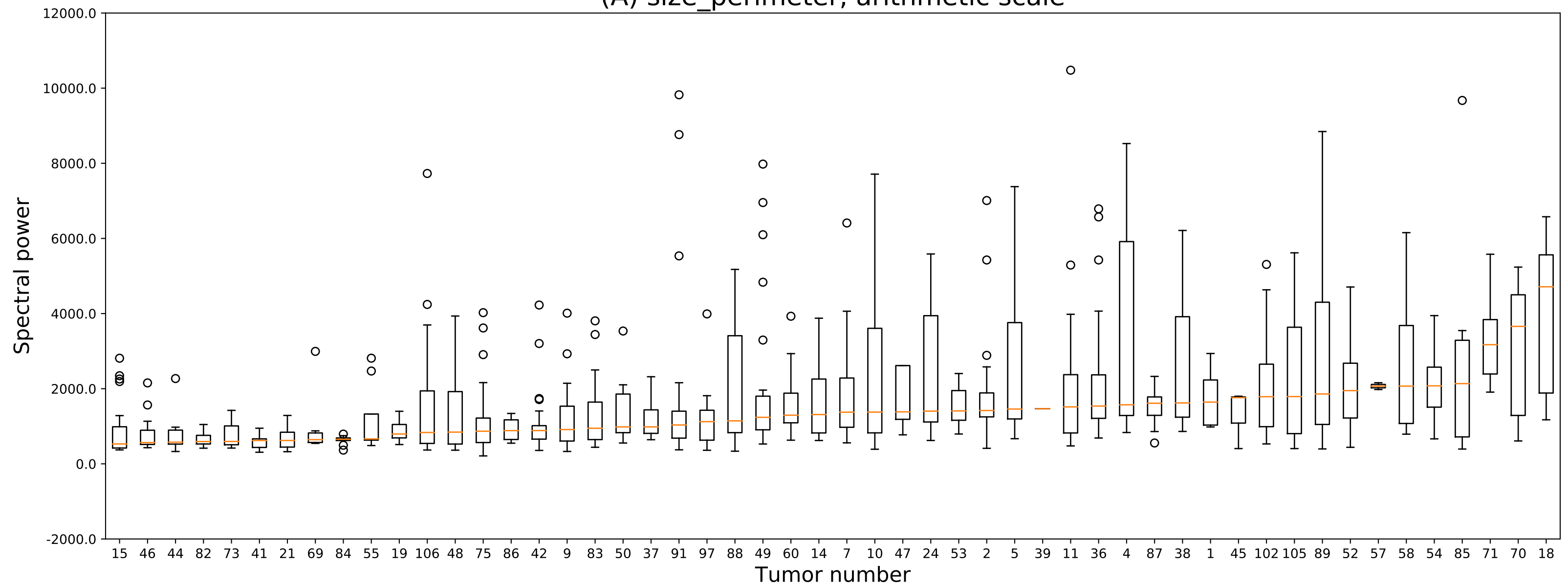

(B) size\_perimeter, logarithmic scale

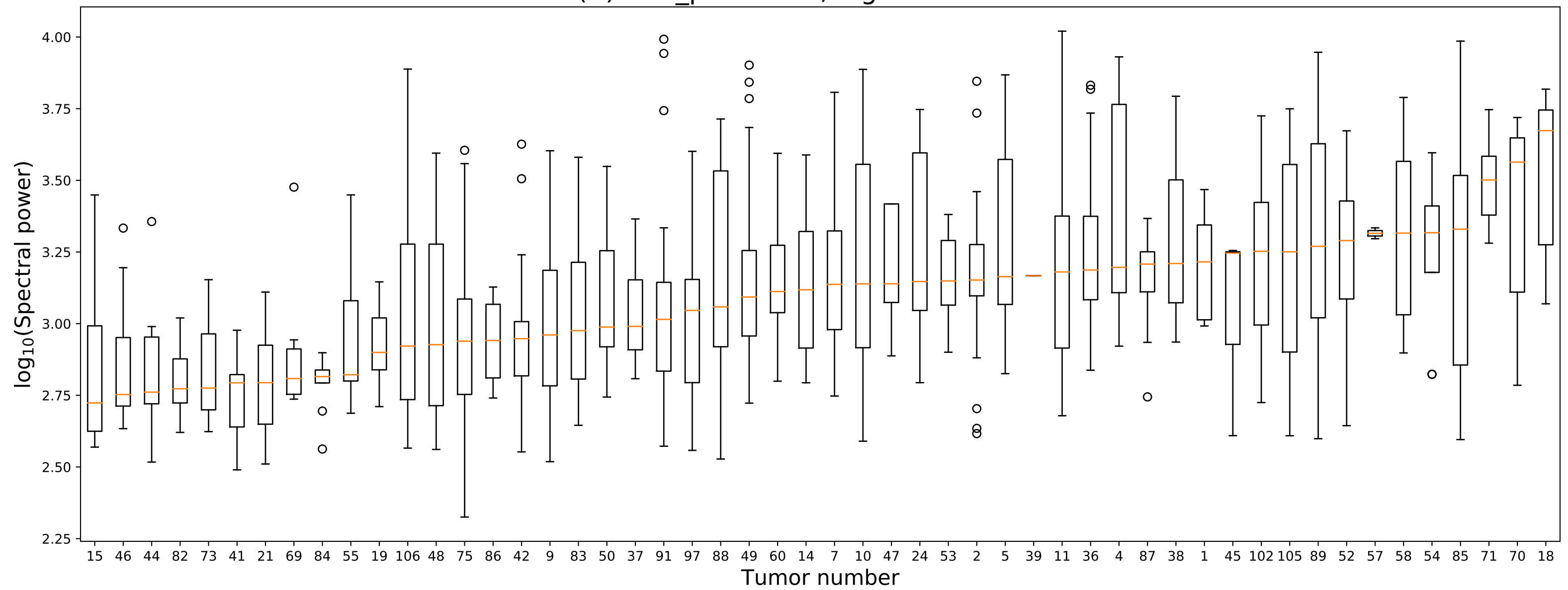

Supplement: S1 File — (GZ) [file pcbi.1007464.s003.tar.gz › S1_File/OUTPUT_ALL/boxplot_pair_size_perimeter.pdf]

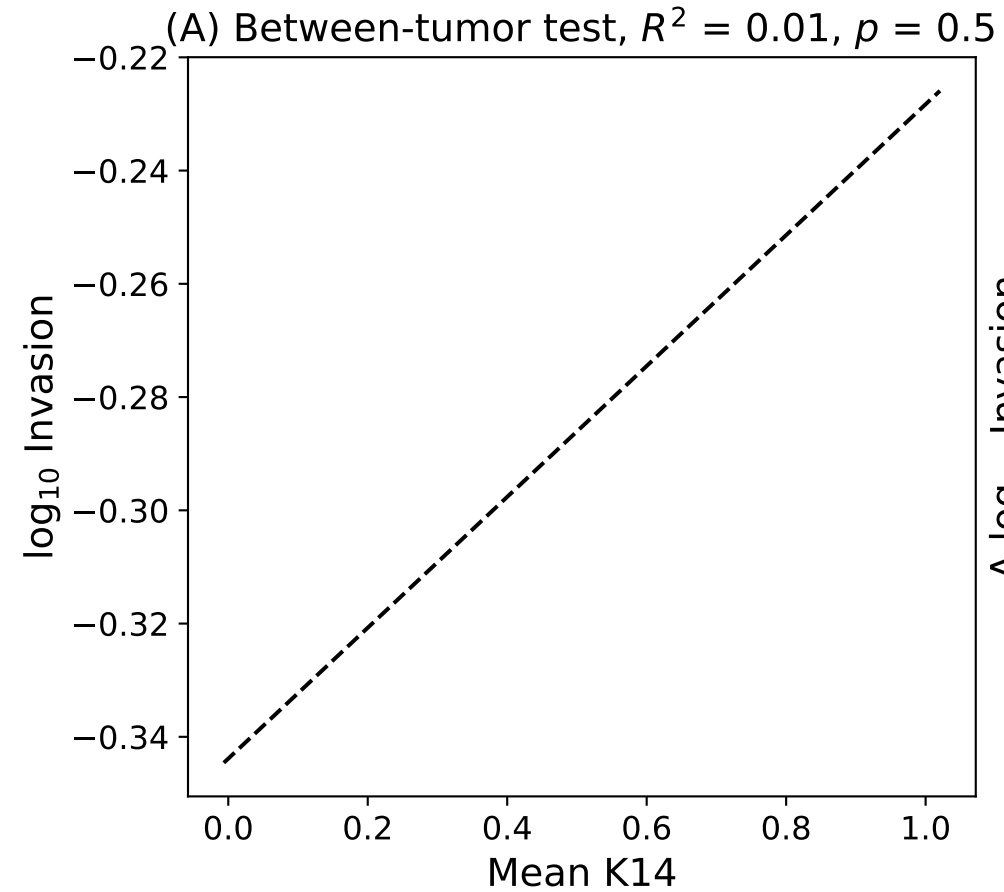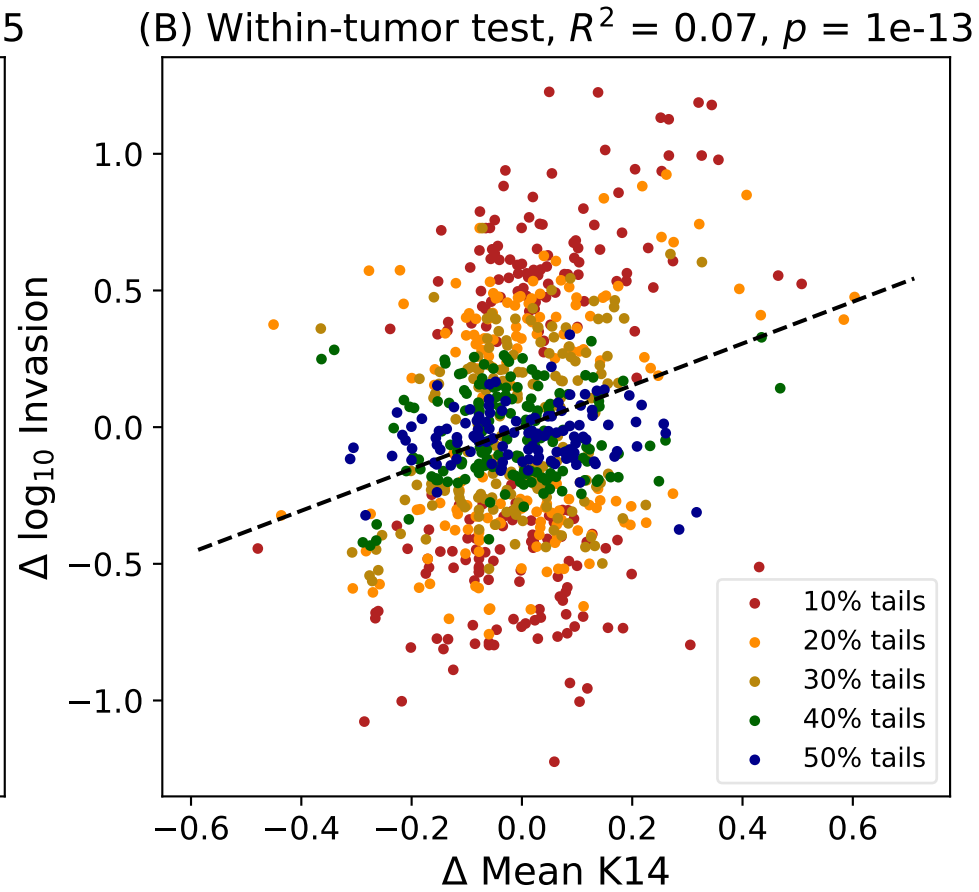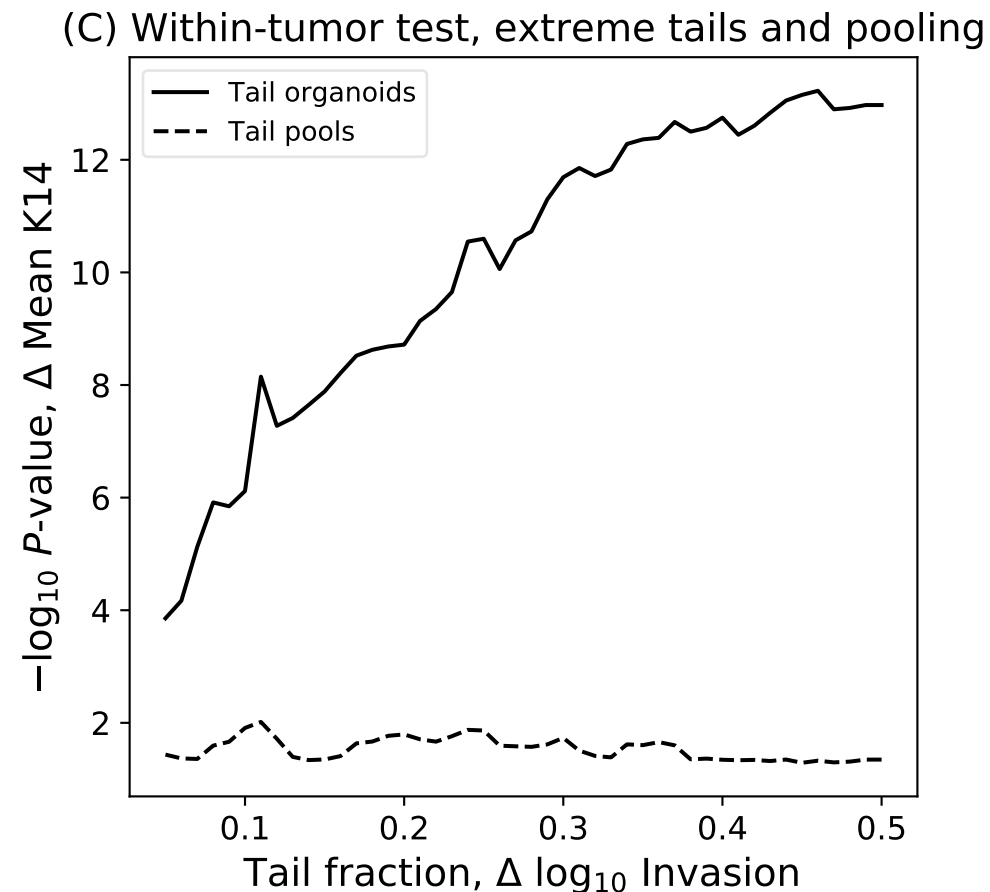

Supplement: S1 File — (GZ) [file pcbi.1007464.s003.tar.gz › S1_File/OUTPUT_ALL/fig10_k14mean.pdf]

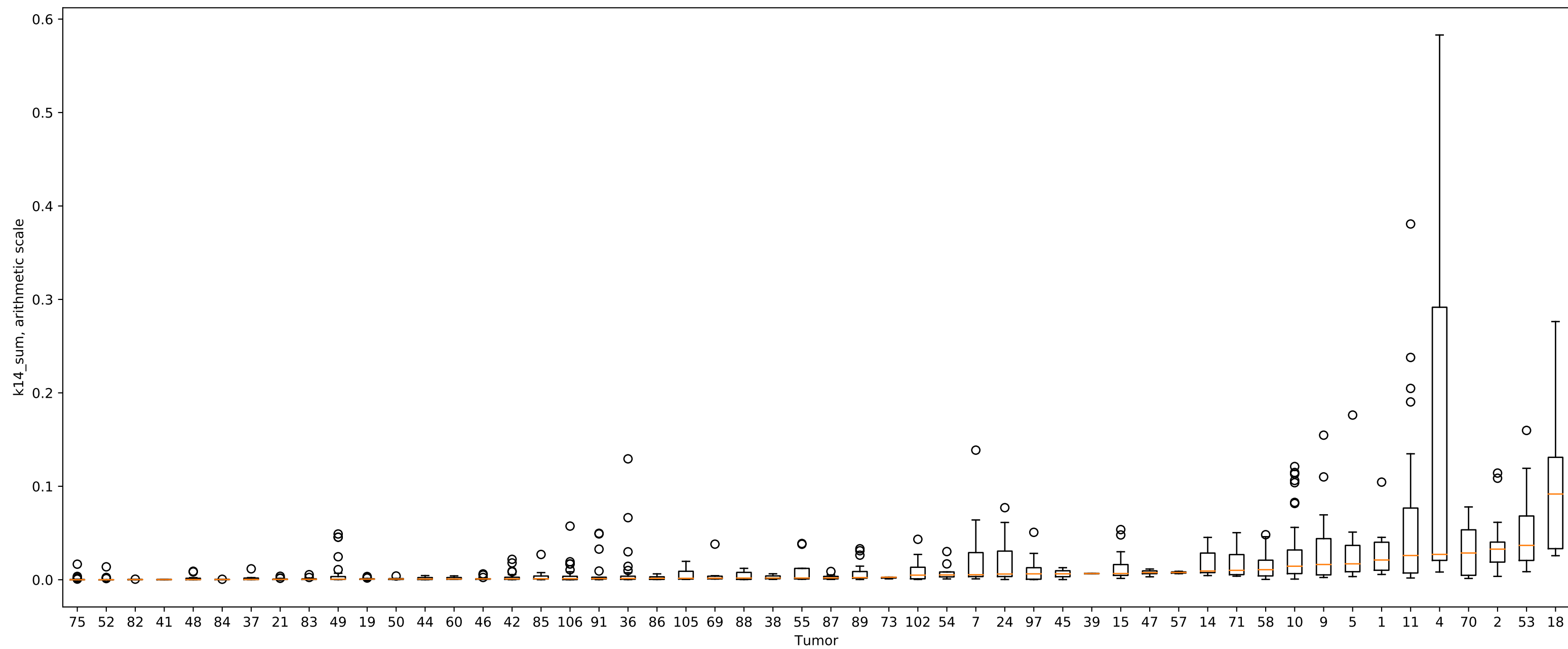

Supplement: S1 File — (GZ) [file pcbi.1007464.s003.tar.gz › S1_File/OUTPUT_ALL/boxplot_k14_sum.pdf]

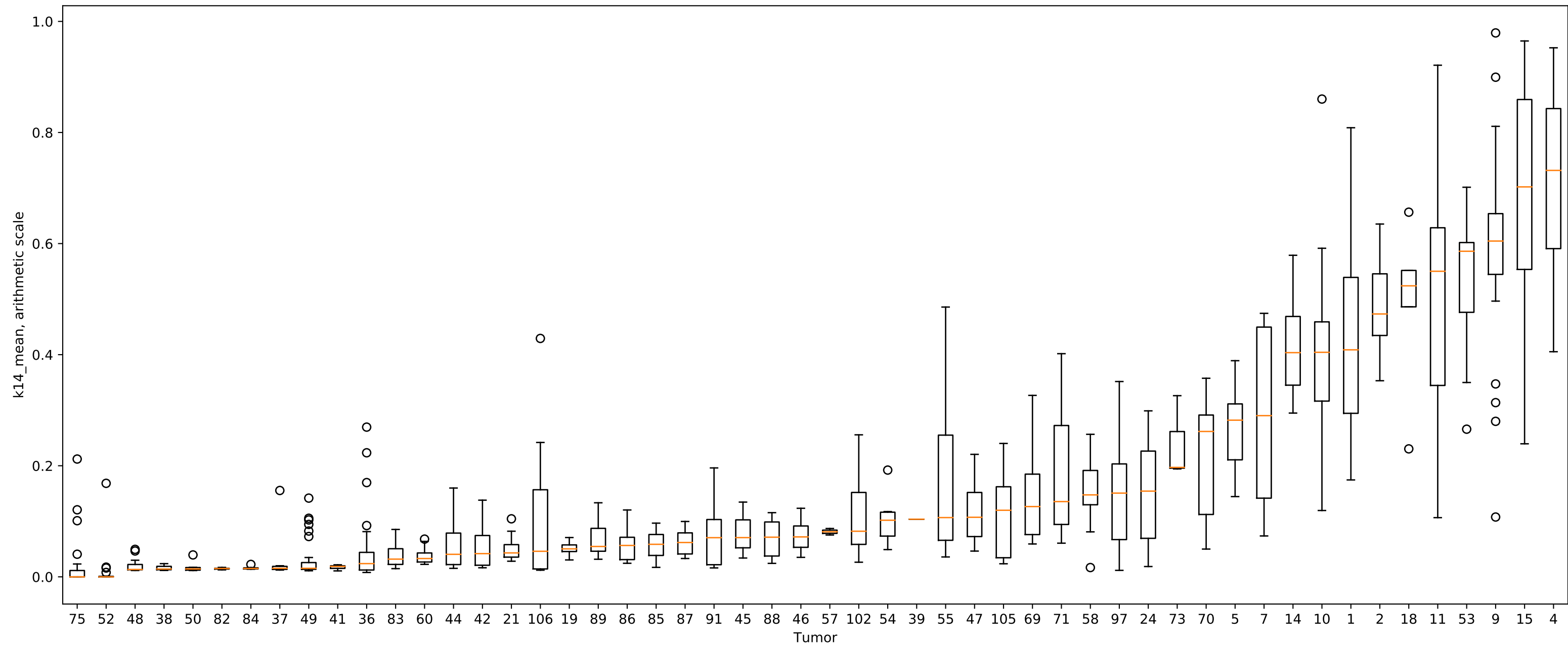

Supplement: S1 File — (GZ) [file pcbi.1007464.s003.tar.gz › S1_File/OUTPUT_ALL/boxplot_k14_mean.pdf]

(A) Total K14 per organoid

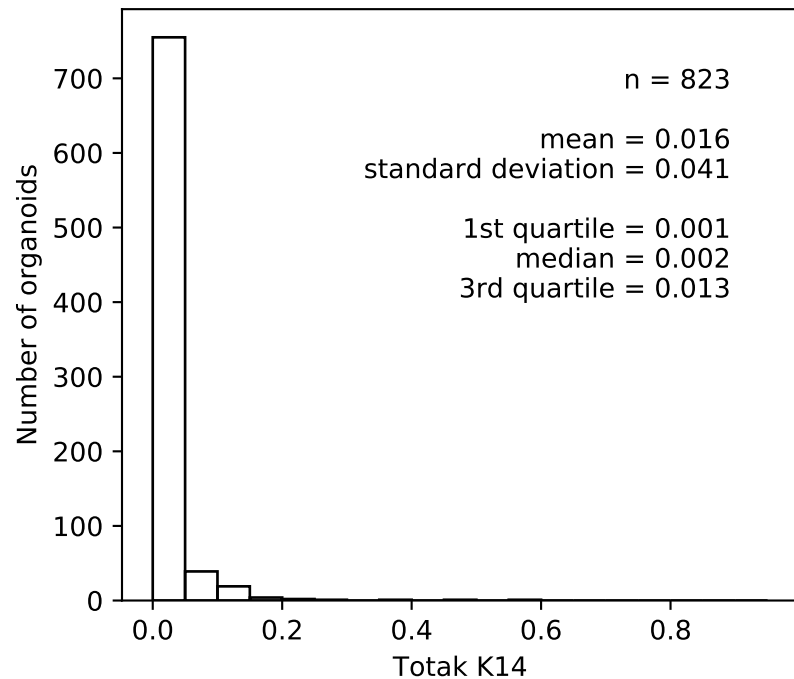

(B) Mean K14 per organoid

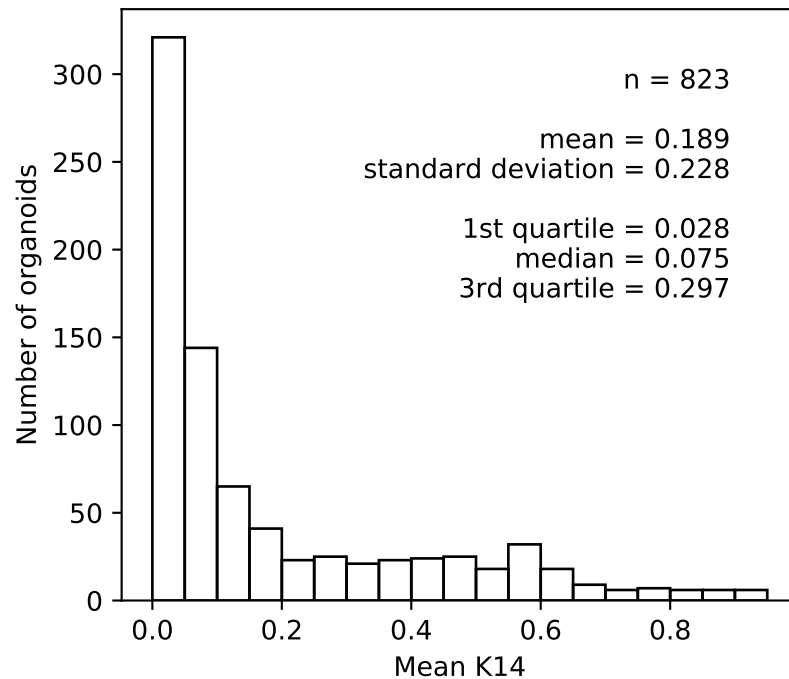

Supplement: S1 File — (GZ) [file pcbi.1007464.s003.tar.gz › S1_File/OUTPUT_ALL/fig8_histogram_k14.pdf]

(A) Manual boundary point distribution

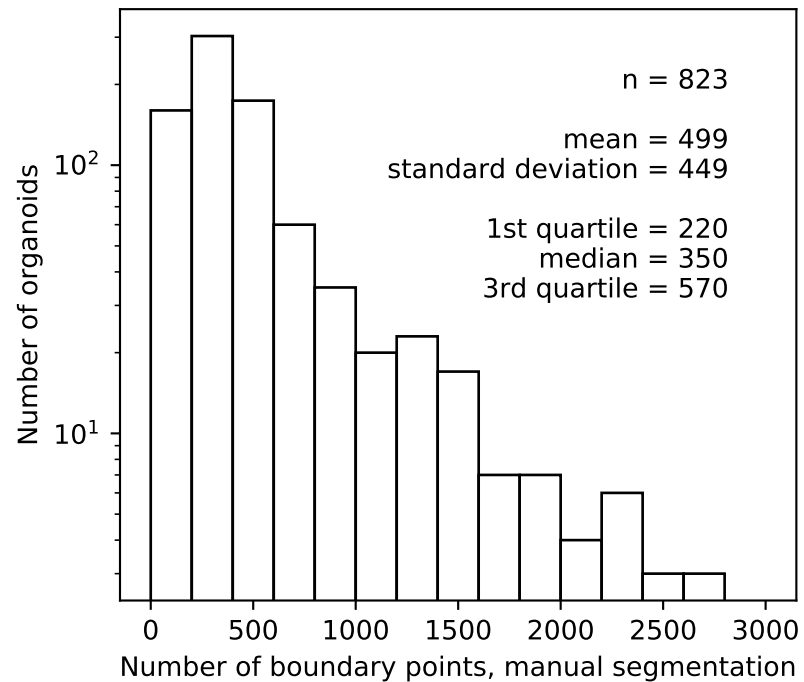

(B) Effective diameter distribution

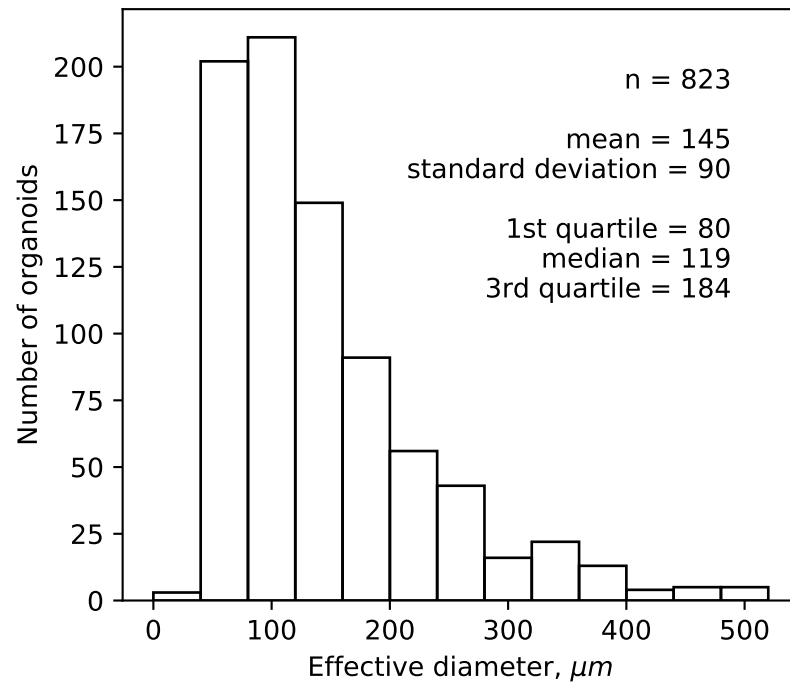

Supplement: S1 File — (GZ) [file pcbi.1007464.s003.tar.gz › S1_File/OUTPUT_ALL/fig1_histogram_pts_diam.pdf]
